# Acquisition of a side population fraction augments malignant phenotype in ovarian cancer

**DOI:** 10.1038/s41598-019-50794-w

**Published:** 2019-10-02

**Authors:** Koji Yamanoi, Tsukasa Baba, Kaoru Abiko, Junzo Hamanishi, Ken Yamaguchi, Ryusuke Murakami, Mana Taki, Yuko Hosoe, Susan K. Murphy, Ikuo Konishi, Masaki Mandai, Noriomi Matsumura

**Affiliations:** 1 Department of Gynecology and Obstetrics, Kyoto University Graduate School of Medicine, Kyoto, Japan; 2 Department of Obstetrics and Gynecology, National Hospital Organization Kyoto Medical Center, Kyoto, Japan; 3 Division of Gynecologic Oncology, Department of Obstetrics and Gynecology, Duke University Medical Center, Durham, USA; 4 Department of Obstetrics and Gynecology, Faculty of Medicine, Kindai University, Osaka, Japan

**Keywords:** Cancer genomics, Ovarian cancer

## Abstract

Side population (SP) cells harbor malignant phenotypes in cancer. The aim of this study was to identify genes that modulate the proportion of ovarian cancer SP cells. Using a shRNA library targeting 15,000 genes, a functional genomics screen was performed to identify genes whose suppression increased the SP percentage. The biological effects caused by alteration of those identified genes were investigated *in vitro* and *in vivo*. We found that suppression of *MSL3*, *ZNF691*, *VPS45*, *ITGB3BP*, *TLE2*, and *ZNF498* increased the proportion of SP cells. Newly generated SP cells exhibit greater capacity for sphere formation, single cell clonogenicity, and *in vivo* tumorigenicity. On the contrary, overexpression of *MSL3*, *VPS45*, *ITGB3BP*, *TLE2*, and *ZNF498* decreased the proportion of SP cells, sphere formation capacity and single cell clonogenicity. In ovarian cancer cases, low expression of *MSL3*, *ZNF691* and *VPS45* was related to poor prognosis. Suppression of these six genes enhanced activity of the hedgehog pathway. Cyclopamine, a hedgehog pathway inhibitor, significantly decreased the number of SP cells and their sphere forming ability. Our results provide new information regarding molecular mechanisms favoring SP cells and suggest that Hedgehog signaling may provide a viable target for ovarian cancer.

## Introduction

Ovarian cancer is the most lethal cancer among gynecologic malignancies^1^ and is usually diagnosed at an advanced stage with peritoneal dissemination^2^. Most ovarian cancer patients respond well to primary chemotherapy and enter into clinical remission. However, tumor recurrence occurs in more than 70% of patients^3,4^. Recurrent tumors are usually resistant to treatment, resulting in poor prognosis^5,6^.

The cellular side population (SP) is a subpopulation of cells defined by the ability to efflux DNA dye Hoechst 33342 and can be detected using flow cytometry^7^. Recent reports have shown that the SP is identified in a variety of solid tumor types including ovarian cancer^8,9^. It is known that SP cells are chemoresistant, have sphere forming capacity, exhibit single cell clonogenicity, and *in vivo* tumorigenicity^8,9^. In addition, in ovarian cancer, a relatively high proportion of SP cells are found in samples from clinical relapse^10^, and the proportion of SP cells is said to be an independent factor for poor prognosis^11^. However, little is known about the factor(s) that regulate the proportion of SP cells in cancer. If these factors are identified, they may help us to establish new therapeutic approaches for treatment of refractory ovarian cancer.

A functional genomics screen is a very useful tool to identify factors that re strongly related to specific functions or phenotypes among the numerous genetic alterations occurring in cancer cells. A shRNA library is one of the most useful methods to perform a functional genomics screen^12^. We previously performed a functional genomics screen by shRNA library, and successfully identified some novel genes that contribute to anoikis resistance in ovarian cancer^13^.

In the present study, we report identification of novel factors whose downregulation markedly increases the proportion of SP cells in ovarian cancer. To our best knowledge, this is the first report of functional genomis screen to find shRNAs that increase the proportion of SP cells in cancer cells.

## Results

### Functional genomic screen

**Figure 1:**
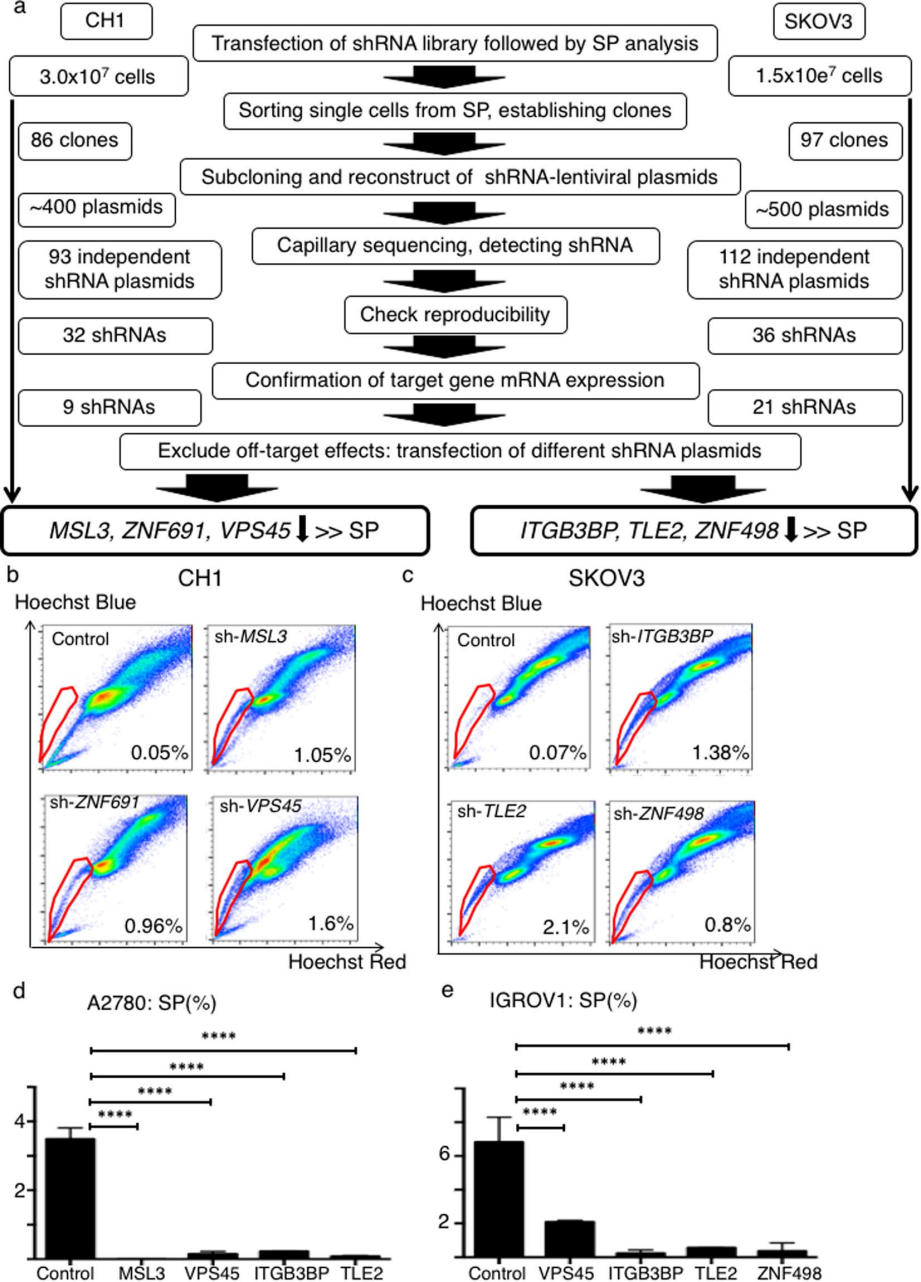
Schematic of functional genomics screening. (**a**) Ovarian cancer cell lines, CH1 and SKOV3 were used. Following transfection of the shRNA library, we performed SP analysis and SP cells were singly plated into each well of 96-well plates. We established 86 clones in the CH1 screen and 97 clones in the SKOV3 screen. shRNAs were amplified by PCR and we reconstructed 93 different shRNA plasmids from the CH1 screen and 115 different shRNA plasmids from the SKOV3 screen. Out of 97 shRNAs in CH1 cells, 32 again markedly increased the SP fraction. Out of 115 shRNAs in SKOV3 cells, 36 again markedly increased the SP fraction. We measured mRNA expression of these 32 and 36 genes using RT-PCR, and found that 9 shRNAs in CH1 suppressed their target gene’s mRNA expression and 21 shRNAs in SKOV3 suppressed their target gene’s mRNA expression. We transfected shRNAs containing completely different anti-sequence than those initially used for each gene and performed SP analysis and RT-PCR to exclude off target effects. We identified *MSL3*, *ZNF691* and *VPS45*, whose downregulation markedly increased the SP fraction of CH1 cells and *ITGB3BP*, *TLE2* and *ZNF498* whose downregulation markedly increased the SP fraction of SKOV3 cells. (**b**) Representative data showing the percentage of the SP fraction of control, sh-*MSL3*, sh-*ZNF691* and sh-*VPS45* CH1 cells. (**c**) Representative data showing the percentage of the SP fraction of control, sh-*ITGB3BP*, sh-*TLE2* and sh-Z*NF498* SKOV3 cells. (**d**) SP fraction percentage of A2780-control, orf-MSL3, orf-VPS45, orf-ITGB3BP and orf-TLE2. ****p < 0.0001. (**e**) SP fraction percentage of IGROV1-control, orf-VPS45, orf-ITGB3BP, orf-TLE2 and orf-ZNF498. ****p < 0.0001

A schematic of the functional genomics screen is shown in Fig. 1a. We used two human ovarian cancer cell lines, CH1 and SKOV3, which harbor few SP cells (Supplementary Fig. S1). We transfected the shRNA library into these two cell lines followed by SP analysis. The identified SP cells were single-cell-sorted and independently expanded. Then we identified 93 kinds of anti-sequences in the screen from CH1 (see Supplementary Table S1a), and 112 kinds of anti-sequences in the screen using SKOV3 (see Supplementary Table S1b). We transfected the reconstructed shRNA plasmids into CH1 or SKOV3 cells individually followed by SP analysis and qPCR. We found that nine of the shRNA plasmids for CH1 and 21 of the shRNA plasmids for SKOV3 markedly suppressed expression of their target genes (Supplementary Figs S2, S3).

We next transfected shRNA plasmids containing a different anti-sense sequence against the same gene followed by SP analysis and q-PCR. We assumed that off-target effects would be evident if among two shRNAs targeting the same gene, both increase the SP but only one exhibits target gene suppression, or when one shRNA does not increase the SP but does suppress expression of the target gene. As a result, we identified three genes, *MSL3*, *ZNF691*, and *VPS45*, whose downregulation markedly increased the proportion of SP cells for CH1 (Fig. 1b, Supplementary Figs S4a, S5a). We also identified three other genes, *ITGB3BP*, *TLE2*, and *ZNF498*, whose downregulation markedly increased the SP fraction for SKOV3 (Fig. 1c, Supplementary Figs S4b, S5b).

We investigated whether overexpression of these six genes decreased the proportion of SP cells. We used two other human ovarian cancer cell lines, A2780 and IGROV1, that each harbor some SP cells (Supplementary Fig. S1). We transfected the open reading frame of the six genes independently into the A2780 and IGROV1 cells to establish overexpression of the identified target genes. For A2780, we established *MSL3*, *VPS45*, *ITGB3BP*, and *TLE2*-overexpressing cells (Supplementary Fig. S5c). For IGROV1, we established *VPS45*, *ITGB3BP*, *TLE2*, and *ZNF498*-overexpressing cells (Supplementary Fig. S5d). As for ZNF691, we transfected its open reading frame to both A2780 and IGROV1, but we could not confirm overexpression of ZNF691 mRNA expression in both open reading frame-transfected A2780 and IGROV1 cells. Then we quantified the SP proportion of the overexpressing cells relative to control cells. Although *ZNF691*-overexpressing cells was not established, overexpression of the other five genes significantly decreased the proportion of A2780 and IGROV1 SP cells as compared to the control cells (Fig. 1d,e).

In summary, using a functional genomics screen, we identified six novel genes, *MSL3*, *ZNF691*, *VPS45*, *ITGB3BP*, *TLE2*, and *ZNF498*, that regulate the proportion of SP cells in ovarian cancer.

### Analysis of clinical samples

To our knowledge, there are few reports that describe the function of these six SP-regulating genes. In particular, little is known about their functions related to cancer (Table 1). We investigated the expression status of these genes in ovarian cancer clinical samples.

**Table 1:** Gene lists detected by functional genomics screen.

Symbol	Full name	Location	Reported function
MSL3	male-specific leathal 3 homolog	Xp22.2	Chromatin remodeling and transcriptional regulation
ZNF691	zing finger protein 691	1p34.2	Unknown
VPS45	vacuolar protein sorting 45 homolog	1q21.2	Playing a role in trafficking proteins and autoohagy
ITGB3BP	integrin subunit beta 3 binding protein	1p31.3	Enhance the activity of members of nuclear receptor families, thyroid hormone receptor. Corepressor of NF-kappaB-dependent signaling.
TLE2	transducin like enhancer of split 2	19p13.3	Co-reppressor of Wnt pathway
ZNF498	zing finger protein 498	7q22.1	Unknown

**Figure 2:**
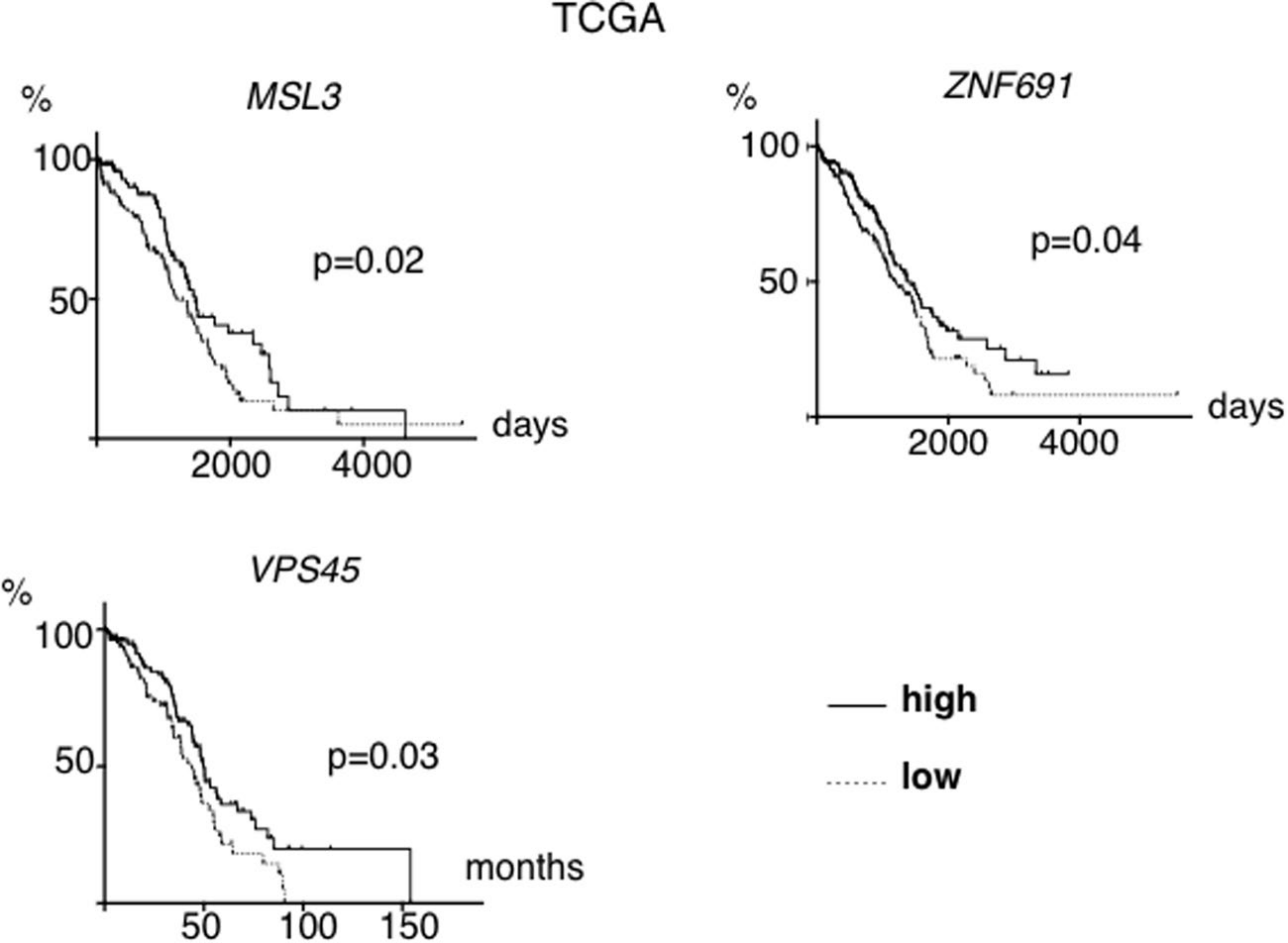
Overall survival based on mRNA expression of *MSL3*, *ZNF691* and *VPS45* in clinical samples. TCGA cases were analyzed. Differences in survival based on *MSL3*, *ZNF691* and *VPS45* mRNA expression are shown. The low expression group consisted of 100 cases with the lowest levels of expression while the high expression group consisted of 100 cases with the highest levels of expression.

First, we analyzed overall survival. Using clinical data from The Cancer Genome Atlas (TCGA), we compared overall survival between the low mRNA expression group (100 patients with lowest expression) with that of the high mRNA expression group (100 patients with highest expression) for each one of the six genes. Survival for patients with tumors having low expression of *MSL3*, *VPS45*, and *ZNF691* was significantly shorter than that of the high expression group (Fig. 2), although no significant differences were found for the other three genes. Using another ovarian cancer clinical dataset, (GSE3149), the survival for patients with tumors having low expression of *MSL3* and *ITGB3BP* was again significantly shorter than that of the high expression group (Supplementary Fig. S6a,b, p < 0.05 for each).

Additionally, we analyzed association of the six genes with genomic deletion, metastasis, relapse, and chemoresistance. Genomic deletion at *TLE2* (19p13.3) was frequent in ovarian cancer (84%, Supplementary Fig. S6c). *ZNF498* expression at the metastatic site was significantly lower than that at the primary site (Supplementary Fig. S6d, p = 0.01). *MSL3* expression derived from the relapsed tumors was significantly lower than that derived from the primary tumors (Supplementary Fig. S6e, p = 0.01, n = 17, Supplementary Fig. S6f, p = 0.0012, n = 12). Expression of *VPS45* and *ZNF498* derived from the relapsed tumors were also significantly lower than that derived from the primary tumors (Supplementary Fig. S6g,h, p = 0.036, p = 0.013, respectively). *ITGB3BP* expression derived from the paclitaxel-resistant relapsed tumors was significantly lower than that derived from the primary tumors (Supplementary Fig. S6i, p = 0.04, n = 6). Collectively, these analyses suggest clinical relevance with the identified genes in ovarian cancer.

### Alteration of sphere forming capacity, single cell clonogenicity and cell morphology caused by expression alteration of the six genes

**Figure 3:**
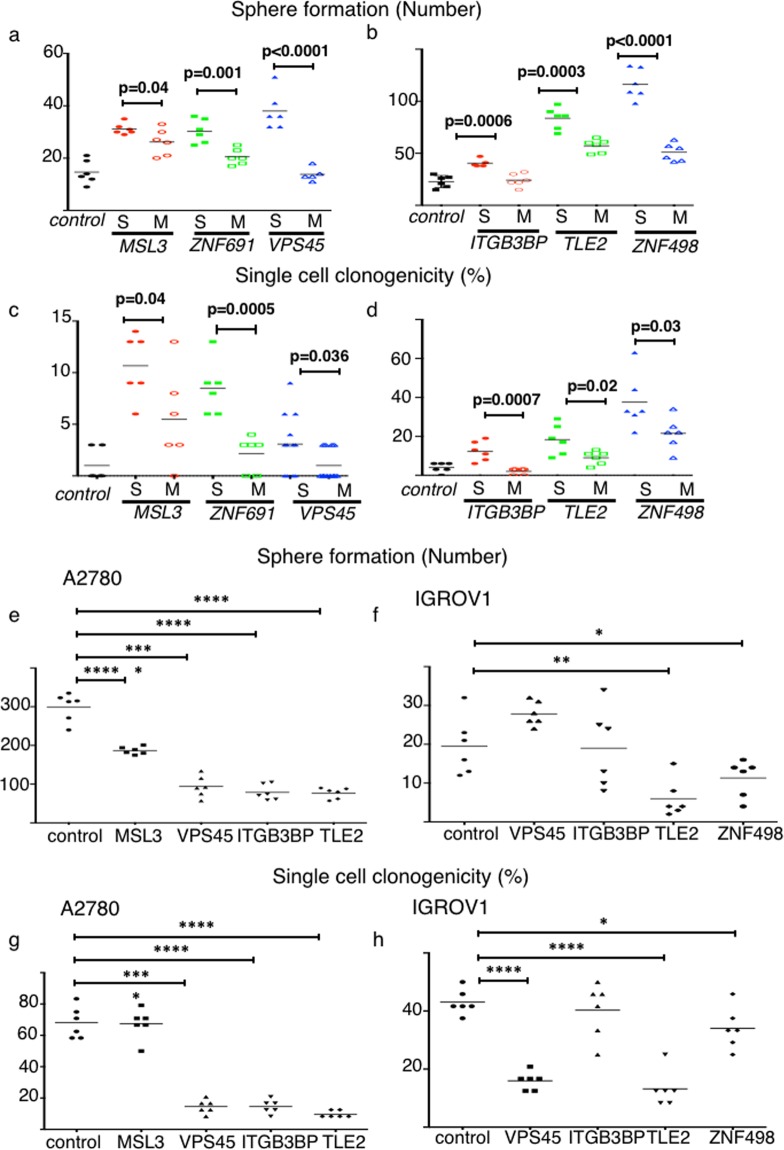
Six genes regulate cancer stem cell (CSC) phenotypes as well as the SP fraction. Sphere formation and single cell clonogenicity were assessed. (**a**–**d**) Newly generated SP cells harbor a CSC phenotype. (**a**,**b**) Sphere forming ability. Data from CH1-shcontrol and sh-target gene cells are shown in Fig. 3a; data from SKOV3-shcontrol and sh-target gene cells are shown in Fig. 3b. The number represents spheres larger than 200 µm (n = 6 replicates). (**c**,**d**) Single cell clonogenicity. Expanded clone colonies were counted and the proportion of cells capable of colony formation was calculated (n = 6 replicates). Data from CH1-shcontrol and sh-target gene cells are shown in Fig. 3c; data from SKOV3-shcontrol and sh-target gene cells are shown in Fig. 3d. (**e**–**h**) Overexpression of *MSL3*, *VPS45*, *ITGB3BP*, *TLE2*, *ZNF498* decreased sphere formation and single cell clonogenicity. (**e**,**f**) Sphere formation. Data from A2780-control and ORF-target gene cells are shown in Fig. 3e. Data from IGROV1-control and ORF-target gene cells are shown in Fig. 3f. The number of spheres larger than 200 µm is depicted. *p < 0.05, **p < 0.01, ***p < 0.001, ****p < 0.0001. (**g**,**h**) Single cell clonogenicity. Expanded clones were counted and the proportion of cells capable of colony formation was calculated. Data from A2780-control and ORF-target gene cells are shown in Fig. 3g. Data from IGROV1-control and ORF-target gene cells are shown in Fig. 3h. *p < 0.05, **p < 0.01, ***p < 0.001, ****p < 0.0001.

It is widely known that SP cells have pro-malignant phenotypes such as sphere formation ability and single cell clonogenicity^8^. We checked sphere forming capacity of SP cells that were newly generated by suppression of the six genes. As a result, newly generated SP cells had significantly higher sphere formation as compared to the main population (MP) cells and control cells (Fig. 3a,b, p < 0.05, respectively). Next, we examined single cell clonogenicity. Newly-generated SP cells had significantly higher single cell clonogenicity as compared to the MP cells and control cells (Fig. 3c,d, p < 0.05, respectively).

Next, we investigated whether overexpression of the six genes caused a change in sphere formation and single cell clonogenicity. For A2780, we established *MSL3*, *VPS45*, *ITGB3BP*, and *TLE2*-overexpressing cells (Supplementary Fig. S5c). For IGROV1, we established *VPS45*, *ITGB3BP*, *TLE2*, and *ZNF498*-overexpressing cells (Supplementary Fig. S5d). Using those cell lines, we performed experiments. For the A2780 cells, overexpression of *MSL3*, *VPS45*, *ITGB3BP* and *TLE2* significantly decreased sphere formation compared to the control cells (p < 0.0001, Fig. 3e). Overexpression of three genes, *VPS45*, *ITGB3BP* and *TLE2* significantly decreased single cell clonogenicity as compared to the control cells (p < 0.0001, Fig. 3g). Using the IGROV1 cells, we found that overexpression of *TLE2* and *ZNF498* significantly decreased sphere formation as compared to the control cells (p < 0.05, respectively, Fig. 3f). Overexpression of three genes, *VPS45*, *TLE2*, and *ZNF498* significantly decreased single cell clonogenicity relative to the control cells (p < 0.05, respectively, Fig. 3h).

Intriguingly, altered expression of the six genes changed *in vitro* cell morphology. Suppression of six genes tend to lead to a cobblestone appearance. On the contrary, overexpression changed to spindle (Supplementary Fig. S7).

### Alteration of *in vivo* tumorigenicity, *in vivo* tumor proliferation and morphology caused by altered expression of the six genes

**Figure 4:**
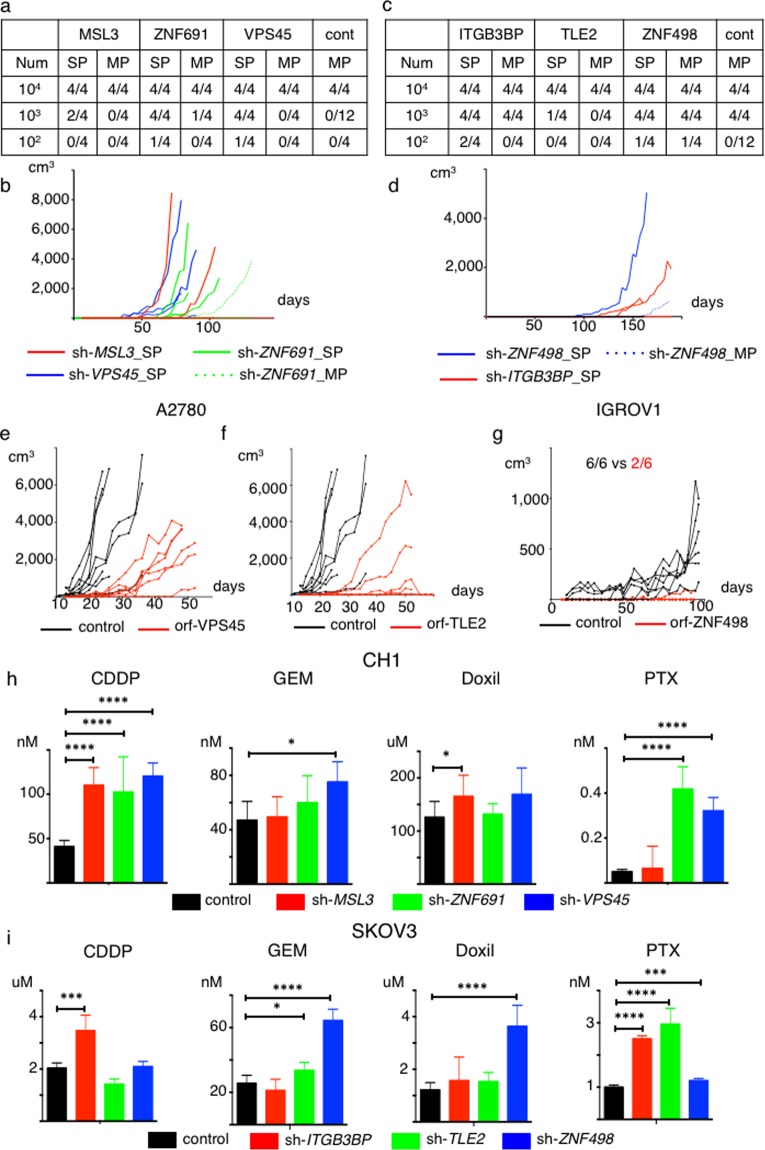
Target genes regulate tumor initiating ability and *in vivo* tumor proliferation, and suppression of six genes enhances resistance to chemotherapeutic agents. (**a**,**b**) Newly generated SP cells exhibit greater tumor initiating capability than do MP cells and control cells. Data from CH1-control, SP and MP cells derived from CH1 sh-target gene cells are shown. Num; Number, cont; control-MP. (**a**) Limiting dilution assay results. (**b**) Tumor growth curves generated from injection of 1 × 10^3^ indicated cells. (**c**,**d**) Newly generated SP cells exhibit greater tumor initiating capability than do MP cells and control cells. Data showing tumor initiating ability for SKOV3-control, SP and MP derived from SKOV3 sh-target gene cells are shown. Num; Number, cont; control-MP. (**c**) Limiting dilution assays. (**d**) Tumor growth curves generated from injection of 1 × 10^2^ indicated cells. (**e**–**g**) Overexpression of *VPS45*, *TLE2*, *ZNF498* decreases *in vivo* tumor proliferation. (**e**) Tumor growth curves generated from injection of 1 × 10^5^ A2780 control or ORF-VPS45 cells. (**f**) Tumor growth curves generated from injection of 1 × 10^5^ A2780 control or ORF-TLE2 cells. (**g**) Tumor growth curves generated from injection of 1 × 10^6^ IGROV1 control or ORF-ZNF498 cells. (**h**,**i**) Suppression of six genes enhances resistance to chemotherapeutic agents. *p < 0.05, **p < 0.01, ***p < 0.001, ****p < 0.0001. (**h**) IC50 values for CH1-control, sh-*MSL3*, sh-*ZNF691* and sh-*VPS45* cells treated with Cisplatin (CDDP), Gemcitabine (GEM), doxorubicin (Doxil) and paclitaxel (PTX). (**i**) IC50 values for SKOV3-control, sh-*ITGB3BP*, sh-*TLE2* and sh-*ZNF498* cells treated with CDDP, Gemcitabine (GEM), doxorubicin (Doxil) and paclitaxel (PTX).

To compare *in vivo* tumorigenicity between the newly-generated SP cells and the MP cells derived from the cell line clones with repressed expression of the six genes versus the control cells, we performed a limiting dilution assay. We subcutaneously injected 1 × 10^2^, 1 × 10^3^ and 1 × 10^4^ cells into NOD-SCID mice. The newly generated SP cells derived from three gene–suppressed CH1 cells had significantly higher *in vivo* tumorigenicity as compared to the MP cells and control cells (Fig. 4a,b, SP vs. MP: p = 0.0002, SP vs. control: p < 0.0001). For SKOV3 cells, the newly generated SP cells derived from the three gene-suppressed cells tended to have higher *in vivo* tumorigenicity as compared to the MP cells and control cells (Fig. 4c,d). In addition, the newly generated SP tumors derived from the three SKOV3 cell clones showed a high nuclear/cytoplasmic ratio, relatively small cells. On the other hand, the MP and control SKOV3 tumors showed low N/C ratio, tubular formation, and relatively large cells (Supplementary Fig. S8).

We assessed whether overexpression of these genes altered *in vivo* proliferation. We subcutaneously injected 1 × 10^5^ cells of the A2780-control cells and each of the *MSL3*, *VPS45*, *ITGB3BP*, and *TLE2*-overexpressing A2780 cells into nude mice. We found that overexpression of *VPS45* and *TLE2* significantly decreased *in vivo* proliferation compared to the control cells (Fig. 4e,f; p < 0.05). Although the difference was not statistically significant, overexpression of *ITGB3BP* showed a trend of decreased *in vivo* proliferation (Supplementary Fig. S9a). Overexpression of *MSL3* did not influence *in vivo* proliferation (Supplementary Fig. S9b). We also subcutaneously injected 1 × 10^6^ cells of the IGROV1-control and the *ZNF498*-overexpressing IGROV1 cells into NOD-SCID mice. We found that overexpression of ZNF498 tended to decrease *in vivo* tumorigenicity (Fig. 4g, control, 100%: 6/6 vs *ZNF498*, 33%: 2/6, p = 0.06), and significantly decreased *in vivo* proliferation (Fig. 4g, p < 0.05).

Collectively, general suppression of the identified genes increased *in vivo* tumorigenicity and *in vivo* proliferation, whereas overexpression of these genes decreased *in vivo* proliferation.

### Altered chemosensitivity caused by suppression of the six genes

We examined whether suppression of the six genes changed chemosensitivity. We examined four kinds of chemotherapy: cisplatin (CDDP), paclitaxel (PTX), pegylated liposomal doxorubicin (PLD), and gemcitabine (GEM), which are commonly used for treatment of ovarian cancer. We calculated IC50 values at 72 hours and compared results.

For CH1 cells, suppression of all three genes, *MSL3*, *ZNF691*, and *VPS45* significantly increased the IC50 value of CDDP compared to the control cells (p < 0.0001, Fig. 4h). Suppression of *ZNF691* and *VPS45* significantly increased IC50 values for PTX as compared to the control cells (p < 0.0001, Fig. 4h). Suppression of *VPS45* significantly increased the IC50 values for GEM, (p < 0.05, Fig. 4h), and suppression of *MSL3* significantly increased the IC50 value for PLD (p < 0.05, Fig. 4h).

For SKOV3 cells, suppression of *ITGB3BP* significantly increased the IC50 value of CDDP compared to the control cells (p < 0.001, Fig. 4i). Suppression of all three genes, *ITGB3BP*, *TLE2*, and *ZNF498* significantly increased the IC50 value for PTX compared to the control cells (p < 0.0001, Fig. 4i). Suppression of *TLE2* and *ZNF498* significantly increased the IC50 value of GEM (p < 0.05, p < 0.0001, respectively, Fig. 4i), and suppression of *ZNF498* significantly increased the IC50 value of PLD (p < 0.0001, Fig. 4i).

Therefore, suppression of the six genes caused resistance to multiple chemotherapeutic agents.

### Cancer stem cell phenotype and expression suppression of the six genes

SP cells are known to exhibit cancer stem cell (CSC) phenotypes^8,14,15^. However, although the CSC-like phenotype of the SP-generating cancer cells was suggested through the gene expression analysis (Supplementary Fig. S10), we could not find a significant difference between the SP and MP with regard to ability to undergo asymmetric cell division (Supplementary Fig. S11). Therefore, our experimental data suggests the SP fraction does not exclusively contain the CSC.

### Pathway analysis

**Figure 5:**
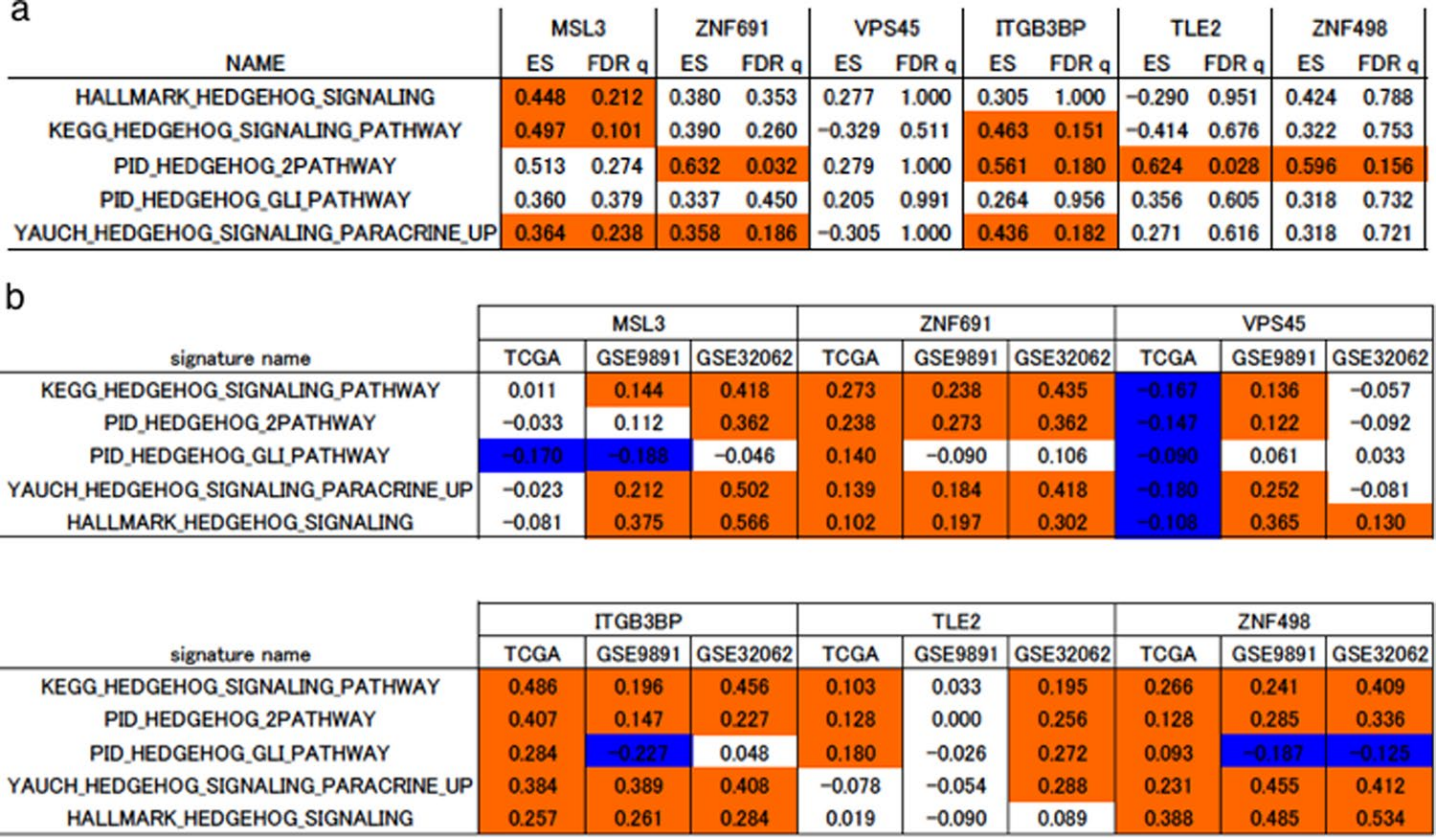
Strong relationships between the hedgehog pathway and six target genes are found. GSEA and ssGSEA analysis showing the relationships between the hedgehog pathway and six target genes. GSE32062 contains 260 patients with ovarian cancer. GSE9891 contain 285 patients with ovarian cancer. (**a**) Results of GSEA analysis. Enrichment score (ES) and FDR q value (FDR q) are shown. Red shading indicates significant enrichment for sh-target genes at an FDR q < 0.25. (**b**) Results of correlation analysis between the scores of the six gene signature and the hedgehog pathway-related signature. Red shading indicates a significant positive correlation (p < 0.05) and blue shading indicates a significant negative correlation (p < 0.05).

We investigated how the six genes caused such a drastic change. We examined the Hedgehog pathway, which is known to be strongly related to side population and sphere formation capacity^16–18^. First, using the RNAseq data, we performed GSEA and found that Hedgehog related pathways were significantly enriched for five of the six genes except for *VPS45* (FDR q < 0.25, Fig. 5a). Next we identified genes whose mRNA expression was significantly altered by suppression of the six genes in the RNAseq data. The identified genes are listed in Supplementary Table S2. Using the ssGSEA method, we calculated scores of the Hedgehog related pathways and the six genes-related pathways in ovarian cancer clinical datasets, and investigated the correlation between them. As a result, we found that there was a significant positive correlation between the six genes-related pathway scores and the Hedgehog related pathway scores in multiple datasets (p < 0.05, Fig. 5b).

**Figure 6:**
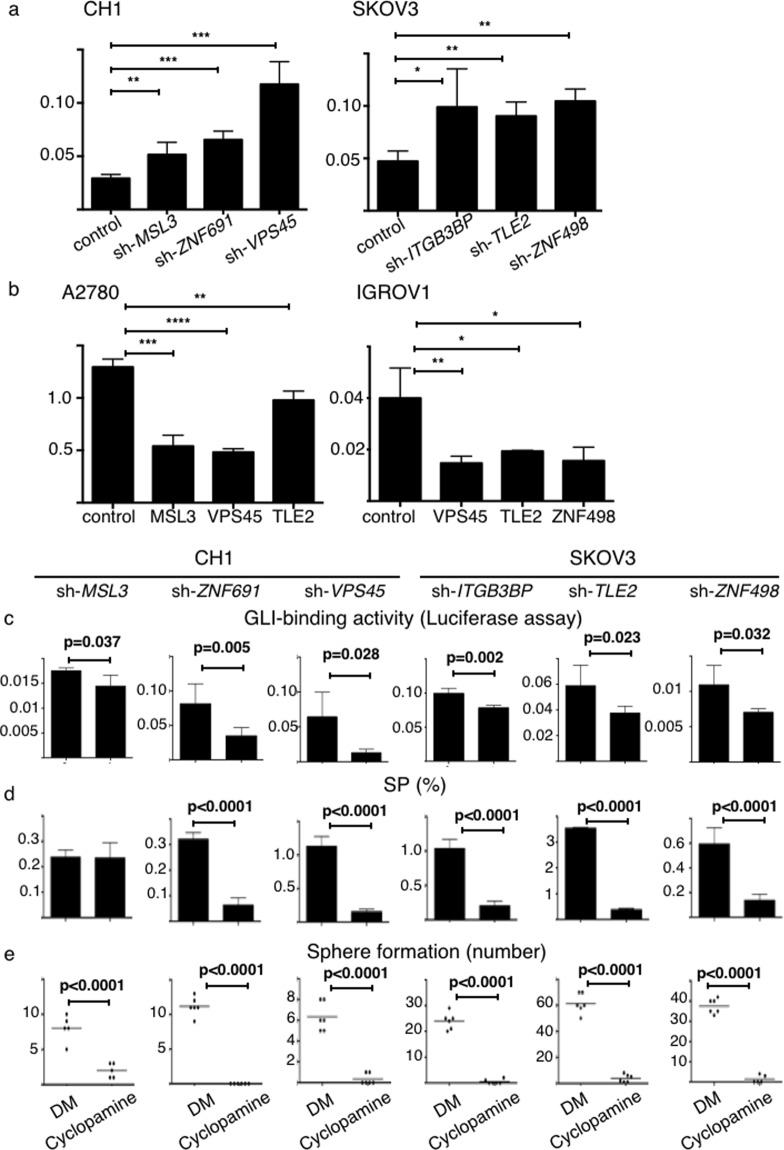
Six genes regulate CSC-like functions via the Hedgehog pathway. DM; DMSO. *p < 0.05, **p < 0.01, ***p < 0.001, ****p < 0.0001. (**a**,**b**) GLI-binding activity measured using a luciferase reporter assay. Relative luciferase activities (y-axis) are shown (n = 4). (**a**) Suppression of the six genes significantly increases GLI-binding activity compared to control cells. (**b**) Overexpression of sMDL3, VPS45, TLE2 and ZNF498 significantly decrease GLI-binding activity (y-axis) compared to control cells. (**c**) Cyclopamine inhibits GLI-binding activity, measured using a luciferase reporter assay. (**d**) Cyclopamine decreases the SP, with the exception of SP cells derived from suppression of *MSL3*. (**e**) Cyclopamine decreases sphere forming capacity of SP cells derived from suppression of six genes.

Then, we found suppression of the six genes significantly increased the GLI-binding activity compared to the control group (p < 0.05, Fig. 6a). Conversely, overexpression of *MSL3*, *VPS45*, *TLE2*, and *ZNF498* significantly decreased the GLI-binding activity compared to the control cells (p < 0.05, Fig. 6b). GLI-binding activity that was increased by suppression of the six genes was significantly diminished by treatment with cyclopamine, an inhibitor of the Hedgehog pathway. Moreover, cyclopamine significantly decreased the proportion of the newly generated SP cells and sphere forming capacity (p < 0.05, Fig. 6c–e).

In addition to the Hedgehog pathway, we analyzed different pathways, the transforming growth factor *β* (TGF-*β*) pathway^19,20^, the Wnt pathway^21,22^ and the Notch pathway^23,24^, which have been reported to relate to the SP, malignancy or CSC phenotype in ovarian cancer. However, analyses of these three pathways did not result in consistent data as observed for the Hedgehog pathway analysis (see Supplementary Material and Method, Supplementary Table S3a,b,c, Supplementary Figs S12, S13).

## Discussion

In this study, we identified six novel genes, *MSL3*, *ZNF691*, *VPS45*, *ITGB3BP*, *TLE2*, and *ZNF498* whose expression altered the proportion of the SP cells of ovarian cancer cells through a functional genomics screen. To our knowledge, this is the first report that directly identified novel factors contributing to the proportion of SP cells in cancer cells by way of a functional genomics screen.

So far, little is known about the detail of the six genes that we found, *MSL3*, *ZNF691*, *VPS45*, *ITGB3BP*, *TLE2*, and *ZNF498*. There are no published reports about *ZNF691* and *ZNF498*. *MSL3* is known to be related to histone modification^25^. This suggests that *MSL3* could have an influence on the expression of various genes. *VPS45* is known to relate to cellular trafficking pathways and autophagy^26^. Mutations in *VPS45* have been reported in a congenital neutrophil defect syndrome^27^. *VPS45* is also reported as a tumor suppressor^28^. *ITGB3BP* is reported to function as a tumor suppressor in breast cancer^29^. *TLE2* is a co-repressor of the Wnt-*β* catenin pathway^30^ and also known to regulate ventral telencephalon formation^31^. There are no published reports regarding a role for *TLE2* in cancer.

SP cells are identified on the basis of efficient efflux of Hoechst 33342 dye. SP cells are also known to have some malignant phenotypes, such as the capacity for sphere formation, single cell clonogenicity, *in vivo* tumorigenicity, and chemoresistance^8–10,14,15^. In this study, we found that sphere forming capacity, single cell clonogenicity, and *in vivo* tumorigenicity of newly generated SP cells were significantly higher than those of the MP cells and control cells (Figs 3a–h, 4a–d). Moreover, expression of the six genes studies had a major influence on chemoresistance and *in vivo* tumor proliferation for the entire tumor cell population (Fig. 4e–i). Additionally, we found changes in cell morphology following suppression or overexpression of the six genes (Supplementary Figs S7, S8), a phenotype frequently observed in the process of acquisition of chemoresistance, tumor metastasis, and inactivation of tumor suppressors^32–35^. These results are relevant here because the SP-generating shRNAs identified through the SP-specific genomics screen also enhanced malignant phenotypes. These results strongly support the intimate relationship between the SP fraction and malignant phenotypes of cancer cells.

In clinical data analysis, we found that suppressed expression of the six genes was related to poor prognosis, dissemination, and chemoresistance of ovarian cancer (Fig. 2, Supplementary Fig. S6a–i). Some have reported that the proportion of the SP cells relative to the total number of cancer cells is clinically important. The proportion of SP cells varies across clinical samples^10,36–38^, and the proportion of SP cells in metastatic and recurrent tumors is reported as higher than that in the primary tumor^10^. Moreover, the more malignant the tumor phenotype, the higher the proportion of SP cells found^10,38–41^. The proportion of SP cells in and of itself, or genes that are overexpressed in the SP cells, are also reported as independent poor prognostic factors^11,42,43^. Because repression of the six individual genes increased the SP as we have demonstrated, and because ovarian cancer is characterized by marked chromosomal instability^44^, ovarian cancer may easily enhance the malignant phenotype during progression or relapse via a genetic change that increases the fraction of SP cells.

Many have reported that CSCs are enriched in the SP cells^8,14,15^. Sphere formation, single cell clonogenicity, and *in vivo* tumorigenicity are hallmarks of CSCs. Asymmetric division is also an important characteristic of CSCs. That is, CSCs can generate both CSCs and differentiated cells, whereas differentiated cells can generate only differentiated cells. In this study, suppression of the six genes tended to cause activation of CSC-related signatures (Supplementary Fig. 10a–f). However, asymmetric cell division was not observed; i.e., the MP cells generated the SP cells (Supplementary Fig. S11). Consistent with our data, the MP cells are reported to generate SP cells in many studies, although the ratios vary^8,9,36,40,45,46^. Therefore, we think the relationship between the SP and MP is not identical to that between CSCs and non-CSCs. In addition, knockdown efficacy may vary between each cell, which might explain why not all cells were changed to SP cells by suppression of each of six genes. To date, there is no available antibody for flowcytometry about six genes. Thus, it was difficult to evaluate the expression of six genes in each cell. Further study is required to elucidate this point.

Several reports have shown that activation of the hedgehog pathway has a major influence on the proportion of SP cells and their related malignant phenotype^16–18^. For ovarian cancer, activation of the Hedgehog pathway causes chemoresistance and cell proliferation, and results in poor prognosis^47,48^. Our data support a strong association between the SP and the Hedgehog pathway in ovarian cancer cells because the six genes identified through the SP-specific genomic screen are involved in the Hedgehog pathway (Fig. 6). Although we could not find the enrichment of Hedgehog pathway by suppression of VPS45, VPS45 is shown to relate to the Hedgehog pathway based on the results of GLI-binding activity and Hedgehog pathway inhibitor experiments. VPS45 could influence downstream factors of Hedgehog pathway. In addition, there might be other mechanisms that associate with each of the six genes and generation of SP cells. In this research, we tried identifying the most common change among the six genes that relate to generation of SP cells, and found that our six genes are most commonly associated with the Hedgehog pathway. Focusing on each of the six factors may lead to the discovery of some other mechanism(s) in each of the six genes, which might reveal some other mechanism(s) related to acquired resistance of ovarian cancer.

In conclusion, through a functional genomics screen using an shRNA library we identified six novel genes; *MSL3*, *ZNF691*, *VPS45*, *ITGB3BP*, *TLE2* and *ZNF498*, whose downregulation individually increased the proportion of SP cells and the malignant phenotypes of ovarian cancer. Suppression of these six genes activated the Hedgehog pathway. These results further our knowledge regarding the acquisition of treatment resistance that frequently occurs in ovarian cancer, and suggest a novel therapeutic approach to treat refractory ovarian cancer.

## Methods

### Cell lines and reagents

We maintained human ovarian cancer cell lines, CH1, SKOV3, A2780, and IGROV1 in RPMI 1640 (Nacalai Tesque, Kyoto, Japan) as previously reported^13^. 293FT cells were purchased from Thermo Fisher Scientific (Waltham, USA), and cultured as previously reported^13^.

Cyclopamine (LKB labs, St Paul, USA) was used to inhibit the Hedgehog pathway.

### Side population (SP) analysis

Cells were detached, centrifuged and resuspended in tissue culture medium containing 10% serum at a concentration of 1 × 10^6^ cells/mL. The cells were labeled with 5.0 µg/mL of Hoechst 33342 dye (Sigma-Aldrich, St. Louis, USA) at 37 °C for 90 min either alone or in combination with ABC efflux pump inhibitor Reserpine (Sigma-Aldrich) at a concentration of 15 µM or 30 µM. 7-amino-actinomycin D (Becton, Dickinson and Company; BD, Franklin Lakes, USA) was added to the cells to a final concentration of 2 µg/mL prior to FACS analysis. The SP analysis was done using a BD FACS AriaII system (BD Biosciences). The Hoechst dye was excited with UV laser and its fluorescence was measured with both 675LP (Hoechst Red) and 440/40 filters (Hoechst Blue). Cell sorting was performed according to the manufacturer’s protocol.

### First shRNA library screen

We examined several human ovarian cancer cell lines and found that CH1 and SKOV3 harbor relatively few SP cells, while A2780 and IGROV1 harbor relatively more SP cells. We used CH1 and SKOV3 cells for the screen. We transfected The DECIPHER RNAi library Module (Cellecta, Mountain View, USA), an shRNA library comprising ~80 000 plasmids targeting ~15 000 genes inserted into a pLKO1 lentiviral vector, into cells at a multiplicity of infection (MOI) of 0.3 as previously reported^13^. Following 72 hours of selection with puromycin (Thermo Fisher Scientific), 3.0 × 10^7^ stably transduced CH1 cells and 1.5 × 10^7^ SKOV3 cells were incubated with Hoechst dye and analyzed to identify the SP. Single SP cells were sorted directly into 96-well plates with culture medium using FACS AriaII system (BD), and incubated for expansion. We extracted DNA using the DNeasy Blood and Tissue Kit (Qiagen, Venlo, Nederland) in preparation for subcloning.

### Subcloning and reconstruction of shRNA plasmids, followed by the second screen

Subcloning and reconstruction of the shRNA plasmids were performed as previously reporte^13^. Briefly, we PCR amplified the shRNA target sequences using the primers listed in Supplementary Table S4. The amplified PCR products were subcloned into the original pLKO1 lentiviral vector using the InFusion HD cloning Kit (Takara Bio, Otsu, Japan) according to the manufacturer’s protocol, thus reconstructing the pLKO1 shRNA lentiviral plasmids that were identical to those included in the original shRNA library. We subcloned at least five clones per PCR amplicon. To identify the cloned shRNAs, we sequenced the anti-sense strands by Sanger sequencing (3130xl Genetic Analyzer) (Thermo Fisher Scientific) using primers listed in Supplementary Table S4, which were purchased from Greiner. In the second screen we transfected each reconstructed shRNA plasmid one by one into CH1 or SKOV3 cells and performed SP analysis.

### RNA extraction and real time quantitative PCR

Total RNA extraction, reverse transcription and real time quantitative reverse transcriptase (RT)-PCR reactions were performed as previously reported^13^. Primers were listed in Supplementary Table 4.

### Stable knockdown and overexpression of target genes

TRC lentiviral target gene-specific shRNAs (GE Healthcare Life Science, Backinghamshire, UK), which are listed in Supplementary Table S5, were used to suppress the mRNA expression of target genes as recommended by the manufacturer. We also transfected the non-silencing control TRC lentiviral shRNA plasmid into cells in the same manner.

To establish stable cells that overexpress some target genes, we generated the lentiviral open reading frame (ORF) plasmids by editing the LentiORF plasmids (GE Healthcare Life Science). Details are described in Supplementary Material and Method. We also transfected the control Precision LentiORF in the same manner.

### Gene expression microarray datasets

Microarray datasets were obtained from the Gene Expression Omnibus website (http://www.ncbi.nlm.nih.gov/geo). The TCGA dataset includes 557 HGSOC patients. Level three microarray data and patient information were downloaded from the Cancer Browser (https://genome-cancer.ucsc.edu/). To analyze the relationship between expression of the six genes and the hedgehog pathway, we used GSE9891 and GSE32062.

### RNA sequencing

Details of Library preparation, RNA sequencing and transcriptome analysis are described in Supplemental Material and Method.

### GSEA and ssGSEA

We downloaded the gene sets listed in Supplementary Table S6 from the Molecular Signatures Database version 5.0 (http://www.broadinstitute.org/gsea/msigdb) and conducted gene set enrichment analysis (GSEA version 2.2.4: Broad Institute, Cambridge, MA; http://software.broadinstitute.org/gsea) using the microarray data sets and gene expression data from our cell lines as previously described.

Single-sample GSEA (ssGSEA) was performed by gene level, as described (GenePattern version 5.0; Broad Institute; http://www.broadinstitute.org/cancer/software/genepattern).

### Sphere forming assays

For assessment of sphere forming capacity, cells were plated as single cells on ultralow attachment 24-well plates (Corning, NY, USA) (2,000 cells/well). Cells were grown in conditioned medium, which consisted of serum-free DMEM/F-12 medium (Thermo Fisher Scientific) supplemented with 20 ng/ml EGF (Merk Millipore, Billerica, USA), 10 ng/ml basic fibroblast growth factor (bFGF; PeproTech, Rocky Hill, USA) and B-27(X100) supplement (Thermo Fisher Scientific.). Cyclopamine (LKT Laboratories, Inc.) was used at 20 µM.

### Single cell clonogenicity assays

For assessment of single cell clonogenicity, cells were single-cell-sorted into 96-well plates containing 150 µl culture medium. Plates were incubated for two weeks and the number of wells harboring viable clones were counted.

### Xenografts

Cells were admixed with 75 µL Matrigel (BD Biosciences) and the cell mixture was injected into both flanks of six-week-old NOD/SCID mice or nude mice. NOD/SCID mice (NOD.CB17-Prkdc^scid^/J) and nude mice (CAnN.Cg-Foxn1^nu^/CrlCrlj) were purchased from Charles River Laboratories Japan, Inc. (Yokohama, Japan). Tumors larger than 100 mm^3^ were counted. Tumor volume was measured two times a week using the following formula: V = 1/2(L × W2), where L equals length, and W equal width. Sample sizes were chosen to assure reproducibility of the experiments in accordance with the replacement, reduction, and refinement principles of animal ethics regulation. All animal studies were approved by the Kyoto University Animal Research Committee.

### Chemotherapy sensitivity assays

Cells were plated into 96-well plates at 2,000~4,000 cells per well. Twenty-four hours later, the culture medium was replaced with fresh medium containing various drug concentrations, including cisplatin (Nippon Kayaku, Kyoto, Japan) paclitaxel (Bristol-Myers Squibb, New York, USA), gemcitabine (Eli Lilly Japan, Kobe, Japan) and liposomal doxorubicin (Janssen Pharmaceutical K.K., Tokyo, Japan) for 72 hours. The percentage of viable cells was then examined using the WST-1 assay kit following the manufacturer’s instructions (Premix WAT-1®, Takara Bio). All cytotoxicity data shown are the means of at least three independent experiments.

### Luciferase reporter assay

The pGL3/(3′Gli-BS)n-Luc vector was kindly provided by Dr. Katagiri and Dr. Harada^49^.

Luciferase reporter assays were performed by transfecting the reporter construct into target cells. The pRL-CMV vector was co-transfected in each experiment as an internal control for transfection efficiency. At 48 hours post-transfection, luciferase activity was measured using the Dual-Luciferase Reporter Assay System (Promega, Madison, USA) according to the manufacturer’s protocols.

For analysis of cyclopamine inhibition of GLI-binding activity, the reporter plasmid and the pRL-CMV vector were co-transfected followed 12 hours later by addition of cyclopamine (20 µM) or DMSO. Following 48 hours incubation with cyclopamine or DMSO, luciferase activity was measured using the Dual-Luciferase Reporter Assay System (Promega).

### Statistical analysis

Results are shown as averages ± standard deviation (SD). Group comparisons were performed using unpaired t-tests. Comparisons between paired samples were performed using paired t-tests. Prognostic analyses were performed using the Log-rank test. All statistical analyses were performed using GraphPad Prism 6.0 and R software. Probability values below 0.05 and FDR q values below 0.25 were considered significant.

### Study approval

All animal studies were approved by Kyoto University Animal Research Committee and performed with the approval of the Institutional Animal Care and Use Committee.

## Supplementary information

Supplementary Information

## Data Availability

All data generated and analyzed during this study are included in this article ant its additional files except for RNAseq data. The RNAseq datas generated and analyzed during the current study are available in the GEO repository and GSE number is 117480.
