# Supplementary material for: Acquisition of a side population fraction augments malignant phenotype in ovarian cancer
Source: Sci Rep. 2019 Oct 2;9:14215. doi: 10.1038/s41598-019-50794-w (PMC6775117; doi:10.1038/s41598-019-50794-w)
Supplement: Supplementary file 1 — Supplementary Information [file 41598_2019_50794_MOESM1_ESM.pdf]

Title

Acquisition of a side population fraction augments malignant phenotype in ovarian cancer.

Authors

Koji Yamanoi[1], Tsukasa Baba[1], Kaoru Abiko[1], Junzo Hamanishi[1], Ken Yamaguchi[1,2] , Ryusuke Murakami[1], Mana Taki[1], Yuko Hosoe[1], Susan K Murphy[3], Ikuo Konishi[1,2], Masaki Mandai[1], Noriomi Matsumura[1,4] †

Affiliations

1) Department of Gynecology and Obstetrics, Kyoto University Graduate School of Medicine.
2) Department of Obstetrics and Gynecology, National Hospital Organization Kyoto Medical Center.
3) Division of Gynecologic Oncology, Department of Obstetrics and Gynecology, Duke University Medical Center.
4) Department of Obstetrics and Gynecology, Faculty of Medicine, Kindai University.

† Corresponding Author; Noriomi Matsumura,

Supplementary Figure

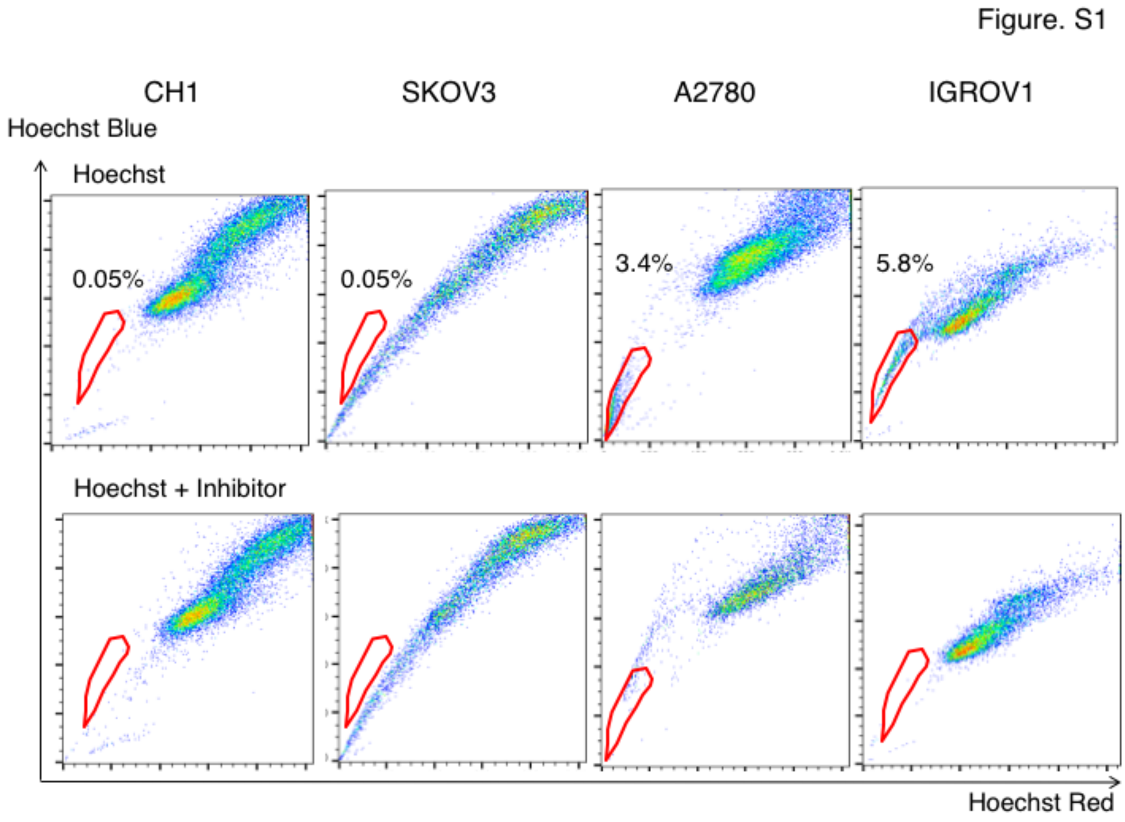

Supplementary Fig. S1. SP fraction of several ovarian cancer cell lines.

Representative data showing the percentage of the SP fraction of CH1, SKOV3, A2780 and IGROV1. SP fraction is marked with red line.

Figure. S2

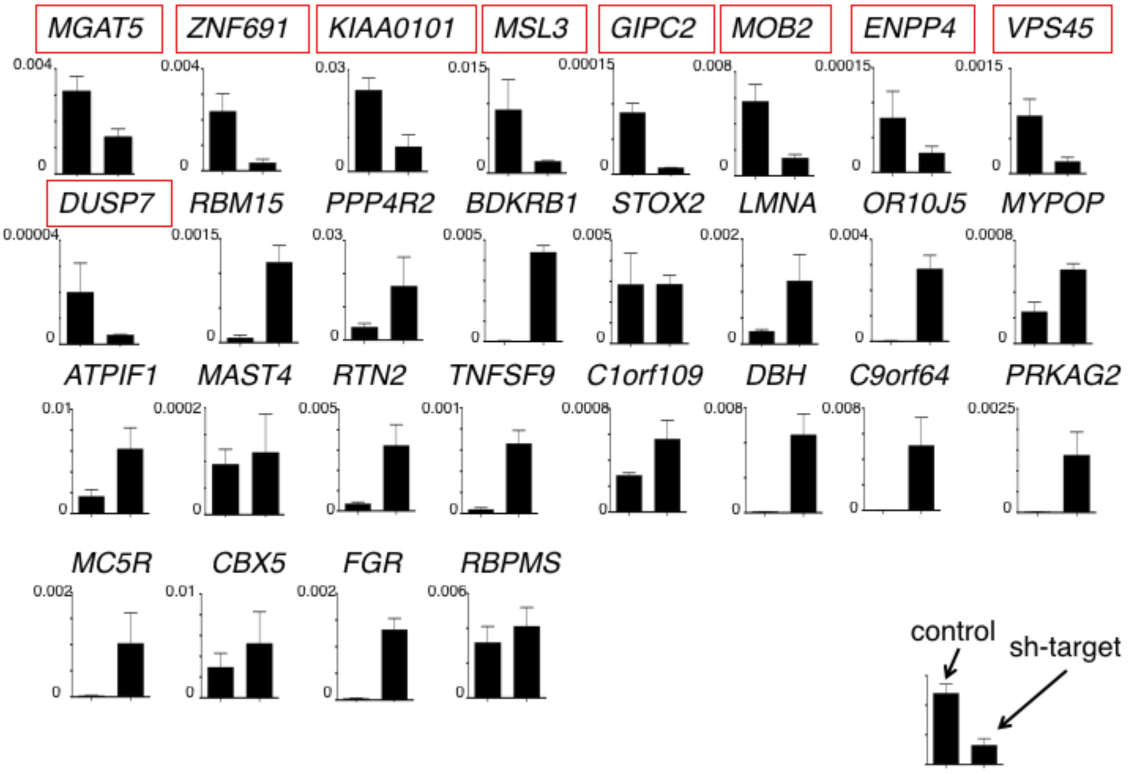

Supplementary Fig. S2. Confirmation of mRNA expression.

RT-PCR showing knockdown of shRNA target genes for the shRNAs that markedly increased the SP fraction in CH1 cells. Red outline indicates that the target gene expression is significantly suppressed.

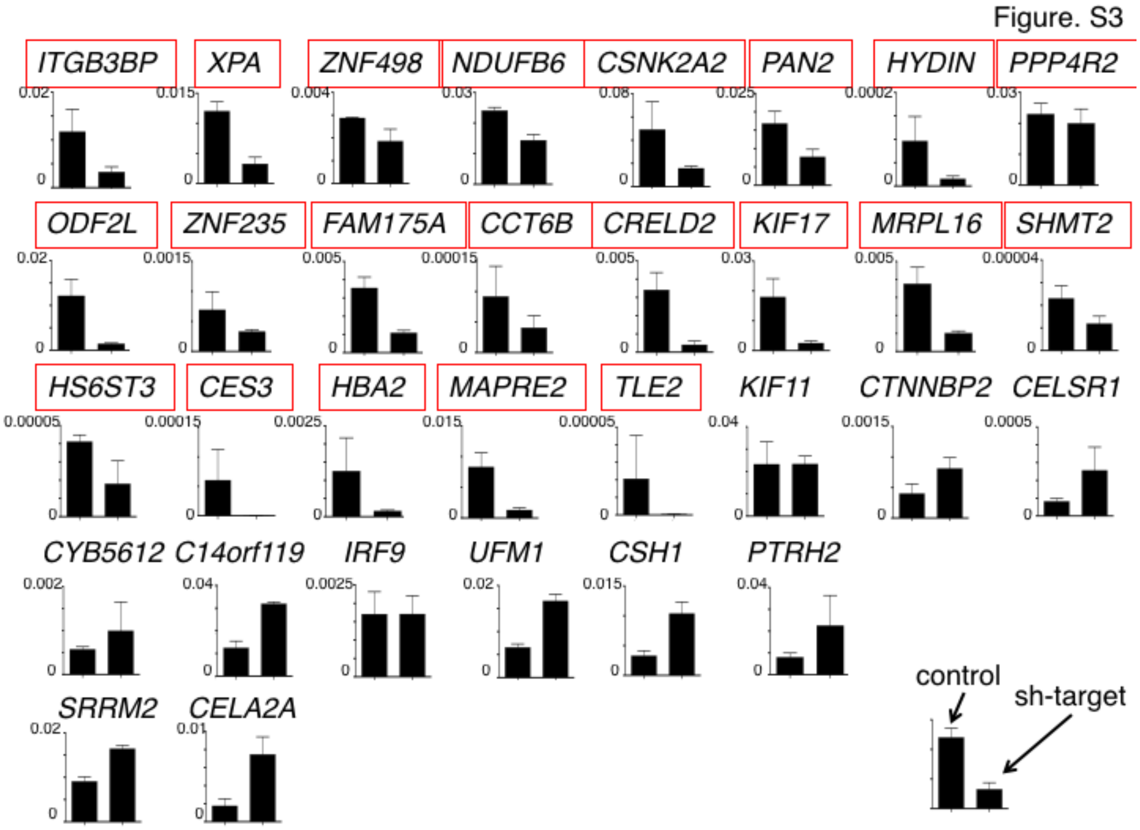

Supplementary Fig. 3. Confirmation of mRNA expression.

RT-PCR showing knockdown of shRNA target genes for the shRNAs that markedly increased the SP fraction in SKOV3 cells. Red outline indicates that target gene expression is significantly suppressed.

Figure. S4

a

Mean number: SP(%)

	MSL3	ZNF691	VPS45	MOB2	DUSP7	MAST4	MGAT5	GIPC2	KIAA0101	ENPP4
sh1	1.05	0.015	0.22	0.2	0.18	1.5	0.01	0.01	0.046	0.22
sh2	0.79	0.01		0.03	0.22	1	0.01	0	0.11	0.01
sh3	0.46	0.96		0.1	0.57	1.7	0.01	0.11	0.044	

b

	ITGB3BP	TLE2	ZNF498	PAN2	CSNK2A2	NDUFB6	ZNF235	XPA	ODF2L	FAM175A
sh1	1.02	1.11	0.59	0.024	0.88	0.042	1.9	0.39	0.023	0.18
sh2	0.28	0.72	0.27	0.27	0.21	0.085	0.61	0.011	0.13	0.16
sh3	0.35	0.61	0.59	0.056	0.49	0.11	0.046	0.12	0.043	0.53
sh4	1.38	0.59	0.37	0.053	0.033		0.15			
sh5			0.7	0.034	0.55					

	HYDIN	KIF17	CCT6B	CRELD2	HBA2	SHMT2	MRPL16	CES3	HS6ST3	MAPRE2
sh1	0.11	0.11	0.28	0.069	0.024	0.039	0.1	0.01	0.067	0.013
sh2	0.42	0.3	0.18	0.072	0.63	–	0.061	0.21	0.17	0.018
sh3	0.68		0.29	0.16	–	0.041	0.037	0.036	0.017	0.0001
sh4				0.14						

Significant increase of SP   No change of SP

Suppression of mRNA expression of target genes

Supplementary Fig. 4. Excluding off target effects and identifying six genes.

We excluded off target effects and identified six genes whose suppression markedly increased the SP fraction. Red shading indicates a significant increase ($p<0.05$) of SP cells as compared to the controls. The purple outline indicates significant suppression ($p<0.05$) of mRNA expression as detected by RT-PCR.

a) Analysis using CH1 cells.

b) Analysis using SKOV3 cells.

Figure. S5

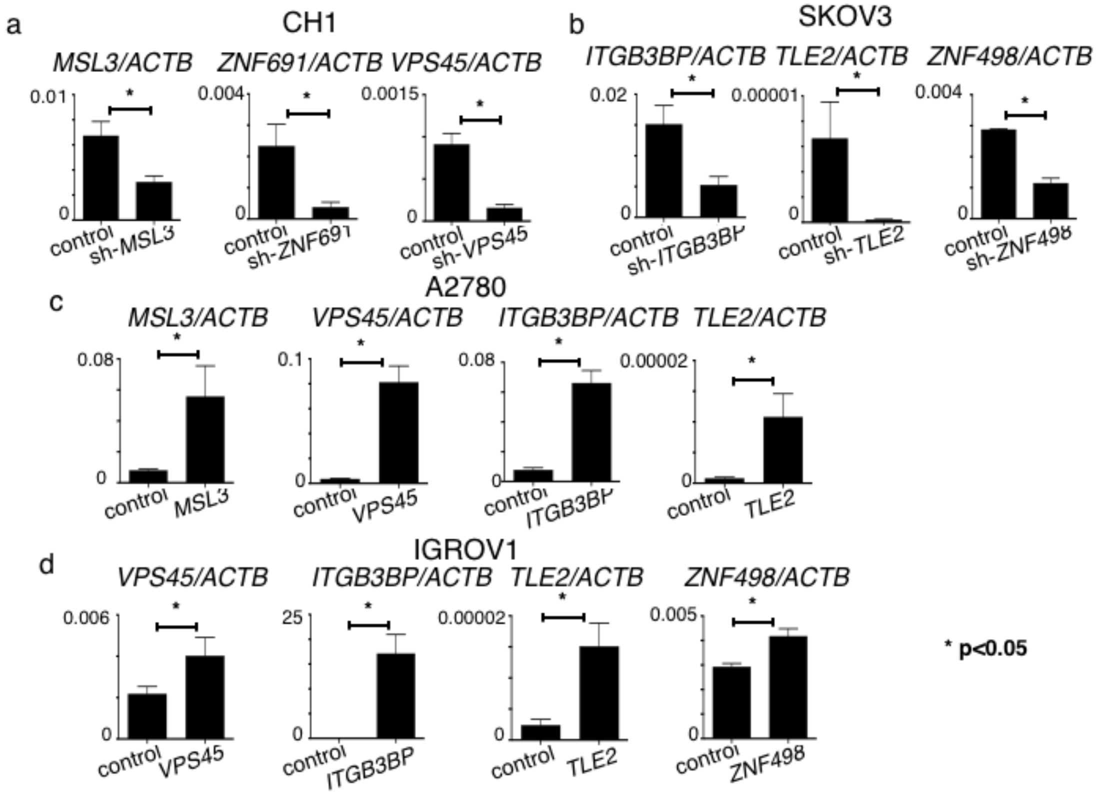

Supplementary Fig. 5. Establishment of cell lines with altered expression of the six target genes.

Target gene mRNA suppression or overexpression was measured by RT-PCR.

a) Establishment of CH1 clones whose mRNA expression for *MSL3*, *ZNF691* or *VPS45* is suppressed by shRNAs.

b) Establishment of SKOV3 clones whose mRNA expression for *ITGB3BP*, *TLE2* or *ZNF498* is suppressed by shRNAs.

c) Establishment of A2780 clones with elevated mRNA expression for *MSL3*, *VPS45*, *ITGB3BP* or *TLE2*.

d) Establishment of IGROV1 clones with elevated mRNA expression for *VPS45*, *ITGB3BP*, *TLE2* or *ZNF498.*

*; $p<0.05$.

Figure S6

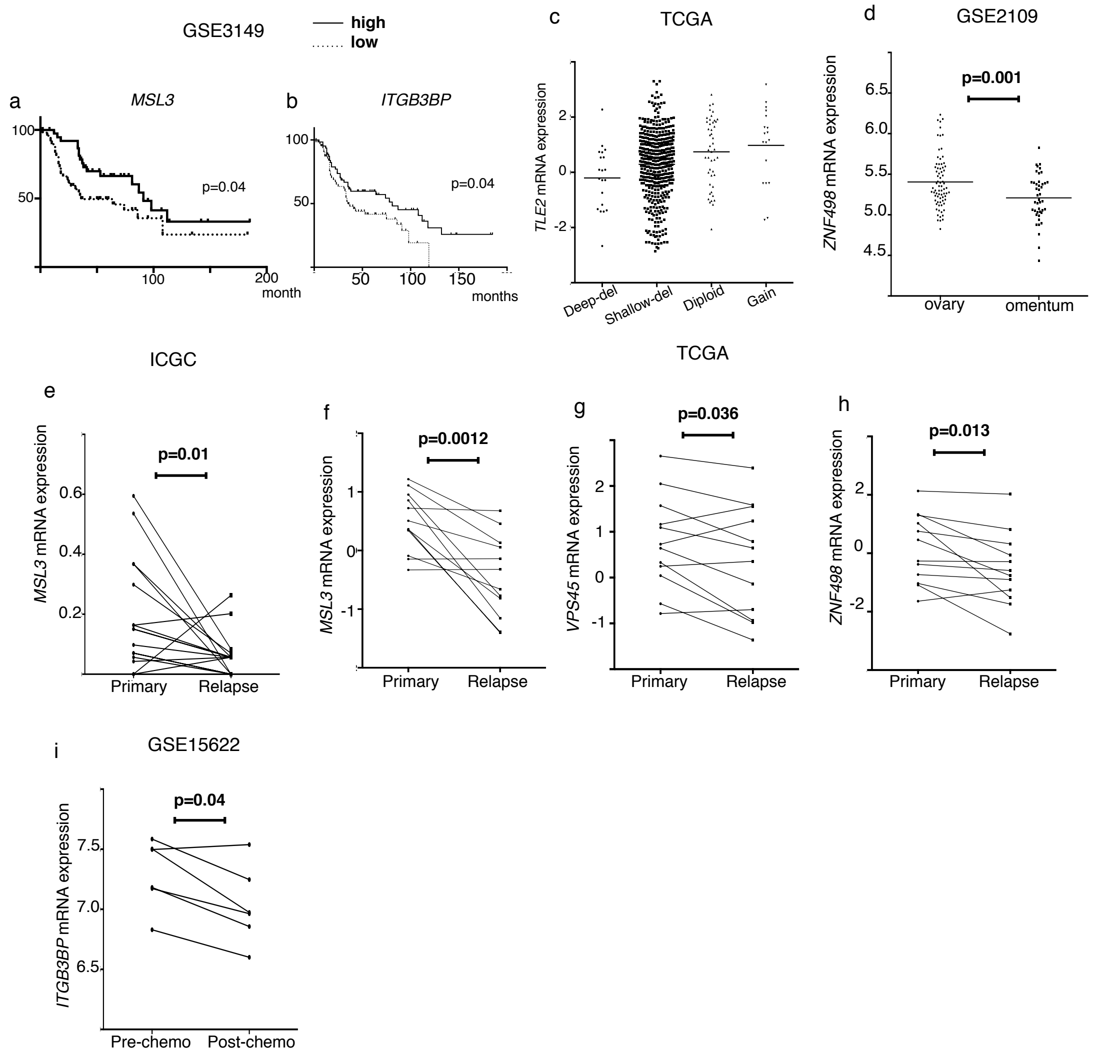

Supplementary Fig. 6. Analyses of the clinical data of the six genes.

a) Overall survival of ovarian cancer patients in data set GSE3149. Differences in survival based on *MSL3* mRNA expression are shown. The low expression group consisted of the 50 cases with the lowest level of expression while the high expression group consisted of the 50 cases with the highest level of expression.

b) Overall survival of ovarian cancer patients in data set GSE3149. Differences in survival

based on *ITGB3BP* mRNA expression are shown. Samples were stratified into two groups based on the median of mRNA expression.

c) Copy number alterations of *TLE2* in TCGA samples. del; deletion.

d) Comparison of *ZNF498* mRNA expression between ovarian cancer tissues from the primary site (n=75) and that from disseminated sites (n=45) using gene expression microarray dataset GSE2109.

e) Change in *MSL3* mRNA expression levels between pre-chemotherapy and post-chemotherapy paired samples (n=17) from dataset ICGC.

f~h) Analysis using primary and relapsed paired samples (n=12) from dataset of TCGA.

f) Change in *MSL3* mRNA expression.

g) Change in *VPS45* mRNA expression.

h) Change in *ZNF498* mRNA expression.

i) Change in *ITGB3BP* mRNA expression levels between pre-paclitaxel treatment and post-paclitaxel treatment paired samples (n=6) from dataset GSE15622.

The details of each dataset are described in Supplementary Material and Method.

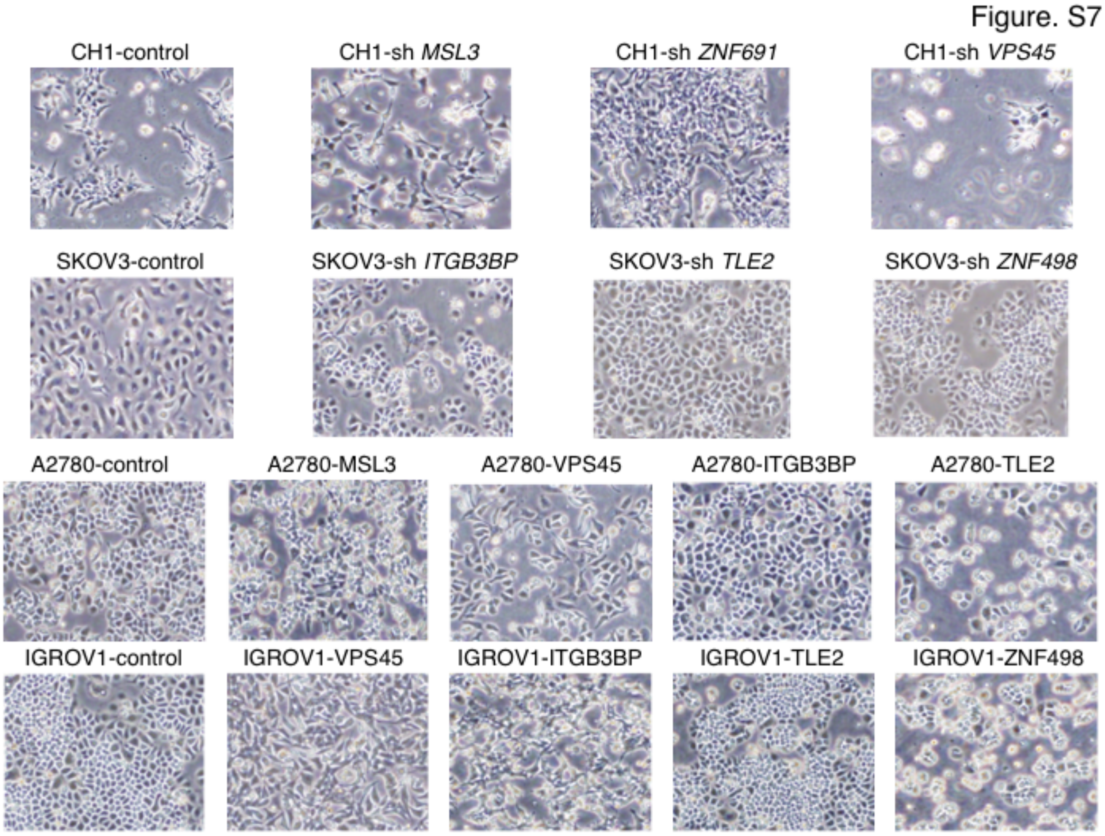

Supplementary Fig. 7: Alteration of mRNA expression of the six target genes changes cell morphology in vitro.

For typically spindle-shaped SKOV3, suppression of *ITGB3BP, TLE2,* and *ZNF498* led to a cobblestone appearance (Supplementary Fig. S8). For cobblestone shaped A2780 cells, overexpression of *MSL3* and *VPS45* changed the morphology to spindle shaped. For cobblestone shaped IGROV1, overexpression of *VPS45* and *ITGB3BP* changed the cell morphology to spindle.

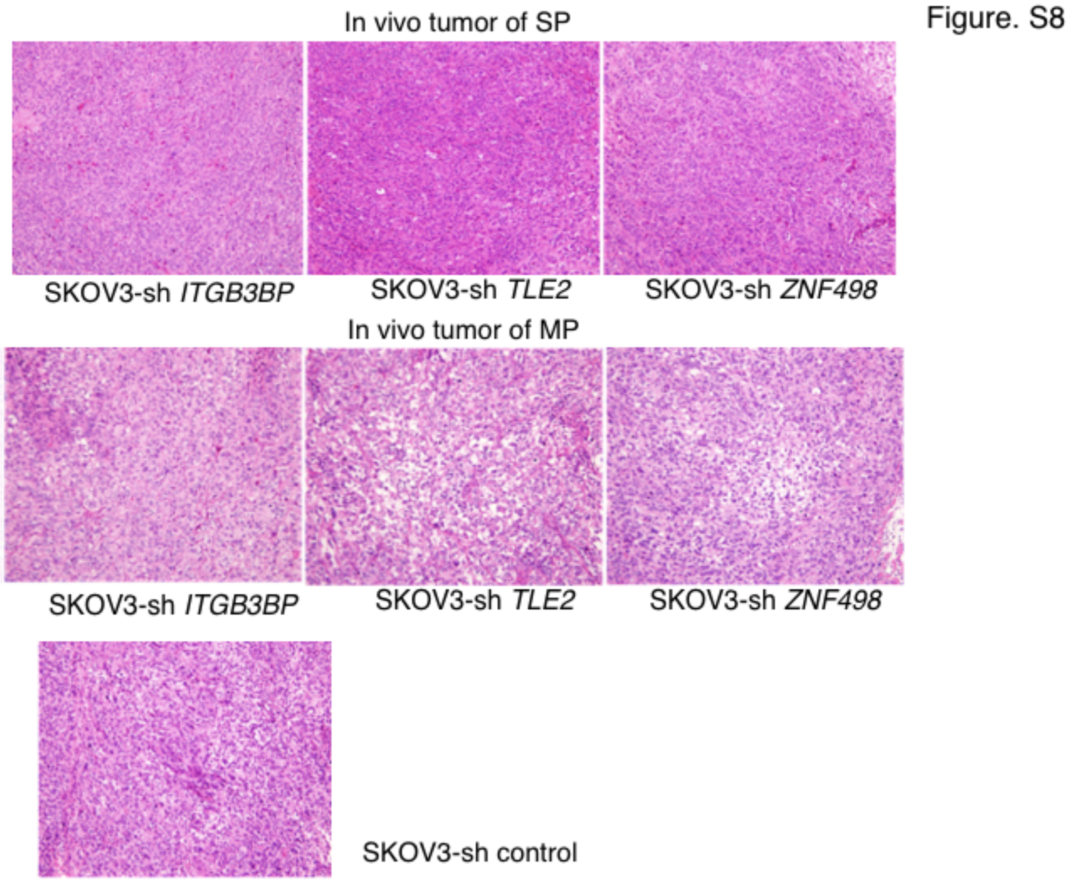

Supplementary Fig. 8. Sections from in vivo tumors formed by newly generated SP cells derived from suppression of ITGB3BP, TLE2 and ZNF498 exhibit different morphology from those of MP or control cells.

Figure. S9

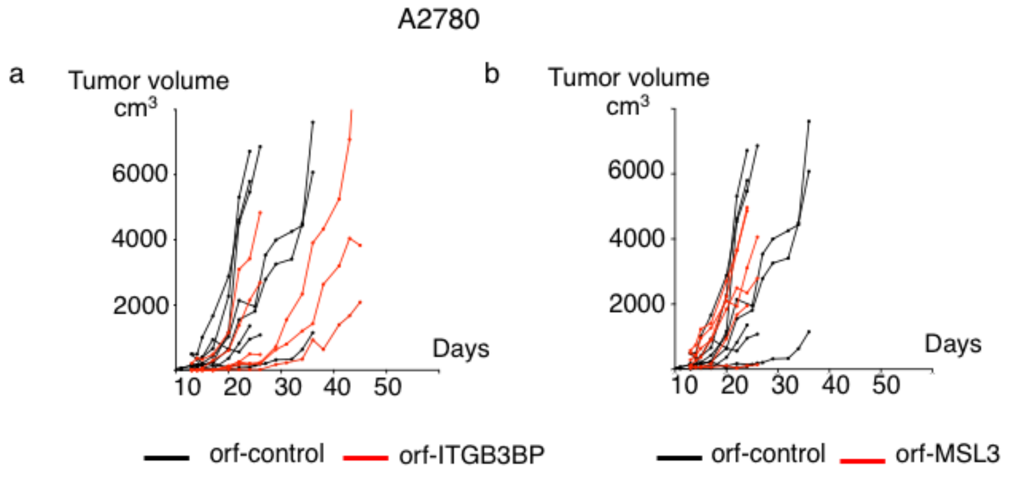

Supplementary Fig. 9. Overexpression of six genes regulates in vivo tumor proliferation.

a) Tumor growth curves from injection of $1x10^5$ A2780 control cells and A2780 ORF-ITGB3BP. Overexpression of ITGB3BP tended to decrease in vivo proliferation ability.

b) Tumor growth curves from injection of $1x10^5$ A2780 control cells and A2780 ORF-MSL3.

X-axis, days post-injection; Y axis, tumor volume.

Figure. S10

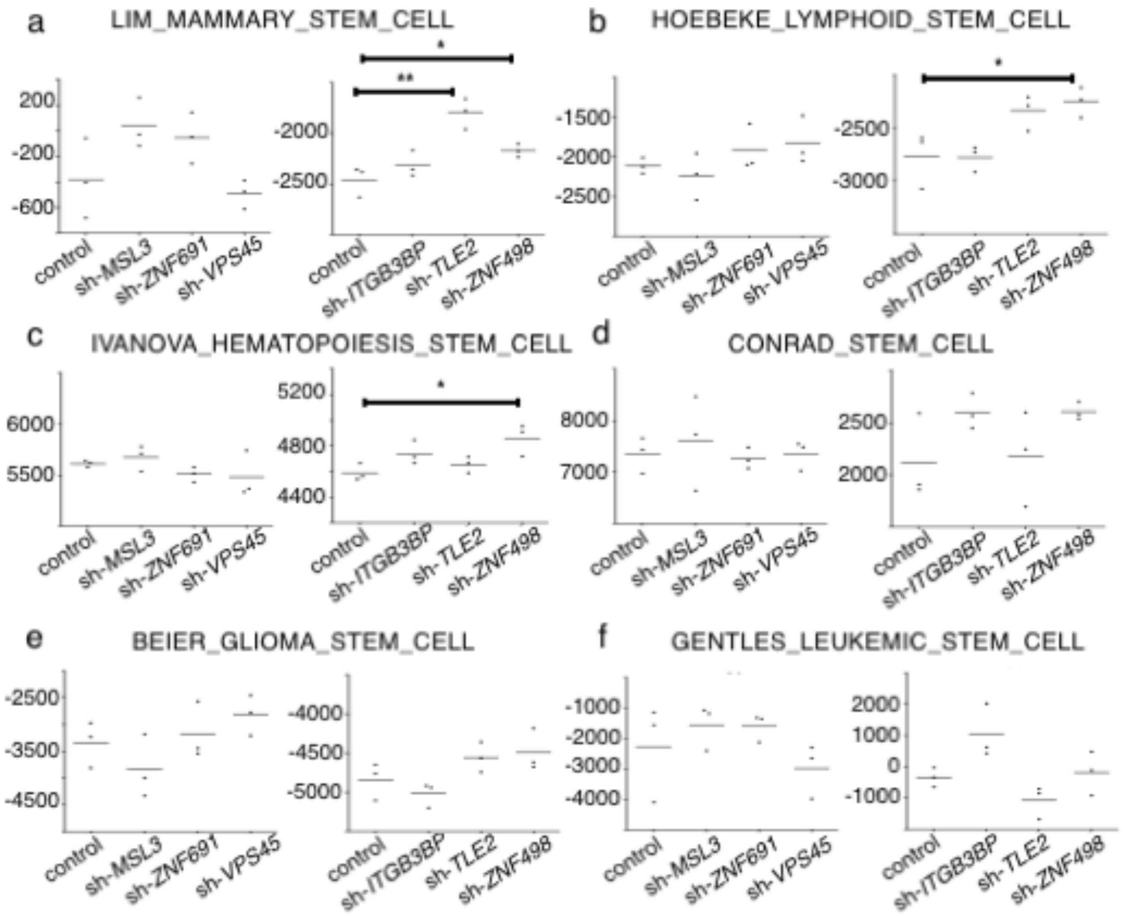

Supplementary Fig. 10. Suppression of six target genes can enhance the CSC phenotype.

ssGSEA analyses are shown. Comparison of ssGSEA scores for CH1-shcontrol, sh-MSL3, sh-ZNF691 and sh-VPS45 samples, and for SKOV3-shcontrol, sh-ITGB3BP, sh-TLE2 and sh-ZNF498 samples (n=3, each). Signatures related to CSC phenotype were analyzed. **p<0.01 *p<0.05

(a) Signature of LIM_MAMMARY_STEM_CELL

(b) Signature of HOEBEKE_LYMPHOID_STEM_CELL

(c) Signature of IVANOVA_HEMATOPOIESIS_STEM_CELL

(d) Signature of CONRAD_STEM_CELL

(e) Signature of BEIER_GLIOMA_STEM_CELL

(f) Signature of GENTLES_LEUKEMIC_STEM_CELL

We performed a transcriptome analysis and investigated potential CSC-like functions. We extracted RNA from samples of the CH1-sh-control, CH1-sh-MSL3, CH1-sh-ZNF691, CH1-sh-VPS45, SKOV3-sh-control, SKOV3-sh-ITGB3BP, SKOV3-sh-TLE2, and SKOV3-sh-ZNF498 followed by RNA sequencing (n=3 each). Using this data, we calculated the scores of stem cell-related gene signatures by ssGSEA. We found that the gene sets, CONRAD_STEM_CELL, BEIER_GLIOMA_STEM_CELL, GENTLES_LEUKEMIC_STEM_CELL, LIM_MAMMARY_STEM_CELL, HOEBELE_LYMPHOID_STEM_CELL, and IVANOVA_HEMATOPOIESIS_STEM_CELL, tended to increase with suppression of the six genes (a-f). In particular, the score of the LIM_MAMMARY_STEM_CELL signature was significantly higher in the sh-TLE2 group compared to the control group (a, p=0.006). The scores of the LIM_MAMMARY_STEM_CELL, HOEBEKE_LYMPHOID_STEM_CELL and IVANOVA_HEMATPPOIESIS_STEM_CELL signatures were significantly higher in the sh-ZNF498 group compared to the control group（a-c; p=0.038, 0.043, 0.03, respectively）.

Figure. S11

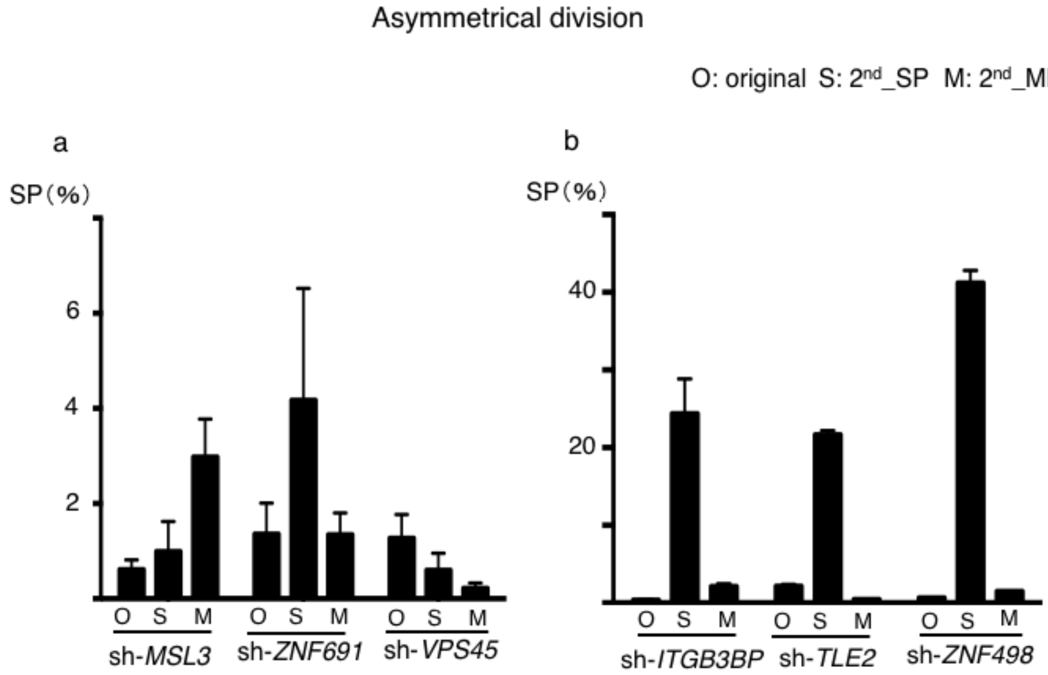

Supplementary Fig. 11. Asymmetric cell division ability was assessed.

a) Analysis of CH1 cells.

b) Analysis of SKOV3 cells. The SP fraction was significantly enriched in 2nd_SP. SP cells can be detected in 2nd_MP cells.

We investigated asymmetrical division capacity. We sorted the SP and MP fractions separately, followed by two weeks culture in normal cell culture conditions, and established 2nd generation-SP and 2nd generation-MP cells, and analyzed the proportion of the SP cells of the 2nd generation. As a result, the proportion of the SP cells of the 2nd generation-SP derived from the three genes suppressed SKOV3 cells were markedly enriched (a). However, the proportion of the SP cells of the 2nd generation-MP cells were

almost the same as that of the original three genes-suppressed SKOV3 cells (a). For CH1, we found that the proportion of the SP cells of the 2nd generation-MP cells was not different from that of the original three genes-suppressed CH1 cells (b).

Figure S12

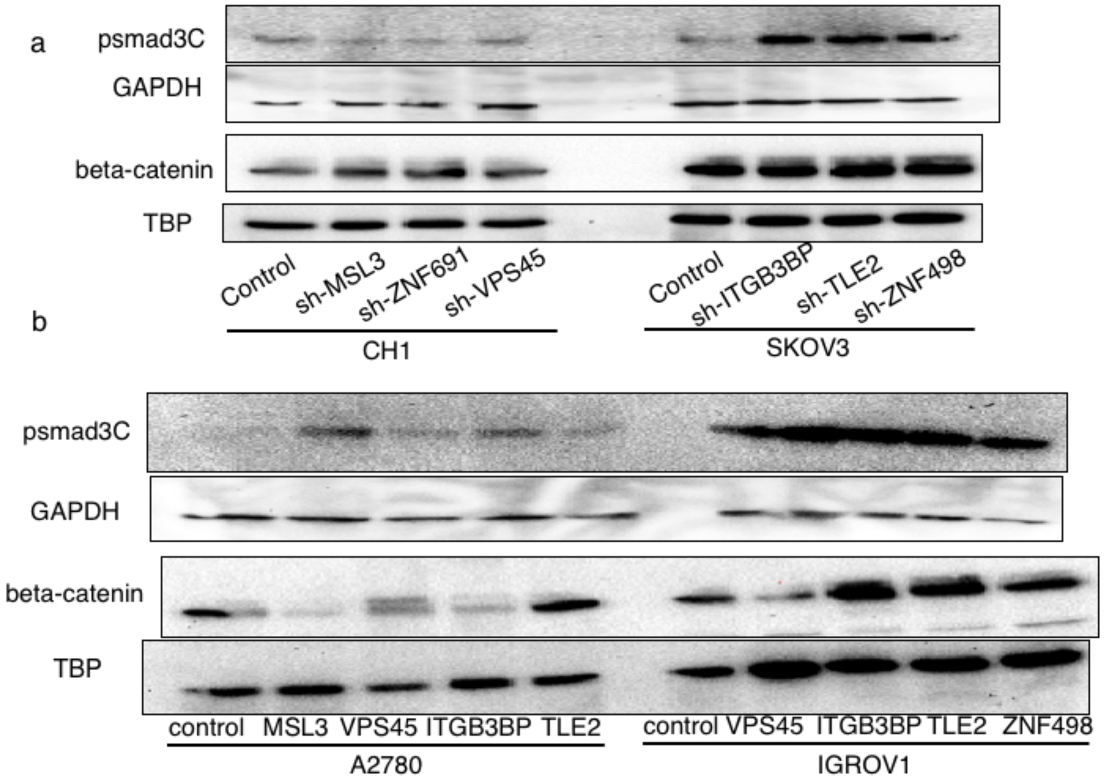

Supplementary Fig. 12. Several of the six target genes regulate TGF-β pathway and Wnt pathway activity.

The TGF-β pathway was assessed by phosphorylation of Smad3C using Western blotting. The Wnt pathway was assessed by β-catenin nuclear accumulation using Western blotting. All experiments were performed in triplicate.

a) Representative image of Western blotting in control cells and cells with repression of sh-target genes for both CH1 and SKOV3. Phosphorylation of Smad3C is markedly increased by suppression of *ITGB3BP*, *TLE2* and *ZNF498* in SKOV3 cells. Slight β-catenin nuclear accumulation was evident with suppression of *MSL3*.

b) Representative Western blotting for A2780 and IGROV1 with control cells and cells with overexpression of target genes. Phosphorylation of Smad3C is not decreased by overexpression of five genes. Overexpression of *MSL3* and *VPS45* markedly decreased nuclear β-catenin accumulation.

The details of western blotting method are descried in supplementary Material and Method.

See Supplementary Figure 14 for full image of the blots.

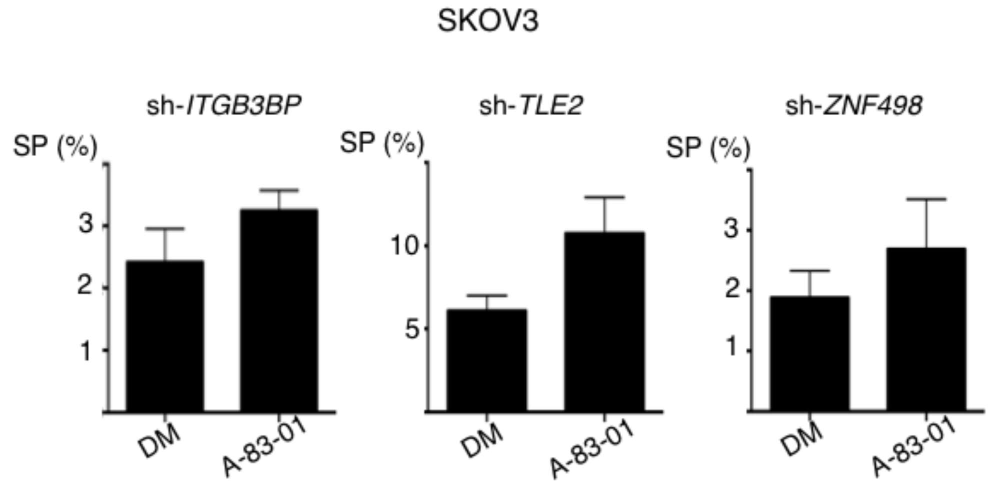

Supplementary Fig. 13. Inhibition of the TGF-β pathway does not decrease the SP fraction that was generated by suppression of *ITGB3BP*, *TLE2* and *ZNF498*.

DM; DMSO (control). A-83-01 (Sigma-Aldrich, St.Louis, USA): Inhibitor of TGF-β pathway, which was used to inhibit the TGF-β pathway.

Figure S14

a psmad3C blot

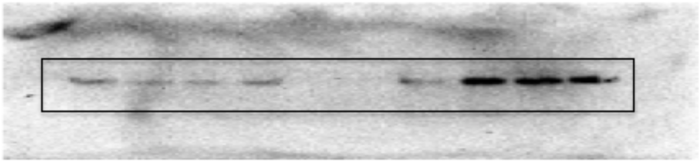

b GAPDH blot

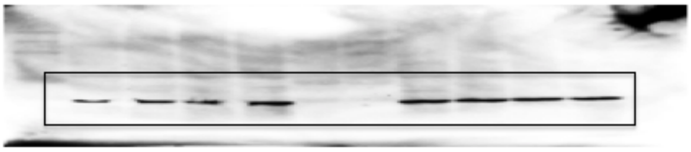

c beta-catenin blot

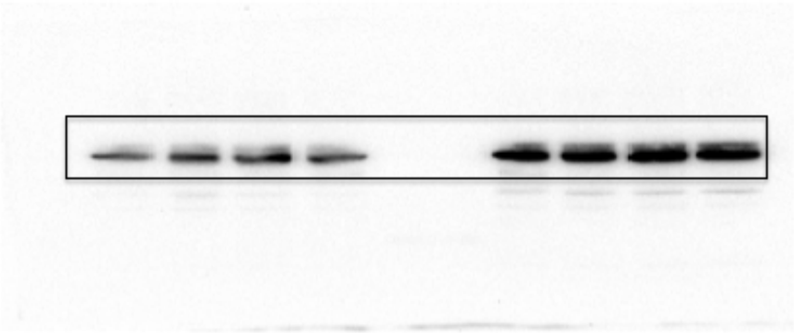

d TBP blot

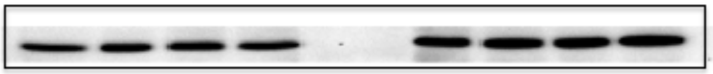

e psmad3C blot

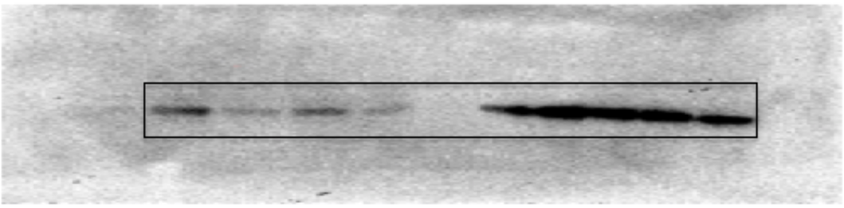

f GAPDH blot

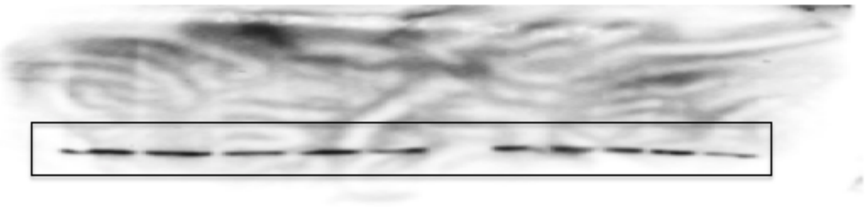

g beta-catenin blot

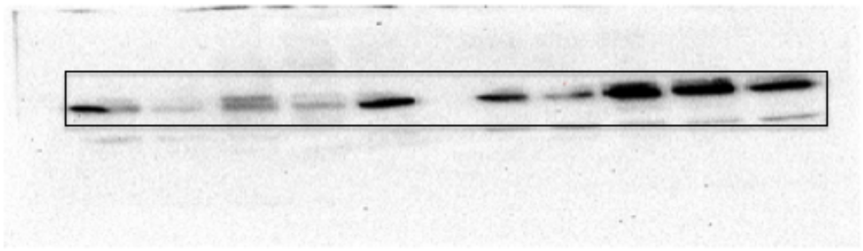

h TBP blot

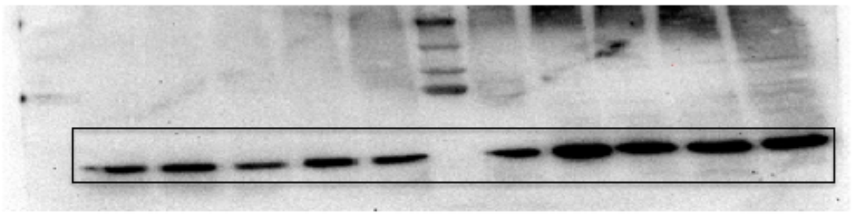

Supplementary Fig. 14. Full images of the blots in Supplementary Figure 12..

Supplementary Table S1: Antisense sequence of 1st screen, and SP percentage in 2nd screen.

a) Datas using CH1 cells

Anti-sense sequence	Target gene	NM number	SP(%)	mRNA check
TAAGAAGTGTAACTCGTCTGG	MAST4	NM_001164664.1	3.79	yes
TTGTAGTGTTTCTCTTCCAGG	ZNF691	NM_015911.2	2.43	yes
TAGCTGACATTCCATGAGAGC	DBH	NM_000787.3	2.19	yes
AGATGAGTTCTAACAACCAGC	VPS45	NM_007259.3	2.01	yes
TCTAGTCTTTCCCAGAGACAG	HSFY2	NM_001001877.1	1.7	yes
AAACACATTGTCAATGTGGCG	MC5R	NM_005913.1	1.64	yes
ATGGATTCAACAAAGGCGGAC	MSL3	NM_006800.2	1.54	yes
ATTCTTAACCACTTGCAGCCA	PRKAG2	NM_024429.1	1.51	yes
TTTCTATGGGCCTCCTAAGTC	GIPC2	NM_017655.4	1.31	yes
TGAGGTAGAGAAAGCAGCAGG	TNFSF9	NM_003811.2	1.24	yes
AGAGCCACTGGTCAGGCTGGC	HAMP	NM_021175.2	1.19	yes
GAAGAAATACATGGGAGTGTG	OR10J5	NM_001004469.1	1.15	yes
TTCTCTTCGAATGAGAAGCGC	MYPOP	NM_001012643.2	1.14	yes
ATGTGTTTAGGATAGAAAGGG	CBX5	NM_012117.1	1.06	yes
ATTGTCAATCTCCACCAGTCG	LMNA	NM_005572.3	1.02	yes
AACTTGGCGTAGATGCTCTGG	RCVRN	NM_002903.2	0.966	yes
ATAGCTGCTGAACTATGATAC	SLC27A6	NM_001017372.1	0.96	yes
ATCTCCTTCTTATGATGAACG	ATPIF1	NM_016311.3	0.86	yes
TACTAGTAGTAACTTAGGTGG	ENPP4	NM_014936.4	0.83	yes
ATGTACTGATTATCAGACGGC	SV2A	NM_014849.3	0.799	yes
AAAGATCTGAAAGTAACACGG	C1orf109	NM_017850.1	0.79	yes
AATTCCACATTGCTTGTGCTG	STOX2	NM_020225.1	0.772	yes
ATGGAGCCACTATCAAGGAAG	FGR	NM_001042729.1	0.75	yes
TTCAGAGTCTTGTATTCCGCG	RBM15	NM_022768.4	0.748	yes
TCATTTGTGTGATCAGGTTGC	KIAA0101	NM_001029989.1	0.74	yes
GTTGGGACCACTGAATCATTG	PPP4R2	NM_174907.2	0.655	yes
CACAAATTCTTAAGGACCGAC	MGAT5	NM_002410.3	0.608	yes
AAAGGCAAATAACAATGAGGG	PPM1H	NM_020700.1	0.56	yes
ATGAGAAGAGTCAAACCATTG	RTN2	NM_005619.3	0.55	yes
ATAAACATCTATCTCACAGGC	RFX1	NM_002918.4	0.51	yes
TATAACCTCGATCATGTTCTC	C9orf64	NM_032307.3	0.45	yes
AACAGGTAACCACTTGAGAGG	SPCS1	NM_014041.2	0.431	no
AATAAACTTGAAGTACCAAGG	GPR87	NM_023915.3	0.4	no
ATAAAGTCCGTAGGACTTCGG	MMD	NM_012329.2	0.38	no
AATCTCAGAAATGATACGGTC	EMILIN1	NM_007046.3	0.37	no
AACTTGTTGAAACCACCTTGG	DUSP7	NM_001947.3	0.33	no
AAGGCAAAGAAGTGGTAAGGG	BDKRB1	NM_000710.2	0.32	no
CTCAAAGCATCAGTCCCATGC	MOB2	NM_001172223.1	0.32	no
ATCACTCTGAATAACTTCTCG	PDIA6	NM_005742.2	0.3	no
ATATCTAGGTAGCCACAGGAG	PPP1R12C	NM_017607.2	0.2	no
AAGATCACTATGAAGAATTGG	RMI1	NM_024945.2	0.19	no

TAGAAGTAGCGGTAGTTCCTC	ZDHHC9	NM_001008222.1	0.142	no
AAGTGCTGACAACGGTGATTC	BAG1	NM_004323.4	0.13	no
TTAGGCCCATTCTCATTGTCG	CRTAC1	NM_018058.4	0.086	no
TTGAAGTTAGAGATTGCCCTG	METTL7B	NM_152637.2	0.075	no
TAGAGACCCTTATTGAAGTAG	SFXN2	NM_178858.4	0.065	no
TAGCTGTGAGAAGTCATGTGC	ZNF488	NM_153034.2	0.06	no
AAAGGATTATTCCAATGAGGG	SLITRK4	NM_173078.2	0.043	no
ATGTCCCGACTTTCAGAAGGC	DRD5	NM_000798.3	0.038	no
GAGATGAAATTGCTGAGGAGG	SYT7	NM_004200.2	0.036	no
AAGTGTCCTTTGCATACTGGC	TRIM13	NM_001007278.1	0.035	no
CTCACGAGGGAGTATTTCTTG	ZC3H3	NM_015117.2	0.023	no
AATAGGAGAATTTGCTTTGGC	HYDIN	NM_017558.2	0.018	no
AAACCCTGTCGTGTATAGTGC	AFF1	NM_001166693.1	0.011	no
ATGTCGACAAATTTAGTTCAC	CCDC59	NM_014167.4	0.01	no
AAGCTCATCAGCTTAGGGATG	CCDC80	NM_199511.1	0.01	no
AAATGATTCATCGTCAGAGTC	CDCA7	NM_031942.4	0.01	no
TAAGGCAGAAGATGTAGAGCG	CDKN1A	NM_078467.1	0.01	no
TACTCAATCTTCAGGCTTGGG	CIB4	NM_001029881.1	0.01	no
ATTACATTGCAGTTTCCCGGG	CORIN	NM_006587.2	0.01	no
TTACCATAGAGTCTGGCTTTC	DYNC1LI2	NM_006141.2	0.01	no
TTATTAAGGTGCACACCCATG	EFS	NM_005864.2	0.01	no
ATTTCCCATCATTAAGAAGGC	FAM91A1	NM_144963.2	0.01	no
TAGATTTCCGGGATACAGCAC	FHOD1	NM_013241.2	0.01	no
TTTAACCTCATCTTTAGAGAG	FKBP14	NM_017946.2	0.01	no
CTTTAACCTCATCTTTAGAGA	FKBP14	NM_017946.2	0.01	no
ATCTGCTTGTCAATCACATGG	GFPT2	NM_005110.1	0.01	no
AACCAAACGCAACCTGCAGTG	GMCL1	NM_178439.3	0.01	no
CATTTACAACGGGCATTTGGC	KIAA0528	NM_014802.1	0.01	no
ATGTTGATAATTTCTTGGTGG	NDUFB10	NM_004548.2	0.01	no
TTCATAGACCCAGTCATGAGC	NME1-NME2	NM_001018136.1	0.01	no
ATACAGATCAATGAGCCCTGC	OAS2	NM_002535.2	0.01	no
TGCCTTAGCAAACTCTAGTCG	RBPMS	NM_001008710.1	0.01	no
TACAGCCACCATGTTTAAGGC	RIOK2	NM_001159749.1	0.01	no
ATAGACGAAGATGGAAACGGG	SCYL1	NM_020680.3	0.01	no
ATACTGTTCAAATTCCGTGCG	SOX17	NM_022454.3	0.01	no
ATTCTAAAGATTCCACTCGGG	SPAG9	NM_001130528.2	0.01	no
ACTAACCTTGTAGGAGAGCTG	SUN2	NM_015374.1	0.01	no
ATACCTCATAAGTTTCCTTTG	TBC1D1	NM_015173.2	0.01	no
CAATTCCACATTGATCGTGCG	TECTA	NM_005422.2	0.01	no
AATTCCACATTGATCGTGCGG	TECTA	NM_005422.2	0.01	no
TTCCAACTGAAAGATTTGAGC	TLR7	NM_016562.3	0.01	no
AGTGGCCAGAACATTCTCATC	WDR55	NM_017706.4	0.01	no
ACTTACTACCAACAGCTAGGG	WIPI2	NM_016003.3	0.01	no
TATAATCTGCTGATACTGTTG	YLPM1	NM_019589.2	0.01	no
ATTCTGAGTGAAACTTCTTTC	ZKSCAN3	NM_024493.2	0.01	no

ATTTGTAAGCTCAGGTATGGG	ESRRB	NM_004452.3	0.001	no
TTTAGCATCTTCTTTGAGTTC	EVC	NM_153717.2	0.001	no
TAGACTACCTCTCACACCTGG	GPR153	NM_207370.2	0.001	no
TTGGTATAAGTGATGGTGCGC	KIAA1024	NM_015206.2	0.001	no
AAATTGTCTGTATCTGACTGA	TBR1	NM_006593.2	0.001	no
TAGCTGGTGAATTTAACTGAC	TMPRSS11D	NM_004262.2	0.001	no
TTAGTAGTATTTCCTTCAAGC	TNFRSF1A	NM_001065.2	0.001	no

	SP(%)
control-1	0.122
control-2	0.053
control-3	0.084
control-4	0.0483

b) Datas using SKOV3 cells

Anti-sense sequence	Target Gene	NM number	SP(%)
TTTCTGGAGATGTCACAACTC	TLE2	NM_001144761.1	23.1
TTAGCCAGATATTGTTGTGCG	C14orf119	NM_017924.2	9.93
TTCATAGCTGTCAAGATGACG	ITGB3BP	NM_014288.3	7.5
TTATAGGGCATAGACTCGAAG	SHMT2	NM_005412.4	3.93
ATACCTGAGCTATAACTGGTG	ZNF235	NM_004234.4	3.3
CATTTCTGCGGGATGCCGTTG	CELSR1	NM_014246.1	3.18
AATATAGGGCATTAATACTAG	ZNF498	NM_145115.2	2.57
TAGAGCTGTGTGTTTGTACTG	FAM175A	NM_139076.2	2.36
ATAATGACCAAGCTGGTGAGG	CYB561D2	NM_007022.3	1.9
AATAGGAGAATTTGCTTTGGC	HYDIN	NM_017558.2	1.87
TAGCTGGTGAATTTAACTGAC	TMPRSS11D	NM_004262.2	1.77
AATCAAATTCCATAACAGGTC	XPA	NM_000380.2	1.77
TTCCAGAATTTCTCCATAGGC	NDUFB6	NM_002493.3	1.55
TTCTGGTGCCACTGTTGTATC	CES3	NM_024922.4	1.49
AATGCAGAATCCTTCTTTCAG	MRPL16	NM_017840.3	1.22
CTTGAAGTGTGGATCTAGGTC	CSNK2A2	NM_001896.2	1.17
TAGATTTCTGTTTAGGTGGCG	SRRM2	NM_016333.3	1.08
TCCTTCTCCATATTTCACTGG	ITLN1	NM_017625.2	1.07
AATATGATGGTGAGCTTCCGG	HFE2	NM_145277.4	1.06
ATTGGATTTCTGTTGCGTTTC	CSH1	NM_001317.3	0.9
AGCTGAGATGACTTTCACTGG	ODF2L	NM_001007022.1	0.9
TTATTCCTATTCCATCATTGG	UFM1	NM_016617.2	0.89
AAACCACTCGAATAAGTCAGG	CRELD2	NM_001135101.1	0.86
CATCTTGGAACACAGGAGCTG	HS6ST3	NM_153456.2	0.74
TTGAAGCCTCACAACATTGGC	KIF11	NM_004523.3	0.728
TATAGTCGGCTTTCAGCCGGG	KIF17	NM_001122819.1	0.69
TACAAGTAGAATAACACTCAG	BAGE	NM_001187.1	0.64
TTGCCGTGGCCCTTAACCTGG	HBA1	NM_000558.3	0.64
TTCTATTCTGTGGAGTATGGC	IRF9	NM_006084.4	0.59

CATTTCGAACCACAAGAATCA	PTRH2	NM_016077.3	0.57
TGGGTAGAAATTCAGAATGGC	CCT6B	NM_006584.2	0.52
ATTTAAGCTGTCTGCCATGCG	EGLN2	NM_053046.2	0.51
AACCACCCTAGTCACATAAGG	CELA2A	NM_033440.2	0.47
ATAGAACAGTAAAGGGAGAGG	PAN2	NM_014871.2	0.459
ATTCGCTTAAATGATGCTTGC	MAPRE2	NM_014268.1	0.456
ATAGACAGTGAGACAGTGAGC	GPR142	NM_181790.1	0.45
GTTGGGACCACTGAATCATTG	PPP4R2	NM_174907.2	0.42
ATCACGATGCTGTTGTCTCTG	DHX38	NM_014003.3	0.41
TTCAACTCTCTTCATTGCATC	RSL24D1	NM_016304.2	0.39
ACCTTGGCTAAACTGGCATCA	LRRC29	NM_001004055.1	0.361
TTGTCTATCTTTCCTTGATTC	TCEAL2	NM_080390.3	0.355
ATGACCTTCCTCAGTAGCTTG	CCL27	NM_006664.2	0.34
TGACAAAGAATGAGTGTGCTG	RP1	NM_006269.1	0.32
AAGAGTATGGTGGTCTTATCA	AMELY		0.3
CTAAGATGTGTGCTCCGGCTA	ZNF391	NM_001076781.1	0.294
AATAAACGTGCAAGGCATCTG	CASP14	NM_012114.1	0.26
TTGACTATCACTTGTAGGTGG	SCHIP1	NM_014575.2	0.26
TTGTTACGCATAATTTCCGTC	SCHIP1	NM_014575.2	0.26
TTCCTATCTGCAATGTAAAGC	SEC63	NM_007214.4	0.26
TAGATCAAGCTGTATCCATGC	ZNF527	NM_032453.1	0.258
TTAAGACCGATATTCCTGAGC	PLCL1	NM_001114661.1	0.25
TTGATGGTGTGGTCTGACAGC	FCHSD1	NM_033449.1	0.2
ATCAGTTGTTTCATTCGCCCA	PDIK1L	NM_152835.3	0.2
ATTGGTAATGCACACAGAGAG	C9orf46	NM_018465.2	0.19
AACTTGGTGTACTTCTTGAGC	CHST12	NM_018641.3	0.175
AAGTAGAGGCAGATGGTAAGC	CHMP2B	NM_014043.3	0.17
CATACCCTAGCTCGGATGGGA	FKBP2	NM_001135208.1	0.17
TAAGAGATGATTTATCTCTGC	RGSL1	NM_001137669.1	0.17
AAGAAGGGTCAGCTGAAGAGC	C2CD2L	NM_014807.3	0.16
TTCCAGGACCTTGTATTCTGG	ELL3	NM_025165.2	0.15
ATCTCTCAGTTTCCTTGATCG	GDA	NM_004293.3	0.15
TTGTTGGCATAAACTGTAGTC	PON2	NM_000305.2	0.15
AAAGATGTTAAATGGCTTTGC	TKTL2	NM_032136.4	0.15
AATCTTAAATATGTTGCCCAG	PTPRR	NM_002849.2	0.148
CAATGCGGCCATCTTGTCATC	ADAMTS4	NM_005099.4	0.124
TTCTTGGTATGTTTGCTCAGG	TMTC4	NM_001079669.1	0.11
AAGATTAGATATGGAGGCAAC	USP39	NM_006590.2	0.11
ATTTCTGAGCTATGCCTGGTG	CTTNBP2	NM_033427.2	0.107
AAGTAGGGAAGGTGCAAGGTG	RNLS	NM_001031709.2	0.103
AATATGGACCAGAGCCAAGAG	OR2A12	NM_001004135.1	0.1
TAGAGGTCGCAGTCGTAGAGG	CHST1	NM_003654.3	0.099
AAAGGCAAATAACAATGAGGG	PPM1H	NM_020700.1	0.092
TTTGTTAGTTTAAGAACCTGG	NHS	NM_001136024.2	0.09
AAGGGAAGCTCTTTCTAGCTC	UTS2	NM_006786.3	0.09

TCTCTACTTTAGGTCACCTCG	C16orf72	NM_014117.2	0.089
ATAAACTCGTTGAAGCGTTTG	PIP4K2B	NM_003559.4	0.085
AAAGGCATCATCCTGGATGCT	CABC1	NM_020247.4	0.081
ATAAAGTCCGTAGGACTTCGG	MMD	NM_012329.2	0.071
TTCTTTGAAATTCGTCTTGGG	CENPC1	NM_001812.2	0.07
TATAGGCTGCTGCTGGTTGGG	YBX2	NM_015982.3	0.064
AGACCTATACAGAAGCTGTCG	SFI1	NM_001007467.1	0.06
CTTTCCATGGTAACGACACAG	PDSS2	NM_020381.3	0.057
CACAAATTCTTAAGGACCGAC	MGAT5	NM_002410.3	0.052
AATGGCACAGTTTATCACGAC	ENAH	NM_001008493.1	0.05
CCATAGTAAGTGTTGACCAAA	UBIAD1	NM_013319.2	0.044
ATTAGCTTGTATACACGTGGG	DCUN1D5	NM_032299.3	0.04
TTGGTGAGGTTTAGCTTACTG	MAGEB1	NM_002363.4	0.04
ATCTTGGACGTGAAGAAGTGC	PLXNA1	NM_032242.3	0.039
AAGGTGGATAAGTACTTGGTC	TRIM17	NM_001024940.2	0.038
TTCACTGAGCTGGAAGGCGGC	MESP2	NM_001039958.1	0.036
TTGACATTAGTGTGTAGCTGG	VDAC2	NM_003375.2	0.031
AAACAGGAAGGTAAATTAGAC	OR4D9	NM_001004711.1	0.03
TTAAGGCGGCTATTGTATTGC	DGKK	NM_001013742.1	0.0229
ATGACAACAGTCTTGACCAGG	LPAR2	NM_004720.5	0.02
AATATTTGACTACATGAGGGC	STK4	NM_006282.2	0.02
AATTGGAACTTCATTGGCATC	GATM	NM_001482.2	0.013
AATGCTGCTTAGACCAGTTGG	AIM2	NM_004833.1	0.01
TAATGCTCCCAATGCTACTCG	ARID3B	NM_006465.2	0.01
AAGAAGGAAACATTCAATAGG	C1orf109	NM_017850.1	0.01
CAATATCCAGGAGTGACTGCG	CYS1	NM_001037160.2	0.01
ATCTGAACAACTTGATAACCG	FASTKD3	NM_024091.2	0.01
TACCTTTAATAGTTTCTCTGC	ITM2B	NM_021999.2	0.01
AGTGTTTCAACAACTGAGAGG	LAMP2	NM_002294.1	0.01
ATAGATTCCCAGGAACAGGAG	OR8D1	NM_001002917.1	0.01
TACATGGCAGATCGGGAATAC	RPL6	NM_001024662.1	0.01
TGCCCTACAGCAATTTCTGAG	SLC25A35	NM_201520.1	0.01
TTGAGCTCCGACTGGTTGTGG	SOST	NM_025237.2	0.01
TTCATCCAGCTTAACAATGGC	SPTAN1	NM_003127.1	0.01
TTTCCATTCCATATCATCAGG	SUGT1	NM_001130912.1	0.01
AAATGCCGACACAAACCACTC	TRAPPC2	NM_001011658.3	0.01
CAACAATCACCATATCGTCAC	UQCRFS1	NM_006003.2	0.01
AAGGTTACTGAGTTCTGTGAC	ZNF177	NM_001172650.1	0.009

	SP(%)
control-1	0.086
control-2	0.066
control-3	0.055
control-4	0.074

Supplementary Table S2:
Gene list significantly downregulated or upregulated by suppression of 6 genes.

(A) Genes significantly downregulated by sh-MSL3

Gene list	log2-fold-change
FOSB	-4.18191
FOS	-2.25775
A2M	-2.00255
OLIG3	-1.6759
FHDC1	-1.35521
EGR1	-1.08108
IER2	-1.07422
JUN	-1.00886
DHRS2	-0.841775

(B) Genes significantly upregulated by sh-MSL3

Gene list	log2-fold-change
CXCL14	3.91678
HAPLN1	3.78177
TNFRSF11B	3.09781
CER1	2.99185
PSKH2	2.98947
COL3A1,MIR3(	2.81225
RASGRP3	2.62125
CDH11	2.60686
LGR5	2.59575
ISM1	2.53031
WNT9B	2.4567
MIXL1	2.4564
HAS2	2.10645
IGFBP3	2.02902
IL8	1.97846
HEY1	1.75901
HAND1	1.75241
DKK1	1.74624
MSX1	1.74486
LIPG	1.68959
SNAI2	1.64438
ITGA9	1.59375
BMPER	1.58026
NRP1	1.46298
GPR124	1.41164
FN1	1.35286
FLNC	1.31755
APLNR	1.25522

SULF1	1.22959
VGF	1.1369
COL6A3	1.13451
COL2A1	1.12277
BAMBI	1.05446
RDH10	0.978624
ADAMTS9	0.87749
COL6A1	0.836

(C) Genes significantly downregulated by sh-ZNF691

Gene list	log2-fold-change
FOSB	-7.20419
FOS	-4.69166
TOX	-2.91714
KLF4	-2.87287
MIR1263	-2.40381
DUSP1	-2.30706
ARC	-2.19869
EGR1	-2.16924
SLC27A6	-2.06807
GDF15	-1.99919
CYP26A1	-1.84019
JUN	-1.78372
EGR3	-1.70323
IER2	-1.55566
PMAIP1	-1.49063
EGR2	-1.47525
CACNA1H	-1.43445
ZNF516	-1.4296
LEFTY2	-1.34619
UNC13A	-1.33111
SNHG7,SNOR/	-1.32933
ZFP36	-1.32089
MYH6	-1.24264
HTR7	-1.21323
CDKN1A	-1.18198
CYR61	-1.15118
SLC40A1	-1.08015
ANKRD1	-1.07874
NTS	-1.06782
POU5F1	-0.981822
PPP1R15A	-0.978497
PLK2	-0.959135
EHD3	-0.874311
OR52N4,TRIM:	-0.83255

PRDM14	−0.817699
BTG2	−0.800708
SERPINE1	−0.792314
CTGF	−0.776492

(D) Genes significantly upregulated by sh-ZNF691

Gene list	log2-fold-change
HAPLN1	3.06761
RN7SK	2.86994
COL3A1,MIR36	2.37753
RPPH1	2.16088
MEOX1	2.15139
RMRP	1.98027
SNORD3A	1.84055
MSX1	1.33546
RHBDL3	1.26524
NOG	1.25016
HTRA1	1.21944
COL2A1	1.21517
FRZB	1.19575
NR2F1	1.14323
CDH11	1.11788
LRRN3	1.067
ARL4C	1.05996
TAGLN	1.02811
DACT1	1.01941
RN7SL1	0.989157
LRP2	0.984936
GREM1	0.971817
SORBS2	0.957809
RN7SL2	0.943898
LIX1	0.942753
SOX9	0.936796
ITGA9	0.932969
IGFBP5	0.918005
SLC7A11	0.915311
RGS5	0.914356
FZD2	0.879295
RDH10	0.867611
FGFBP3	0.856321
ATP4A	0.828382
ILDR2	0.802873
COL11A1	0.796979
HUNK	0.77926
PAX3	0.775382

DISP1	0.761682
ID3	0.750814
PKDCC	0.745005
PTCH1	0.735864
HYMAI,PLAGL'	0.651824
ID2	0.645869
DSP	0.616858

(E) Genes significantly downregulated by sh-VPS45

Gene list	log2-fold-change
FOSB	-7.68599
FOS	-4.94366
ANKRD1	-4.19894
HOXB9	-4.14594
PCDH7	-4.01605
FMOD	-3.72444
MYL7	-3.42664
MYLK3	-3.30605
SEMA6D	-3.05695
DGKB	-2.96941
LEFTY2	-2.79322
KLF4	-2.75035
SH3GL2	-2.53431
SLC27A6	-2.49722
KRT19	-2.4812
EGR1	-2.4359
CYP26A1	-2.42114
EGR3	-2.3515
TNF	-2.34463
DUSP1	-2.31374
HAND1	-2.28721
SLC40A1	-2.22794
EGR2	-2.19082
ACTC1	-2.16748
BMPER	-2.1538
LOXL4	-2.07291
ARC	-2.01035
CYR61	-1.98109
GATA6	-1.97977
COLEC12	-1.97523
JUN	-1.90003
GATA4	-1.88096
LHX1	-1.82141
ADAMTS6	-1.7594
DLL3	-1.75619

IER2	-1.74324
ALPK2	-1.73871
UNC5C	-1.73579
PLXNA2	-1.63756
H19,MIR675	-1.58821
COL1A2	-1.58728
ZFP36	-1.58397
COL13A1	-1.53157
SLC7A14	-1.52878
LIPG	-1.51586
CTGF	-1.49239
CER1	-1.46372
HTRA1	-1.42278
GDF15	-1.41763
SOST	-1.34637
FAM89A,MIR1'	-1.30874
DLX5	-1.30293
COL2A1	-1.29453
MYH6	-1.28147
IGF2,INS,INS-I	-1.20574
RGS16	-1.19584
BMP4	-1.18937
FBN2	-1.10327
RMRP	-1.0688
VGF	-1.03039
FOXC1	-1.01329
PPP1R15A	-0.943602
FN1	-0.942187
IER3	-0.907062
PCDH10	-0.9053
DUSP6	-0.882344
SAT1	-0.870932
APLNR	-0.864792
SERPINE1	-0.808872
ENC1	-0.791225
ANKRD33B	-0.776967
ADAMTS12	-0.757723
RHOB	-0.682941

(F) Genes significantly upregulated by sh-VPS45

Gene list	log2-fold-change
CA9	1.82724
EPHA3	1.60351
STC2	1.49174
GPC6	1.27863

PRDM14	1.11244
PDZD4	0.999355
OLIG3	0.877604
PFKFB4	0.771154
DPYSL3	0.682752

(G) Genes significantly downregulated by sh-ITGB3BP

Gene list	log2-fold-change
ANKRD1	-2.05243
PDIA5	-1.65339
HR	-1.44687
SMR3B	-1.40264
LMLN	-1.23999
PSTPIP2	-1.16588
CYP24A1	-1.15361
PPYR1	-1.11689
TRIM31	-1.02658
TCF4	-1.01072
SNCG	-1.00987
CLIC3	-0.970617
FNDC3A	-0.954996
C1orf51	-0.9483
DCAF12L1	-0.933145
TFPI2	-0.92686
C22orf32	-0.923105
SDPR	-0.896883
TMED8	-0.889374
DDAH1	-0.871154
PLOD2	-0.866413
KRT7,LINC005	-0.865006
APOL3	-0.821783
IL1B	-0.806726
SELK	-0.770966
RRAD	-0.770155
CDH16	-0.752899
AMMECR1	-0.742981
C1QL1	-0.739194
PER3	-0.730809
TEF	-0.711282
PDE3A	-0.710818
NCAM2	-0.709616

USO1	-0.703489
PRKCH	-0.697559
KDM5C	-0.695136
COX17	-0.691002
SLC25A4	-0.687359
NEK7	-0.664502
GAS2L3	-0.641248
FAF2	-0.62412
PNMT	-0.623833
PHGDH	-0.622662
UNC13D	-0.606739
HABP4	-0.600667
ZNF781	-0.592096
ZNF681	-0.590637
ARHGAP18	-0.581491
FAM96A	-0.577954
UBE2L6	-0.573912
FGF2	-0.561123
PSMD1	-0.550202
WDR37	-0.544056
SEMA4B	-0.542844
THBS1	-0.541295
MALL,NPHP1	-0.538007
NNMT	-0.531081
SLC25A30	-0.526159
MMP1	-0.518944
RPL7L1	-0.516534
ARL6IP5	-0.515401
CDV3	-0.48092
AFAP1L2	-0.480565
STRADB	-0.479903
GM2A	-0.478326
ZNF486	-0.470985
ACLY	-0.470924
KIAA1161	-0.467725
ADCY9	-0.465931
GTSE1	-0.460251
IRF1	-0.457724
COL5A2	-0.454961
PMEPA1	-0.451757

CD2AP	-0.444223
SNX6	-0.440644
KRT19	-0.437081
PDZK1IP1	-0.436235
CES2	-0.429953
APOL1	-0.412931
RUVBL2	-0.410636

(H) Genes significantly upregulated by sh-ITGB3BP

Gene list	log2-fold-change
MIR2682	5.56676
SNHG11,SNOF	4.42231
SERPINB7	3.70128
ST8SIA6	1.91257
IL33	1.56824
NTNG2	1.5664
PARM1	1.47642
CORO2B	1.47444
DNER	1.45377
NDNF	1.45333
PI16	1.44019
SCEL	1.42307
EREG	1.41966
TMPRSS4	1.35359
IL13RA2	1.33741
RN7SL2	1.33465
PAX2	1.20716
CPAMD8	1.17514
PRDM1	1.17122
CXorf22,CXorf3	1.16835
CD69	1.15797
COL8A1	1.06633
OSCAR	1.02886
JPH1	1.02744
TP63	1.01335
SLC22A17	1.01007
WDFY4	0.987767
NCALD	0.978979
PPARGC1A	0.975126
MEOX1	0.97466

RN7SL1	0.968892
FLRT2	0.957731
MAPK8IP2	0.956275
THSD4	0.954543
AREG	0.92796
CCDC64	0.922365
SERPINA1	0.88819
WNT5A	0.85809
FOXQ1	0.847851
DACT1	0.834616
LBH	0.834042
ARL14EPL	0.828242
ZNF423	0.80379
BACH2	0.789203
FAM198B	0.773284
SIX3	0.766906
INHBB	0.766513
C9orf47,S1PR3	0.765652
LIPG	0.748481
FNDC1	0.739001
DPP4	0.737751
MCAM	0.732712
BOLA2	0.719968
C14orf132	0.716352
TMTC1	0.707114
LRRC38	0.696591
ELFN1	0.691723
NOV	0.648431
KCNJ16	0.645769
PGBD5	0.639343
TNFRSF11B	0.633991
C1orf116	0.631904
ARNTL	0.628596
SAMD12	0.628589
CLDN16	0.627735
MTRNR2L8	0.622668
AIM1	0.608147
TMEM47	0.60778
SMOC2	0.605006
CRABP2	0.603325

LIN28A	0.598212
C1S	0.597727
SLC1A3	0.593477
SEPT3	0.592115
SLC36A1	0.590216
NPAS2	0.588527
NOTCH3	0.583231
KIAA1211	0.570221
CPS1	0.568441
LAMC2	0.561917
DUSP5	0.556892
C2orf72	0.550712
GPR110	0.547833
NDRG4	0.543185
IGFBP6	0.540565
PEG10	0.52883
SEMA6A	0.525957
PLSCR4	0.52542
VNN1	0.521925
SEL1L3	0.520235
PGPEP1	0.514597
EDN1	0.506976
MAL2	0.506565
BAI2	0.498963
FERMT1	0.496004
GPRC5B	0.489996
AKAP12	0.489536
PXDN	0.480641
MICAL2	0.478865
CDH5	0.473065
TUB	0.435212
TNFSF10	0.426957
HSPA1A	0.421592

(I) Genes significantly downregulated by sh-TLE2

Gene list	log2-fold-change
VNN1	-4.37179
AQP3	-4.08992
IDO1	-4.01947
C12orf36	-3.95307

VNN2	-3.90216
MEFV	-3.81621
LTB	-3.79239
TM4SF18	-3.65406
TM4SF18	-3.65406
HLA-DMB	-3.44505
LOC10013041	-3.39162
SAA2,SAA2-SA	-3.2952
DHRS3	-3.2918
MUC1	-3.26725
C8orf4	-3.24409
ALOX5	-3.23775
AKR1C1	-3.19765
CLIC3	-3.12111
MAL	-3.08857
LOC10050632	-3.07431
MMRN2	-2.85374
IL1R2	-2.8002
ICAM1	-2.79104
SMPDL3B	-2.74211
GOLT1A	-2.69101
TM7SF2	-2.67388
TNFSF10	-2.65972
CLDN10	-2.63356
KCNQ1	-2.6007
LOC113230	-2.57399
GBP2	-2.55418
IGFBP3	-2.51624
SERPINA6	-2.50714
KIF6	-2.49922
BASP1P1	-2.49804
BASP1P1	-2.49804
PRKCG	-2.49545
G0S2	-2.48563
PCSK4	-2.46641
SEPP1	-2.44493
AKR1C2	-2.39667
ALDH3A1	-2.3835
MMP7	-2.37314
CRYM	-2.35323

SOD2	-2.35208
CA11	-2.34262
HRASLS2	-2.33688
WISP2	-2.33649
CYP26A1	-2.32434
LEMD1,MIR135	-2.32166
CASP1	-2.32056
ZNF323	-2.31401
SGPP2	-2.30748
LIPC	-2.30108
LIPC	-2.30108
AKR1C3	-2.29396
SLC22A18	-2.2935
RNF144B	-2.2897
DDIT4	-2.28757
SDPR	-2.28166
HSD17B14	-2.27157
CDH16	-2.25662
SHISA3	-2.23988
CALHM3	-2.23859
PBXIP1	-2.2313
IL33	-2.22574
SLC14A2	-2.21246
APOL3	-2.20847
TLE2	-2.19856
IFIT2	-2.19804
CEBPD	-2.19776
IFIT1	-2.1972
SAA1	-2.19713
RASAL1	-2.19382
MPZL2	-2.18783
PPL	-2.17867
ADHFE1	-2.13255
GPR110	-2.11603
MMP1	-2.08901
LOC10050608	-2.08564
PRPH	-2.08186
CLDN3	-2.07718
LOC10050600	-2.07525
PP13	-2.07456

CP	-2.0705
SNCG	-2.06285
LOC10050765€	-2.05506
C7orf29,LRRC€	-2.05393
CFH	-2.03813
IL1B	-2.0359
KIF12	-2.03317
EPSTI1	-2.0218
LOC10013155'	-2.01411
MMP28	-2.00569
WFDC2	-1.9957
NR3C2	-1.97482
REEP6	-1.96324
C2orf88	-1.96103
LGALS9	-1.95661
SULT1C2	-1.95063
SULT1C2	-1.95063
CEACAM20,CE	-1.94804
RARRES3	-1.9459
CDK18	-1.92288
LMO1	-1.91502
TRAPPC6A	-1.91351
FLRT3	-1.8935
CDH1	-1.88258
DNAJC22	-1.8821
C1QTNF1	-1.88079
LOC10099645'	-1.86576
BMF	-1.84962
PARK2	-1.84776
PNMT	-1.84288
KIAA1211L	-1.84208
IL1R1	-1.83589
GPM6A	-1.83166
SLC44A5	-1.83158
NXNL2	-1.82957
ANXA4	-1.81221
NTSR1	-1.80298
C3	-1.79959
RCAN2	-1.79806
SSPN	-1.78804

OCEL1	-1.78509
GBP5	-1.78427
YPEL3	-1.78143
UGT1A1,UGT1	-1.7812
TNFRSF11B	-1.7778
CYP1B1-AS1	-1.77438
CYP1B1-AS1	-1.77438
ITGB8	-1.76827
CFB	-1.76388
CA12	-1.76295
NEURL3	-1.75629
DDIT4L	-1.75436
TRIM2	-1.74972
YPEL2	-1.74918
LOC440461	-1.74846
STAP2	-1.74814
CYP1B1	-1.74053
OR1N2	-1.73595
PRR15	-1.73431
TCP11L2	-1.73001
BAIAP2L2,PLA2	-1.72274
TMEM45B	-1.71828
ASS1	-1.71547
TNFAIP2	-1.71294
LOX	-1.71171
GPRC5C	-1.69924
TMEM47	-1.68958
CALCOCO1	-1.68136
HIST1H2AI,HIS	-1.68096
SH2B2	-1.66909
B3GNT3	-1.66582
DSCAML1	-1.6658
WDR96	-1.66414
IMPA2	-1.6637
PDK4	-1.66202
HIST1H3E	-1.65731
DICER1-AS1	-1.65693
ALOX12	-1.65589
C1RL	-1.65273
QPRT	-1.64762

HID1	-1.64213
PARD6A	-1.64208
ELF3	-1.64111
ALDOC	-1.62511
C1S	-1.62449
DYRK1B	-1.62316
LHPP	-1.62283
FAM20C	-1.62103
PBX1	-1.62003
PNRC1	-1.61299
HSD11B1L	-1.60948
RNF208	-1.60913
ABCA3	-1.60189
CXCR4	-1.60186
C8orf34	-1.60154
SAT1	-1.58401
SAT1	-1.58401
SPEF1	-1.58102
AK8	-1.58045
RAB26	-1.57804
MIR4800,MXD4	-1.57697
DNAJC28	-1.57633
CLEC11A	-1.57396
EDIL3	-1.57392
SPTLC3	-1.5719
IL1A	-1.56938
PLLP	-1.56533
LSAMP	-1.56151
TSPAN7	-1.55845
ACBD4	-1.54942
BTBD19	-1.54722
THBS4	-1.54671
CXCL2	-1.54427
MACROD2	-1.54304
MACROD2	-1.54304
GNG7	-1.53645
CASP10	-1.53469
LOC10050766[illegible]	-1.53172
C2orf15	-1.53026
CYB5R3	-1.52995

WNT10A	-1.52625
ETV7	-1.52405
ETV1	-1.52105
TACSTD2	-1.51581
CSF1	-1.51567
KLHL24	-1.51426
CREBRF	-1.51294
LMO2	-1.49545
PDZK1IP1	-1.49401
NOV	-1.48465
DCAF12L1	-1.48456
C6orf223	-1.48257
MIR210,MIR21(	-1.48083
CTSF	-1.47887
SMOC2	-1.47533
FLJ22184	-1.47252
BMS1P5	-1.46989
SLC16A2	-1.46799
CERS4	-1.46785
SLC2A12	-1.45938
DNAAF3,TNNI3	-1.45851
IER5L	-1.45649
SOD3	-1.45018
FTCD	-1.44874
CGNL1	-1.44303
ITGB4	-1.43893
FBXO32	-1.43721
NDRG1	-1.4352
CITED4	-1.43231
C17orf103	-1.42694
TUBB4A	-1.42598
COL16A1	-1.41853
AMTN	-1.41829
DBP	-1.41636
RSPH1	-1.41246
BCAT1	-1.399
GPX3	-1.39673
OSCAR	-1.39586
CNFN	-1.3909
FP588	-1.38623

SRCIN1	-1.38538
C11orf70	-1.3821
ZFYVE28	-1.37831
TRIB2	-1.37749
OAS1	-1.37628
CRB3	-1.37422
ABCC3	-1.37399
ACSS3	-1.37137
GATSL3	-1.3712
KYNU	-1.37069
CAMK2B	-1.37054
LOC10050763[illegible]	-1.36897
ZC3H12A	-1.36856
LOC728730	-1.36245
FOXA2,LINC0[illegible]	-1.36142
INSL3,JAK3	-1.36094
FAM66C	-1.3577
ALDH3B1	-1.35708
SMARCD3	-1.34989
TSPAN1	-1.34931
ADC	-1.34746
PGPEP1	-1.34438
COL8A1	-1.34364
MARCKS	-1.34149
CCDC30	-1.34083
HES2	-1.33953
SHC2	-1.33877
ATP8A1	-1.33728
FOS	-1.33673
ORAI3	-1.33348
FTH1	-1.32973
MAPT	-1.32864
PIANP	-1.32769
LOC10027074[illegible]	-1.3233
LOC388210,L[illegible]	-1.31926
HOTAIRM1	-1.31917
NRG2	-1.31801
BFSP1	-1.31749
C14orf159	-1.31732
ESR1	-1.31506

NOXA1	-1.31155
VDR	-1.30887
LMTK3	-1.30702
MST1	-1.30619
LYRM9	-1.30245
PLEKHA4	-1.30004
ACSF2	-1.29786
LOC10012882	-1.29781
LOC254896,TN	-1.29621
TSC22D3	-1.29371
PLA2G16	-1.28968
OR1F1	-1.28924
RNASET2	-1.28808
BCL6	-1.2861
PCDH7	-1.28571
LINC00472	-1.28521
CD70	-1.28503
GPR173	-1.27989
COMMD9	-1.27949
ABTB1	-1.27909
CCL28	-1.27887
CTSO	-1.27709
IFITM1,IFITM2	-1.27685
MMP13	-1.27208
LOC10028891	-1.26995
FOXO1	-1.26815
ARHGAP24	-1.26621
KIF17	-1.26597
HIST1H2AC	-1.26155
EGLN3	-1.25898
RASSF6	-1.25667
GATS	-1.25437
SLC16A3	-1.25425
MXRA5	-1.25401
TLCD2	-1.25306
EFNB3	-1.24899
CCNG2	-1.24881
GYG2	-1.24638
BATF2	-1.24317
MMP11	-1.24255

LOC100272217	-1.23774
STC1	-1.2364
ZNF114	-1.23378
LOC339535	-1.23229
YBX2	-1.23118
KLC4	-1.23111
CTXN1	-1.2291
LOC100507639	-1.22659
SYTL1	-1.22412
HNMT	-1.22214
LRG1	-1.22113
CCL20	-1.22109
SYT11	-1.21953
EFNA1	-1.21915
SOX12	-1.21847
RHBDL2	-1.21262
PDE3A	-1.21216
A4GALT	-1.21183
CALHM2	-1.2112
FUCA1	-1.20876
ZNF396	-1.20871
LINC00511	-1.20513
HBP1	-1.2045
ATP8B1	-1.20267
RND1	-1.20253
C19orf66	-1.20244
CNGA1	-1.20158
FOXO4	-1.19905
DENND1C	-1.19869
TFEB	-1.19808
IFIT3	-1.19787
TMEM92	-1.19729
LRRC17	-1.19567
KLHDC9	-1.1946
TRIM46	-1.19369
HIST2H2BE	-1.19318
FCGRT	-1.19312
ZNF852	-1.19163
LOC100132999	-1.1907
PLA2G4C	-1.18764

NCAM2	-1.18762
RAPGEF3	-1.18652
AK7	-1.18556
SNAI3-AS1	-1.18394
CLDN4	-1.18357
SLC18B1	-1.18099
PAPLN	-1.17909
NRP1	-1.1759
FAM131B	-1.17527
ACBD7	-1.17274
GPR56	-1.17165
ROPN1L	-1.17105
PDE9A	-1.17058
PTX3	-1.16951
C12orf52,TPCN	-1.16792
C1QL4	-1.16759
CXCL3	-1.16655
GRB7	-1.16554
PTGER2	-1.16454
PLAC8	-1.16427
TMEM129	-1.16361
ATP2A3	-1.16345
UBE2L6	-1.16281
FKBP7	-1.16259
TBKBP1	-1.16252
CRIP2	-1.16155
NCKAP5L	-1.16125
DDO	-1.16028
TMEM150A	-1.15797
PCDHB7	-1.15459
FADS2	-1.15447
PLVAP	-1.15386
KDM6B	-1.15336
ANKRD20A19F	-1.15306
OAS2	-1.15245
SLC25A27	-1.15229
BSPRY	-1.15084
SLC27A2	-1.15064
REEP2	-1.14873
GLT8D2	-1.14397

LOC10050812(	-1.14266
GRN	-1.13955
EGFEM1P	-1.13936
FLJ22447	-1.13862
NAT14	-1.13707
LINC00648	-1.13514
CES3	-1.13433
CADM1	-1.13282
EPCAM	-1.13227
VAV3	-1.13141
FKBP8	-1.13092
C12orf55,LOC1	-1.1308
LCN2	-1.13016
RHPN1	-1.12957
KLHL14	-1.12483
APOL1	-1.12003
SEMA3B	-1.11631
TLR5	-1.11599
BEX4	-1.11358
FAM19A5	-1.11327
B3GNT4	-1.11088
HIST3H2A	-1.11079
FOXQ1	-1.10844
SPATA17	-1.10752
C1R	-1.10681
C1R	-1.10681
PLCD3	-1.10676
P2RY6	-1.10624
CDKN1C	-1.10608
PTP4A3	-1.10296
IL8	-1.10232
IRF2BPL	-1.09999
TP53INP1	-1.09884
SPON1	-1.09803
PHLDA1	-1.09694
LOC10050740(	-1.09335
BNIP3L	-1.09279
OXLD1	-1.09242
FAM43A	-1.09094
PGAP3	-1.088

LOC10012978'	-1.08735
BCL3	-1.08619
LOC10050605	-1.08571
PAQR8	-1.085
C21orf119	-1.08491
C20orf96	-1.08027
LOC10012959	-1.07976
TOX2	-1.07974
ANG,RNASE4	-1.07971
FAM117A	-1.07905
BBC3	-1.07689
NPDC1	-1.07687
TNF	-1.07584
CYS1	-1.07538
KCNJ11	-1.074
HCFC1R1	-1.07264
ABCD1	-1.07163
CTSD	-1.07014
PCDHB4	-1.06982
GHDC	-1.06958
ARRDC1	-1.06917
HGD	-1.06879
PTGIS	-1.06816
LOC153684	-1.06643
LOC10050731	-1.06516
C15orf48	-1.06509
CCDC69	-1.06197
CXorf22,CXorf	-1.06126
GDPD1	-1.06075
SLC2A11	-1.06055
PCSK1N	-1.06035
THBS3	-1.06024
JUP	-1.05955
PRUNE2	-1.05808
L1CAM	-1.0568
RASL11A	-1.05516
LOC730102,SE	-1.05441
CCDC103	-1.05384
SLC44A3	-1.05282
KRT19	-1.05055

CARD6	-1.04755
IGFBP6	-1.04648
ARHGEF3	-1.04593
RTN4RL2	-1.04487
LRRC4B	-1.04485
GJD3	-1.04445
VMAC	-1.04411
GSTM4	-1.04355
FKBP1B	-1.04194
TSPAN33	-1.03596
NME3	-1.03409
SPP1	-1.03375
KLF9	-1.03296
LOC143666	-1.03242
WDR45	-1.03037
MTMR9LP	-1.03026
SMAD6	-1.0279
LOC441204	-1.02647
DHRS1	-1.02622
SLC26A4	-1.02454
SHF	-1.02384
DNER	-1.02042
SLPI	-1.01897
SEC31B	-1.01865
UPK3B	-1.0185
MCTP1	-1.01821
C14orf132	-1.01818
LINC00663	-1.01727
ACP6	-1.01667
SLC27A1	-1.01538
SCD5	-1.01471
FAM173A	-1.01354
MIR4680,PDCD	-1.01186
HCP5	-1.01063
PAOX	-1.01061
CACNA1H	-1.00986
SEZ6L2	-1.00907
KCNK5	-1.00741
LOC151009	-1.00713
FIG4	-1.00603

BHLHE41	-1.0026
TMEM159	-1.0025
LOC729013	-1.00233
LOC653160	-1.00155
TENM1	-1.0004
PLCD1	-0.999625
LLGL2	-0.998299
LPHN3	-0.996763
FBXL16	-0.993591
RRAGB	-0.991669
PI16	-0.991283
C20orf112	-0.991174
ICAM5	-0.99098
TMEM169	-0.990852
DBNDD1	-0.989503
ZNF579	-0.988241
SCG2	-0.98659
ZC3H12B	-0.986062
GAMT	-0.985138
FGFBP1	-0.983633
ZNF580,ZNF58	-0.983313
TCEA2	-0.981681
SEMA3A	-0.980781
LINC00534	-0.977829
CCNA1	-0.976953
FZD1	-0.976835
FAM85A,LOC1	-0.976336
DUSP6	-0.973368
INPP5J	-0.971195
LOC10028879	-0.971084
TFPI	-0.970126
FBP2	-0.96973
FZD2	-0.968556
NMUR2	-0.968165
PPP1R1B,STAI	-0.96765
SLC26A11	-0.965918
DPYD	-0.965258
TNFSF12,TNF	-0.964756
CPA4	-0.964236
TMEM121	-0.963394

ZSWIM5	-0.963087
ARSE	-0.960538
LOC10050705€	-0.960065
MOSPD3	-0.958376
NNMT	-0.958371
BEX2	-0.955937
AKR1B1	-0.955633
ZNF358	-0.955301
RINL	-0.953839
LOC440894	-0.953141
CHRD	-0.95236
ARRDC3	-0.952239
CYP39A1	-0.951744
CTSS	-0.950049
LONRF3	-0.949772
ROGDI	-0.947969
HS3ST1	-0.946349
SREBF1	-0.945817
SLFN5	-0.945246
MALL,NPHP1	-0.944902
DENND2D	-0.94393
THNSL2	-0.942888
PCOLCE	-0.942407
SALL2	-0.942075
EHF	-0.942049
DPM3	-0.942006
DPP4	-0.940819
MARCH9	-0.940313
KIAA0408,SOG	-0.939735
SUSD3	-0.939719
UBXN6	-0.938567
C17orf100	-0.937895
GPR153	-0.937624
MLXIPL	-0.936426
MYL6B	-0.936124
KCNC3	-0.93332
MOAP1	-0.931724
CCDC106	-0.931245
SLC7A7	-0.93111
KDM5B	-0.929991

MAP3K8	-0.929186
B4GALNT4	-0.928948
SLC25A23	-0.92845
SPAG4	-0.927672
UCP2	-0.920837
KLHL7-AS1	-0.920393
TM4SF1	-0.920293
FAM22A,FAM2	-0.919039
FRMD4B	-0.918204
EMID1	-0.917072
QPCT	-0.915463
BCAS3	-0.915273
CALCRL	-0.914878
SERPINI1	-0.914618
VWA5A	-0.914296
SCD	-0.91417
GUCY1B2	-0.911701
GBP1	-0.911497
VPS37D	-0.910611
GSE1	-0.909385
CXCL16	-0.909277
EPAS1	-0.908786
TMEM170B	-0.908158
ZNF219	-0.905954
SHISA4	-0.905621
ACVRL1	-0.905204
EFNB1	-0.90339
HOGA1	-0.902485
PIP5KL1	-0.902083
CAT	-0.901662
ECHDC2	-0.901257
REPS2	-0.900759
SLCO4A1	-0.900754
GSTT1	-0.898156
SLC29A2	-0.897707
FRAT1	-0.897004
RASGRP1	-0.895677
STEAP1	-0.893796
GMPR	-0.893518
PRDM8	-0.892082

PEMT	-0.892077
FN3K	-0.890181
NIPSNAP1	-0.88884
ZFPM2	-0.88856
GJB2	-0.888408
ZMYND10	-0.887995
FLYWCH2	-0.887608
TNNT1	-0.88738
DDAH2	-0.886909
FADS3	-0.88549
PTPN6	-0.884223
OSCP1	-0.882297
LOC728743	-0.882282
MIR4750,TBC1	-0.881914
TRAPPC9	-0.881052
RPH3AL	-0.880608
IFNGR1	-0.880261
NOVA2	-0.880053
FLJ39632	-0.878761
MKNK2	-0.877726
MIR920,SOX5	-0.877687
BTC	-0.877264
JUND	-0.876037
IFI16	-0.876002
IFITM3	-0.875401
RFXANK	-0.874962
ZBTB7C	-0.874467
PAQR6	-0.87433
SH3TC1	-0.872776
CEBPB	-0.872655
ZBTB7B	-0.872162
JUNB	-0.871335
MVP,PAGR1	-0.871229
FAM131C	-0.869795
HEXIM1	-0.867943
EPS8L2	-0.866505
PPARA	-0.864647
NR2F1	-0.863955
CUBN	-0.862776
SLC6A8	-0.862668

DTX1	-0.862092
KLHDC7A	-0.861906
SLAIN1	-0.859935
CAPG	-0.859177
PFKFB4	-0.859103
LYRM5	-0.858999
LOC10028815	-0.857712
PLA2R1	-0.857683
MIB2	-0.857485
ALDH6A1	-0.856259
NUPR1	-0.854955
LIPA	-0.8516
LOC10012909	-0.851538
PTPRH	-0.851026
FRK	-0.850702
WNT5A	-0.85013
ZMAT1	-0.848167
KLHDC1	-0.846363
MBTD1	-0.846069
MTHFR	-0.845311
NEAT1	-0.844004
NEIL1	-0.843859
NRBP2	-0.843573
SSH3	-0.841173
INO80B,INO80	-0.84108
TLE6	-0.840979
BCKDHA	-0.840718
MAP3K5	-0.840239
CITED2	-0.840104
CCDC85B	-0.839622
VAMP1	-0.839343
MFAP2	-0.838251
KCNRG,TRIM1	-0.837793
SNTB1	-0.835699
TMEM38A	-0.834574
GPR162	-0.834227
SPIN2B	-0.834046
TBC1D8B	-0.833432
TMEM53	-0.831897
CBLB	-0.830866

SNCA	-0.82897
NEU1	-0.827851
PRSS16	-0.82653
ACOT2	-0.825144
CES2	-0.825016
SGSH	-0.823439
YPEL5	-0.822966
UNC13A	-0.822358
CRYL1	-0.821917
PNPLA2	-0.818688
AHR	-0.818382
BCAM	-0.818109
NDRG4	-0.818035
WBSCR27	-0.81709
RAB3D,TMEM2	-0.81609
TMEM59L	-0.813766
IGFBP1	-0.813197
HOXB5,HOXB6	-0.813048
AXIN2	-0.812246
MAGEH1	-0.812126
FAM198B	-0.81089
LOC149351	-0.810109
HIBADH	-0.809994
C1RL-AS1,CLS	-0.809946
ZDHHC24	-0.809413
TPGS1	-0.809278
FBXO17	-0.808715
ZNF785	-0.808664
CAPN1	-0.808235
REPIN1	-0.80765
SERPINB1	-0.807212
FA2H	-0.806774
RN7SK	-0.805535
LAPTM5	-0.805197
PLD3	-0.801854
FOLR1	-0.801617
MAL2	-0.801536
DENND6B	-0.799937
RAB13	-0.799273
GSTA4	-0.798221

GLUD2	-0.79764
LAMB2	-0.797062
PWWP2B	-0.795219
STAT6	-0.791841
PEX11G	-0.790891
NMRK1	-0.790356
PDGFRL	-0.789881
C9orf16	-0.788711
ID4	-0.788596
TTYH3	-0.788451
C12orf68	-0.788347
SOX4	-0.787332
ING4	-0.787066
IGFBP7	-0.786744
SGK223	-0.786324
SGK2	-0.786267
MAATS1	-0.785901
LOC10050611[illegible]	-0.784105
HR	-0.783589
PHF21A	-0.783577
IGBP1	-0.783513
SOX17	-0.783147
PTTG1IP	-0.782624
GALT,IL11RA	-0.781758
MAP7	-0.781159
ASIC1	-0.779039
SLC35E2	-0.778172
BST1,FAM200[illegible]	-0.778081
FAM8A1	-0.777752
PNMA2	-0.776888
METTL7B	-0.771609
BAIAP3	-0.770944
WDSUB1	-0.769762
C6orf162	-0.769607
ADAMTSL4	-0.769526
ANKDD1A	-0.769397
SLC29A4	-0.769301
HOOK2	-0.767891
EPHX1	-0.767735
KIAA1551	-0.767276

DNASE2	-0.766752
HIST1H1C	-0.765298
GLRX	-0.764249
ARHGAP27	-0.76385
TMEM173	-0.762497
PTH2R	-0.762071
PLEKHA7	-0.762004
PCDHB5	-0.761889
SPRED3	-0.761447
CD9	-0.760818
RARG	-0.760425
SUMF1	-0.759843
PPP1R14C	-0.758281
NR2F6	-0.758252
MATN2	-0.758124
C1orf115	-0.757922
PROS1	-0.757749
PRRX2	-0.757041
YDJC	-0.755885
DUSP4	-0.755618
TSC22D1	-0.755254
RIMKLA	-0.75367
IDH1	-0.753644
ZFP36L2	-0.752925
ARHGEF4	-0.751955
AKD1	-0.751611
CTTNBP2	-0.750811
SYNGR1	-0.750571
MPND	-0.750157
ITGB6	-0.749778
PROC	-0.749543
ARID5B	-0.747755
LOC10050730[illegible]	-0.747066
EHD3	-0.746612
SPRY1	-0.745651
CMTM7	-0.744617
PDK2	-0.744058
PLD1	-0.743988
IRF7	-0.741858
TGFBR2	-0.74163

NRARP	-0.741181
BTN3A3	-0.740644
SLC38A2	-0.740595
SIGIRR	-0.738462
PLAG1	-0.736977
FBXL19	-0.736187
ARRB1	-0.73511
C16orf13	-0.73465
TRPT1	-0.734381
FAM117B	-0.733741
MR1	-0.732634
SPRY4	-0.731986
CXXC5	-0.731712
CDC42BPG	-0.731617
HEXIM2	-0.731204
ZNF517	-0.730974
BTBD2	-0.730429
KNDC1	-0.72885
MGAT3	-0.727146
PROSAPIP1	-0.726195
FAM84B	-0.725689
SCAND3	-0.725407
IFT27	-0.725113
HMG20B	-0.723836
OBSCN	-0.7229
CDC42EP4	-0.722887
PLAC2	-0.72262
ACOX2	-0.720477
PEX6	-0.719748
PLXNB1	-0.717891
MAGEB2	-0.717605
FAM214B	-0.716949
GSN	-0.716749
ID2	-0.716599
MIR3605,PHC2	-0.716219
ABHD14B	-0.716122
IL6	-0.715958
NREP	-0.715736
MSI1	-0.715109
TNFRSF1A	-0.714403

ITFG3	-0.714325
TSNARE1	-0.713037
RSG1	-0.712801
ALKBH7	-0.711954
TNS3	-0.71122
TOB1	-0.709943
TNFAIP3	-0.709142
CCDC146,DTX	-0.707908
ACADS	-0.707865
EXD3	-0.707623
TNIK	-0.707202
ID1	-0.705435
SH3BP2	-0.702104
RAB40B	-0.701717
BNIP3	-0.701536
TTC30A	-0.70057
MXI1	-0.700391
HLA-E	-0.700191
IDNK	-0.699471
LOC646778	-0.699383
TOM1	-0.698657
SYNPO	-0.698638
PHLDB3	-0.698473
ARHGEF16	-0.697619
PCMTD1	-0.696421
TLR3	-0.696199
GLTSCR2	-0.694237
MZF1	-0.694168
PSORS1C3	-0.693903
MNT	-0.692792
GPSM3,NOTCI	-0.690576
PTK2B	-0.68934
BBS1	-0.688985
SCAND1	-0.688335
PSMB9	-0.687867
TINAGL1	-0.687836
PSME1	-0.686933
ACTN3	-0.686669
BTN3A1	-0.686099
ST3GAL5	-0.685851

LPAR1	-0.685495
TMEM123	-0.685413
ANKRD5	-0.68508
PPM1H	-0.684612
AMN1	-0.682868
CTSA	-0.682646
SH3BGRL	-0.682218
RILPL2	-0.681591
TSPAN15	-0.681285
RAI2	-0.681221
BTN3A2	-0.680156
LOC550643	-0.679406
FUK	-0.677665
MPST	-0.676503
TIE1	-0.676217
ZNF608	-0.675311
NBR2	-0.67456
ANKRD34B	-0.673774
ICK	-0.673629
ICA1	-0.673607
PPP1R16A	-0.672489
GDPGP1,TTLL	-0.67248
HOXB3	-0.67232
CTDSP2	-0.67202
ZNF395	-0.671653
SLC52A3	-0.671515
FAM214A	-0.671317
C17orf59	-0.670802
MAGED2	-0.67072
HOXB7,HOXB8	-0.670249
SORCS2	-0.67023
IQSEC2	-0.670185
ZNF821	-0.66928
RSPH3	-0.664963
COL4A4	-0.664768
VAT1	-0.664123
TMEM67	-0.662825
METRNL	-0.661908
DNAJC4,NUDT	-0.661556
SYTL2	-0.661388

SPINT2	-0.66065
CHMP1B	-0.660545
C7orf60	-0.660523
LURAP1L	-0.66039
HDAC5	-0.660339
PCMTD2	-0.659349
MNX1	-0.658848
UNC93B1	-0.658794
ZBTB12	-0.658282
HKDC1	-0.657209
TMEM231	-0.656777
ARHGEF25,SL	-0.656706
FBXL15	-0.654394
CCDC15,SLC3	-0.654279
SNRPN,SNURI	-0.653787
PSTPIP2	-0.652924
NCOA7	-0.65153
WNT7B	-0.650936
PRAME	-0.650217
GRINA	-0.650019
PLCH1	-0.649774
ADAP1	-0.648406
LRRC27	-0.648099
ACSS1	-0.648052
SCN2A	-0.647812
LRCH2	-0.647519
UBE2H	-0.647474
MRAS	-0.647424
PTMS	-0.647125
C11orf9	-0.646997
THBD	-0.646732
SLC7A11	-0.645993
CHST2	-0.6458
CXCL1	-0.645556
SH3BP1	-0.645326
FAM100B	-0.645008
TUBA1A	-0.644089
SURF1	-0.643995
ABHD11	-0.643084
FGFRL1	-0.642467

KLHDC8B	-0.641821
MARCH2	-0.641557
PIGZ	-0.641457
PLK1S1	-0.641452
ARSD	-0.640726
GPX4	-0.640602
CUTA	-0.640411
SMIM3	-0.639793
MGST2	-0.639099
HAP1	-0.638951
ZNF350	-0.638435
MEGF6	-0.636042
KLHL13	-0.636018
KRCC1	-0.633928
PPAP2B	-0.633788
LMBRD1	-0.63343
STAT2	-0.633335
NAPRT1	-0.633164
LTBR	-0.632474
TRAPPC5	-0.631237
BCL2L11	-0.631216
ZFP2,ZNF454	-0.630781
OSBPL5	-0.630289
IFIH1	-0.629376
EBF4	-0.629007
PPP1R35	-0.628808
BCAN	-0.628577
BAIAP2-AS1	-0.628513
EPHA10	-0.625727
EPOR,RGL3	-0.625683
SEMA4G	-0.625676
CAV1	-0.624934
C1QL1	-0.624351
CMTM8	-0.62373
TRPC6	-0.62355
FBLN2	-0.622668
SLC35E2B	-0.622432
NFIL3	-0.622257
ZNF385A	-0.622113
ATP6V0E2	-0.62211

LRRN2	-0.621624
RASSF9	-0.621325
GPR108	-0.620823
TCTN1	-0.620567
ZBTB42	-0.619802
NFKBIA	-0.619352
SYNGR3	-0.619133
CUEDC2	-0.618829
FAM213A	-0.617862
OGFR	-0.617648
SORBS1	-0.616902
KCTD1	-0.616734
ZNF444	-0.616237
HAPLN4,TM6S	-0.614764
FGF11,TMEM1	-0.613189
ATF6B	-0.612973
ZSCAN30	-0.612874
KLHDC2	-0.612453
FDFT1	-0.611789
OAF	-0.611365
APOL6	-0.610231
CDK19	-0.609829
SLC4A11	-0.608509
GM2A	-0.608499
FAM3C	-0.607733
MAFF	-0.605678
CDC25B	-0.604535
SERINC5	-0.60426
PRKCDBP	-0.60411
CARD8	-0.603502
UGCG	-0.603462
NUCB1	-0.603253
C5orf55	-0.603184
SLCO2B1	-0.602632
KIAA1217	-0.60182
GTF2IRD2B	-0.600297
RNF44	-0.600246
BARX1	-0.599954
TPD52L1	-0.59977
IFT43	-0.599347

SLC25A42	-0.598874
TOM1L2	-0.59877
SPSB3	-0.597735
SLC9A7	-0.597535
ZNF628	-0.597107
CST3	-0.597053
JMY	-0.596546
UGDH-AS1	-0.596305
DTX4	-0.595447
SAMD12	-0.595374
SH2D3A	-0.594968
TP53INP2	-0.594591
SKAP2	-0.593441
YIPF3	-0.592039
FLOT2	-0.588785
ITM2B	-0.588686
GLB1L	-0.588466
GFPT2	-0.588118
MAF1	-0.587318
IFI6	-0.585635
PPAPDC2	-0.585014
VWA1	-0.58473
VEGFB	-0.584215
FAM86C2P	-0.583781
KIAA0040	-0.583087
VAV1	-0.582606
LRP10	-0.582508
HOXD9	-0.582394
MGST3	-0.582163
EGFL7	-0.58169
PLEKHA1	-0.581314
FAM127B	-0.580797
PIK3R1	-0.57994
ZFP14	-0.579409
CCDC149	-0.578872
TANC1	-0.578798
TMEM74B	-0.578295
FLJ20464	-0.577668
PRODH	-0.576748
HSD17B1	-0.576506

WDTC1	-0.575841
IRF1	-0.575647
TCF7L2	-0.57564
C14orf79	-0.575533
PPCS	-0.575369
ASAP3	-0.573794
ZNF747	-0.573608
SBK1	-0.57201
TMEM187	-0.571699
IP6K2	-0.571483
DIRAS1	-0.57134
BASP1	-0.571141
METTL23	-0.571133
SEMA4B	-0.57101
ETHE1	-0.57034
SPIRE2	-0.570175
MRC2	-0.569785
TRERF1	-0.569561
WWTR1	-0.569121
PML	-0.56856
CD40	-0.56753
TMCC3	-0.567213
PPP4R4	-0.564866
C1orf106	-0.56336
HTRA1	-0.562724
ANKRD29	-0.560721
LBH	-0.560651
STMN3	-0.560303
KCNJ16	-0.559156
ST6GALNAC4	-0.559004
PLEKHA5	-0.558952
ASB13	-0.558639
CYFIP2	-0.558603
NAGLU	-0.557492
PRAF2	-0.556915
RTN2	-0.555295
SPINT1	-0.554777
PGLS	-0.554123
HIST1H2BK	-0.554025
GABARAP	-0.553223

CTDSP1	-0.552734
CBS	-0.552428
HOXD8	-0.551392
CCDC24	-0.551361
MTUS1	-0.551336
MIR21	-0.550908
MMP15	-0.548639
CACFD1	-0.54716
MAT2B	-0.545635
TMEM59	-0.545473
NEDD4L	-0.543991
CSPG5	-0.541085
GRTP1	-0.540992
TRIM21	-0.540988
TMEM181	-0.540942
SQSTM1	-0.538988
FDXR	-0.538371
C1QTNF6	-0.538229
MEIS1	-0.537225
HOXA13	-0.537058
MIR3614,TRIM	-0.537023
FAM127A	-0.536781
RGL2	-0.536499
RRAGA	-0.536463
IL18R1	-0.535794
HOXB13	-0.535011
BBS2	-0.534959
CACNB3	-0.534613
EFNA3	-0.533934
IKBKE	-0.533829
AIG1	-0.533788
POLR3GL	-0.533086
CCDC92	-0.531789
DHX40	-0.531543
LOC728431	-0.531118
DNAL4	-0.530953
GARNL3	-0.530008
NCOA1	-0.529525
SLC35F3	-0.528776
FGF18	-0.527532

TMEM8B	-0.527086
FLI1	-0.525389
GSTK1	-0.525134
SNORA47,ZBE	-0.524641
EDN1	-0.524375
MYLIP	-0.523975
TUSC1	-0.523046
CNIH3	-0.522269
HPS1,MIR1287	-0.520763
MAPK3	-0.520654
BCAS1	-0.519761
MAP3K1	-0.519193
LOC93622	-0.518989
LOC388692	-0.518385
PTPRS	-0.518368
ZER1	-0.518219
CAPN5	-0.516835
DAZAP2	-0.516755
C10orf54	-0.516581
NFKBIZ,NXPE3	-0.516466
FIGF,PIR,PIR-I	-0.515646
SLC22A23	-0.515258
RNF5	-0.514715
PNP	-0.514469
RAB36	-0.513972
MPI	-0.51338
RPP25L	-0.513253
DRAM1	-0.51253
TRPS1	-0.512529
SQRDL	-0.511066
TXNIP	-0.509566
GJA3	-0.509323
FTL	-0.509309
MTSS1L	-0.509244
SATB1	-0.507656
GLRB	-0.506204
C17orf67	-0.506074
LAPTM4B	-0.505517
C1orf226,NOS1	-0.504483
MYADM	-0.503936

ZNF362	-0.502143
RBL2	-0.501982
PTGR1	-0.501612
FAM109A	-0.501427
HIP1R	-0.500907
UAP1L1	-0.50088
CHMP2A	-0.500143
EPB41L1	-0.499517
HS1BP3	-0.499423
CAMK2N1	-0.499417
H6PD	-0.49899
RABEP2	-0.498792
C9orf3	-0.497855
FAM108A1	-0.497709
APBB1	-0.497096
LSS	-0.497053
FOXE1	-0.496919
SAP30L	-0.495825
RTN4R	-0.494133
STXBP2	-0.493862
ECH1	-0.493856
TFDP2	-0.493699
FHL3	-0.492766
PSMB10	-0.492752
MAT2A	-0.492659
LZTFL1	-0.492551
OGDHL	-0.49194
CCDC120	-0.491579
TSC22D4	-0.491563
ARHGAP39	-0.489619
NADSYN1	-0.488577
PP7080	-0.487728
ETV5	-0.48765
SLC30A1	-0.487617
IRF2BP1	-0.486679
H2AFJ	-0.486332
PIH1D1	-0.486234
TPBG	-0.486067
NNT	-0.485571
NFATC1	-0.484473

SPECC1	-0.484407
GOLPH3L	-0.484189
CRHR1,CRHR	-0.482582
EIF4B	-0.482002
CYP2U1	-0.481529
AKNA	-0.48146
SPRY2	-0.481118
CYHR1	-0.481024
LINC00623	-0.480595
XKR6	-0.480332
CLIP2	-0.48009
ACAD10,ALDH	-0.479643
NGFRAP1	-0.479418
ZBED2	-0.478739
SHARPIN	-0.478404
TMEM219	-0.477641
MIR1469,NR2F	-0.477175
SLC25A29	-0.476439
C4orf3	-0.47632
LTA4H	-0.47561
WDR60	-0.474953
FBXW9	-0.474817
SIPA1	-0.473136
HERC6	-0.472853
TEAD2	-0.471936
TBC1D22A	-0.470838
OAZ2	-0.470655
IMP3	-0.470602
RABAC1	-0.46961
RNF150	-0.469397
QPCTL	-0.469209
HDAC11	-0.468488
KCTD18	-0.467561
IGSF8	-0.466852
RASGEF1A	-0.465855
MAPRE3	-0.465734
DAPK1	-0.464416
WTAP	-0.464332
ZNF486	-0.463825
HSD17B8	-0.463423

DENND3	-0.462206
NR1H2	-0.461741
C2orf29	-0.461698
ARHGEF17	-0.461391
USP30	-0.461321
HLA-F	-0.461111
SELO	-0.459917
FAM174B	-0.458846
GMCL1	-0.458604
CASC4	-0.457088
SLC1A3	-0.455845
FAM63B	-0.455468
SYNGR2	-0.455011
MAPK8IP3	-0.454684
HMGCL	-0.454111
ZSCAN18	-0.451928
SPPL3	-0.45152
ACER3	-0.450995
ARHGAP12	-0.450703
WDR54	-0.450517
ZFAND6	-0.449894
SERPINA1	-0.449552
GALNS	-0.44903
AHCYL1	-0.448672
VCAN	-0.446289
TSPYL4	-0.446044
PHPT1	-0.446033
CUEDC1	-0.445252
MRPL10,SCRN	-0.445059
DECR1	-0.444302
FAM171A2	-0.443918
SUDS3	-0.443459
LGALS3	-0.443435
MAP4K3	-0.443325
H1FX	-0.442289
SLC24A6	-0.442206
LONRF2	-0.442101
OS9	-0.441961
PON2	-0.440254
RASSF7	-0.439593

COL5A2	-0.439538
MARCKSL1	-0.439452
LGALS3BP	-0.43942
PARP14	-0.437388
ANXA1	-0.436058
DCAF5	-0.435881
PRDX5	-0.435667
C1orf233	-0.435335
RAB11B	-0.434629
GMPR2	-0.434469
FBRS	-0.434377
LIMCH1	-0.433793
GNE	-0.432537
SYT17	-0.431374
BIN1	-0.43044
IL18	-0.430302
SPNS1	-0.429797
CPNE1,RBM12	-0.429768
SLC25A1	-0.428478
FAM210B	-0.426845
CCDC6	-0.424748
IRAK2,VHL	-0.424521
CAMK2D	-0.423883
COA5	-0.423838
CLCN7	-0.423298
FAM160B1	-0.423168
KAZN	-0.423094
OTUD5	-0.422473
MINK1	-0.422438
SLC50A1	-0.421891
ZNF516	-0.420502
STX4	-0.420386
LGR4	-0.420092
VPS51	-0.419532
RB1CC1	-0.418453
PNPLA6	-0.418382
SPG21	-0.417939
MOV10	-0.417704
PDP1	-0.41608
TPRG1L	-0.414946

WDR59	-0.414927
CD302,LY75,LY	-0.414923
TMUB2	-0.414212
BTBD1	-0.41287
ARL6IP5	-0.410318
ADAM10	-0.409461
NRIP1	-0.409045
PCDHB2	-0.406923
BST2	-0.406639
HSPB1	-0.406573
CORO1B	-0.40617
H3F3AP4	-0.405464
SLC46A1	-0.403842
NBR1	-0.403483
TPRA1	-0.40291
RNF130	-0.402546
ARAP1	-0.402317
KLF13	-0.400674
PPP1R3B	-0.400576
GLIS3	-0.400427
C19orf43	-0.400076
SREBF2	-0.398882
ZBTB1	-0.398709
SALL1	-0.395961
CD81	-0.393792
GCC2	-0.392786
QARS	-0.389277
PPP1R9B	-0.385938
MIR639,TECR	-0.385579
PRKD2	-0.384519
ZNF217	-0.382864
TNFRSF21	-0.381629
APOL2	-0.380835
COASY	-0.37826
MAGED1	-0.376022
MLLT6	-0.375723
IER3	-0.373371
KCNK1	-0.370162

(J) Genes significantly upregulated by sh-TLE2

Gene list	log2-fold-change
FGF1	5.85538
NAV3	4.81581
SERPINB7	4.43693
MARCH4	4.05713
GPR111	3.46902
CGB8	3.45376
KCNH1	3.29653
SPHK1	3.29165
SERPINE1	3.27218
LOC10050742(	3.15456
MYH16	3.1516
ESM1	3.13118
STRA6	3.12677
HCG22	3.0774
TNC	2.76345
PDCD1LG2	2.687
EGOT	2.66607
CGB5	2.58213
LRRC38	2.55963
SEMA7A	2.53728
CCBE1	2.52144
GLIPR1	2.51643
LOC340113	2.50859
GABBR2	2.46632
GDF6	2.4452
TRIML2	2.35465
WDR69	2.34768
ANXA8L1,LOC(	2.26571
AOX1	2.26053
LMCD1	2.25629
PTHLH	2.20676
ADAMTS1	2.20269
P4HA3	2.18578
SCEL	2.14841
LAMC2	2.10629
NR6A1	2.09346
ADRA1B	2.08935
NEXN	2.07669
CREB5,LOC40	2.06309

FGF5	2.0116
ZNF185	1.98276
PDPN	1.97061
C1QTNF2	1.96628
CD274	1.93586
MGC4294	1.89304
MYO7B	1.87794
C3orf52	1.8611
FAM83A	1.83623
FRMD6	1.81071
PEAR1	1.79789
GALNTL4	1.79752
ALPL	1.75163
SPOCK1	1.74884
TGFBI	1.73675
MB21D2	1.73613
XDH	1.73471
BTBD11	1.71138
BCYRN1	1.65641
LRRN4	1.62909
THSD4	1.60289
CYP26B1	1.59401
HHIP	1.59294
CGB7,NTF4	1.57167
LOC10050637	1.55222
TLL2	1.5494
PLXNA2	1.52663
CYP4F11	1.5182
FERMT1	1.51378
FAM101B	1.50888
COL4A1	1.49894
MSC	1.49495
LAMA3	1.49436
E2F7	1.46547
ADAMTS5	1.46074
ZNF542	1.45591
NKX6-1	1.45485
DACT1	1.44638
INHBA	1.4351
LHFP	1.42102

CCDC80	1.41574
FSTL3	1.40951
CYP1A1	1.4041
CRYAB	1.39259
DOCK2	1.39222
GAL	1.38893
KBTBD8	1.3878
GPR19	1.38257
RFTN1	1.36023
GALNT6	1.35648
ANXA8L2	1.3555
SNAI2	1.35028
THBS1	1.34475
CYR61	1.33852
RBP1	1.32905
PHLDB2,PLCXI	1.32178
HBEGF	1.31708
S100A2	1.31051
KLF7	1.30791
ANPEP	1.29779
CLSPN	1.29387
NAV2	1.29239
CLCN5	1.29027
MICAL2	1.28507
OXCT1	1.26805
PSMD2	1.2657
C9orf47,S1PR3	1.2562
LOC401164	1.25456
TIMP3	1.24928
XYLT1	1.24741
NRIP3	1.23672
LRP4	1.23537
LIMA1	1.22498
COL1A1	1.20802
SACS	1.20698
B4GALNT1	1.20562
SGK1	1.20427
PTPRF	1.20023
PIEZO1	1.19691
TNS1	1.1965

HIC1	1.1912
GPR126	1.18338
CDK6	1.17749
WDR4	1.16363
MYH9	1.16095
STRIP2	1.15777
RN7SL2	1.1571
PDGFB	1.15474
FOSL1	1.1526
ADAM19	1.14926
TNFRSF12A	1.14734
UCN2	1.13891
ENTPD4,LOXL	1.13659
TRPM2	1.12797
MKI67	1.12454
CENPI	1.12421
UBASH3B	1.12088
JPH2	1.12007
LOC10050627	1.11732
ITGB1	1.11339
EFNB2	1.11217
APCDD1L-AS1	1.11161
PLAU	1.10937
CAPN2	1.10882
OXTR,RAD18	1.10593
DIAPH3	1.10455
TPM4	1.10276
SHANK3	1.10238
MAP1B	1.09921
PMEPA1	1.09899
ANXA3	1.09724
SKA1	1.09414
CTPS1	1.08785
LOC81691	1.07984
ANLN	1.07873
APBA1	1.07714
CKAP2L	1.07244
RPE65	1.07212
ZNF729	1.07094
MME	1.06854

CORO2B	1.06142
APOBEC3B	1.06123
CTGF	1.06096
COL5A1	1.0566
FANCI	1.0541
RASSF1	1.05338
TCF19	1.05326
NLRP3	1.05195
POLR3G	1.04723
KIAA1524	1.04406
EDNRA	1.04352
TTF2	1.04169
ABLIM3	1.04062
DOCK10	1.03959
PSMC3IP	1.03779
CENPK	1.03708
TRIP13	1.03477
ACE2	1.02982
ARHGAP31	1.02952
C3orf67	1.02904
SEMA6A	1.02557
OSBPL6	1.02531
MYOM3	1.02404
LTBP2	1.02238
WDR62	1.0154
MIR3939	1.01345
NTN1	1.01197
MYBL2	1.0097
NUP155	1.00743
SFN	1.00702
KNTC1	1.0064
HSP90AA1	1.00571
MYBL1	1.00432
POLQ	1.00402
CDH2	1.00222
UCA1	0.999642
FANCA	0.999356
UPP1	0.997331
DSEL	0.996726
SHCBP1	0.993496

CHST3	0.990792
MCM10	0.99048
FAM57B,LOC1	0.990133
WDR67	0.990024
MICA	0.989961
PLK4	0.984664
ENPP1	0.984484
PTPRR	0.979654
CCDC36	0.979547
KIT	0.978738
CYP27B1	0.977846
BAMBI	0.975673
PTPRB	0.974326
FAM196B	0.973979
MARCH3	0.973912
TLL1	0.973133
FKBP5,LOC28!	0.971602
KIAA1715	0.971093
GEM	0.970758
NFASC	0.969395
HOMER2	0.968887
C7orf53,IFRD1	0.968783
ESPL1	0.964985
PHF17	0.964404
MT2A	0.964248
PIGW	0.96409
MFAP5	0.962806
BARD1	0.962417
CASC5	0.95966
SLC6A17	0.957549
FAM72D	0.956304
ESCO2	0.955627
ZWINT	0.953073
KDR	0.952116
FLNB	0.951747
ETS2	0.950755
MID1	0.949706
TUBB2A	0.949
YOD1	0.946843
GSG2	0.946016

NCAPH	0.944468
LOC10050639[illegible]	0.94283
UTP20	0.942258
S100A3	0.940206
CDH18	0.938542
ADCY1	0.937236
CDCA5	0.936935
NCS1	0.93396
ERCC6L	0.933415
C6orf183,CEP[illegible]	0.930806
SBDSP1	0.929782
FANCB	0.929604
ARHGAP11A	0.929464
ZNF107	0.929164
RGS20	0.928804
SNHG1,SNOR[illegible]	0.926503
MTBP	0.924264
KATNAL1	0.923903
NUP188	0.923573
HSF2BP	0.922374
SCML2	0.922112
KIF18B	0.922081
CENPW	0.921166
ZNF367	0.920246
MAMLD1	0.919039
HRASLS	0.916636
GADD45B	0.915988
PRKAB2	0.915968
EXO1	0.915732
CDCA4	0.91335
CDC25A	0.911612
CHSY3	0.909431
HIVEP3	0.908211
UBE2T	0.90673
CENPO	0.900265
UNKL	0.89948
PARM1	0.898949
E2F8	0.898884
CCNA2	0.898096
CCNF	0.897716

C11orf82	0.897143
NCAPG	0.89641
BRCA2	0.896257
EFR3B	0.896245
PRKDC	0.895997
PDE4B	0.895727
PRPS1	0.895221
HS3ST3A1	0.894298
DCLRE1B	0.894057
ALMS1	0.892918
TUBA1B	0.891826
ACTN4	0.888873
PODXL	0.88884
TANC2	0.888753
SH3RF3	0.886899
IQGAP3	0.882309
CAD	0.882205
ZNF850	0.881487
STIL	0.879467
ARSB	0.879358
DDX21	0.878872
CAMK4	0.877838
BRIP1	0.876974
TICRR	0.876041
TUFT1	0.875011
TBX20	0.874828
NOL6	0.874773
ANKRD1	0.874769
SPDL1	0.874076
MYO19	0.873636
CENPN	0.871793
TRAF1	0.870157
SLC7A5	0.869414
CDH3	0.869117
ARNTL2,C12or	0.86884
ORC6	0.863777
RANGAP1	0.862994
OLR1	0.862671
SLC1A4	0.859127
ZNF365	0.858085

GTF2H2C	0.856421
PLCB4	0.856419
DLX1	0.853306
JAG1	0.851907
CYCS	0.850772
AURKB	0.850651
RTKN2	0.849573
COL7A1,MIR71	0.848177
HAUS2	0.847924
FAT1	0.847203
SAMD4A	0.846724
BLM	0.846495
CCNE2	0.84614
NID1	0.846128
ALS2CL	0.8446
DKK3	0.844482
KLF10	0.844197
CD83	0.844191
NCAPG2	0.843806
SPATA5	0.841554
COL4A2	0.841073
RACGAP1	0.84015
IFRD2	0.838424
PPCDC	0.838344
RAD51AP1	0.836746
MOXD1	0.836116
NEIL3	0.835939
BUB1B,PAK6	0.835583
ARHGAP33	0.834595
CDCA2	0.834399
MCM8	0.834265
POLE	0.834131
CDCA8	0.833888
RECQL4	0.833442
MEX3B	0.833179
TMEM74	0.831993
SKA3	0.831587
GFOD1	0.831551
CSTF2	0.83153
NXPH2	0.831173

ARHGAP19,AR	0.830892
KPNA2	0.830533
RIBC2	0.82825
HS3ST3B1	0.827354
STAC	0.827297
TGFB2	0.826847
C17orf53	0.824918
RBL1	0.824105
ATAD2	0.824073
PRC1	0.822782
MND1	0.819511
SMG9	0.818472
PPIF	0.818347
FXYD5	0.817788
MELK	0.816488
MICALCL	0.816043
ZNF643	0.815458
DGCR8,MIR13	0.81473
NFATC2IP	0.814525
ELFN2	0.813414
CDC45	0.812519
SHANK2	0.812433
TPM1	0.811652
CLTCL1	0.810645
C12orf75	0.810498
CALD1	0.810384
HIPK2	0.809444
STEAP3	0.808463
GADD45A	0.806702
CNTNAP1	0.806375
LINC00116	0.805977
LOC650368	0.805954
PBX3	0.803631
FABP5	0.803103
DPP9	0.801865
ZNF530	0.80117
SP6	0.800405
DHX33	0.799995
HJURP	0.79938
GINS1	0.798873

POLE2	0.798654
AFG3L1P	0.797779
C3orf14	0.797301
DNAJC9	0.797101
UTP15	0.796321
LSM11	0.796047
TNPO1	0.791973
LOC10050563[illegible]	0.791586
TMEM209	0.791419
STK39	0.791002
FGF2	0.790361
UHRF1	0.789734
PLP2	0.789585
PRSS23	0.789564
CEP128	0.789461
NRG1	0.789395
DCLK1	0.789253
FAM111A	0.786584
UGGT1	0.78573
LOC10019098[illegible]	0.783919
TTLL7	0.783708
SLC4A4	0.78271
DSN1	0.78264
FBXO5	0.782348
WHSC1	0.782323
LPAR5	0.781868
HSPH1	0.781813
TBC1D4	0.780972
RPS6KA4	0.780436
UBE2C	0.779731
CAP2	0.779725
GCLM	0.777531
DARS2	0.777474
GPCPD1	0.777371
GALNT14	0.777302
CDK1	0.776678
ASPH	0.775938
TOP2A	0.775153
EMG1	0.774842
MAST2	0.773719

C1orf116	0.773654
SUV39H1	0.773439
TRABD2A	0.773423
PSMC3	0.772731
PGM2L1	0.772204
GALNT7	0.77208
MTHFD2	0.77182
WDR77	0.771812
TAGLN2	0.770887
RBMX	0.770052
LPAR3	0.769752
SSR3	0.769506
ITGA2	0.769459
ARL14EPL	0.768843
KLHL18	0.768097
TMEM194A	0.767906
PLCE1	0.767698
ZSCAN12	0.767096
VCL	0.766226
TMEM18	0.766162
KIF4A	0.765308
MTOR	0.764707
GOSR2	0.763855
PCOLCE2	0.763427
LMF2	0.763359
KIF1C	0.763106
CASZ1	0.763101
LOC10013289	0.762859
DNMT1	0.762456
CCP110	0.762077
TMEM79	0.761896
URB2	0.759853
P4HA2	0.759585
PRSS3	0.759538
FLNA	0.759301
NDC80	0.758625
CALU	0.758562
KRT7,LINC005	0.758495
PA2G4	0.757996
CACHD1	0.757356

CCND1	0.75703
ATG4A	0.7567
ALDH1L2	0.75628
STX11	0.755123
CSNK1G1,KIA/	0.754803
DPH3	0.75374
PRIMA1	0.752274
CEP152	0.75175
F3	0.751428
SDC1	0.751265
PNPT1	0.751105
CDKN1A	0.751086
KLHL21	0.750825
TUBB6	0.750143
NUP205	0.74985
MMS22L	0.749796
PEA15	0.749679
CDC6	0.749058
WNT9A	0.748776
TPX2	0.748055
MIR548U	0.748027
PTDSS1	0.747846
JUN	0.747806
GTF2H2B	0.746134
CERK	0.746113
SPATS2L	0.74599
PLXNA1	0.745664
SRPX	0.745381
METTL4	0.745079
ATAD3A,ATAD	0.744758
LRRC59	0.744589
FRS2	0.744573
PARP2	0.744509
HPRT1	0.744347
ETS1	0.744317
FANCG	0.744012
C11orf91,CD59	0.743907
DUSP7	0.743615
C12orf5	0.74207
NUP50	0.741635

TERT	0.741087
FBXO45	0.740905
SYNJ1	0.7399
CENPJ	0.739625
PAK1IP1	0.738542
ITGA6	0.738317
ZNF30	0.738106
WDHD1	0.737946
EIF2B3	0.73704
SIK1	0.73693
ZNF142	0.736765
FANCD2	0.736295
LOC642846	0.736122
FAM54A	0.735839
SMC2	0.735317
SMC4	0.735306
PINX1	0.735287
FER	0.734931
SH3KBP1	0.734703
NOL9	0.734676
BRCA1	0.733205
MICALL1	0.732744
ARHGEF26	0.731967
MTMR9	0.731577
BHLHE40	0.73042
SMG1	0.72989
RRM2	0.729375
RFK	0.729334
LRR1	0.728682
GXYLT1	0.728663
RPL26L1	0.727957
MYEOV	0.727595
MAP3K14	0.727293
FIGNL1	0.727292
ITGA4	0.727042
NBPF24	0.726728
GRAMD1B,LO(	0.725739
CEP55	0.725369
MICB	0.725172
HECW2	0.72406

C12orf4	0.723908
FREM2	0.723359
DMBX1	0.720755
XPO4	0.720715
TLN1	0.72064
TIPIN	0.719198
SLC5A6	0.718478
TMEM98	0.718161
KIF11	0.717906
FANCM	0.717725
GLI2	0.717635
ZNF442	0.717599
VARS	0.716682
CD109	0.715864
POPDC3	0.715087
CD82	0.71473
CS	0.714364
IL32	0.714044
MIS18A,URB1	0.713427
LAMB3,MIR426	0.713336
CBFB	0.712887
HSPB8	0.712815
C1orf112	0.712701
FAM208B	0.712512
GXYLT2	0.712335
ACLY	0.711956
FBN1	0.711829
CEP85	0.711737
UBR4	0.711718
UBA6	0.711688
RANBP1	0.711447
PSMD11	0.711274
PLGRKT	0.711265
RTTN	0.708953
GPATCH4	0.708399
ALYREF	0.70789
BCL2	0.707068
PDCD11	0.706684
AKAP12	0.706401
GTSE1	0.705629

ATAD5	0.705573
DTYMK	0.705332
SYTL3	0.704815
TOMM34	0.704744
TFPI2	0.704086
PFKFB2	0.70351
TRAK2	0.702941
FGD6	0.702448
LAMB1	0.702075
DKK1	0.701672
ASF1B	0.701463
KCTD9	0.701146
SPSB4	0.700194
FHL2	0.699941
ZNF569	0.699151
USP31	0.699101
PBK	0.698474
PSMD1	0.697487
SNAI1	0.696472
FSD1L	0.695302
MAGOHB	0.694982
HAUS8	0.694922
MASTL	0.694916
TNFRSF9	0.694746
MRPL52	0.694409
LMNB2	0.693888
XRCC2	0.69342
MYO10	0.692557
ADRBK2	0.691793
UNC13D	0.691674
SLC27A4	0.691546
EZH2	0.69147
TRHDE	0.691012
LRRC3,LRRC3	0.69048
YWHAH	0.690433
PALLD	0.690371
POLR3K	0.689174
PKMYT1	0.688897
MSANTD3,MS/	0.68761
CMSS1	0.687461

XPO5	0.687372
KDELC2	0.687168
TRIO	0.686935
RAP1GAP	0.686819
IRAK1,MIR718	0.686815
STAG1	0.686135
TEX30	0.685869
C14orf105	0.685419
S100A10	0.685379
TUBGCP3	0.68521
AEN	0.685064
TSPAN5	0.684639
RUVBL1	0.684506
ALDH1B1	0.684125
BUB1	0.684117
PHF5A	0.684069
SIRPA	0.684039
ANKRD44	0.683976
CACNA2D1	0.683519
ADTRP	0.68171
CYP24A1	0.681256
WNK2	0.681123
ANO4	0.680873
LOC90784,POI	0.680676
MNS1	0.679994
EHBP1	0.679867
ZNF473	0.679742
HNRNPH3	0.679047
KIAA1549	0.678974
WDR76	0.678079
TMEM106C	0.67802
UAP1	0.677863
AXL,HNRNPUL	0.677736
RAB27A	0.676522
MIR1292,NOP5	0.675428
CCT5	0.674608
LIN54	0.674257
EHD4	0.67402
NKD1	0.673615
MRPL24	0.673569

EPT1	0.672745
SLC7A6	0.671189
MAPK11	0.670294
SRXN1	0.670098
CAMKK1	0.669842
FERMT2	0.669832
GABPB1	0.669318
SPAG5	0.66754
TIMM10	0.666227
GPR63	0.666202
MRTO4	0.665861
RPL39	0.665807
EFHD2	0.665394
TUBA1C	0.663617
SPARC	0.663212
AP1M2	0.663173
KIF2C	0.663047
CIRH1A	0.66194
PLOD3	0.661502
C15orf23	0.661384
GEN1	0.66111
SNHG15	0.66091
KIF23,PAQR5	0.660368
GJB3	0.66009
NCAPD3	0.65967
CORO1C	0.658852
RELT	0.658835
PRR11	0.658423
USP13	0.65828
POP1	0.658158
MMP16	0.657553
HCFC1	0.65713
ABL2	0.657021
MDC1	0.65697
SCFD2	0.656864
ICMT	0.656824
RGPD8	0.656601
ZMIZ1	0.655905
ERCC4	0.655624
NAA15	0.655179

PRDM2	0.655139
LOC440288	0.65484
C18orf54	0.654713
FAM72B	0.654441
POLR3A	0.654155
AP4E1	0.653996
SRM	0.6539
ZNF26	0.653496
KIF21A	0.653416
NAV1	0.653128
PTAR1	0.65279
FAM105A	0.652382
CRIM1	0.65229
SH3RF1	0.652265
SEPT11	0.65185
TNFRSF19	0.651789
AUNIP	0.650953
CNN3	0.650597
DTL	0.650005
KPNB1	0.649614
GPX8	0.649411
STARD13	0.64909
TMEM87A	0.649032
EMC1	0.648794
ELF4	0.647522
FAM177A1	0.647224
TTL	0.646787
RAP1GDS1	0.646585
VGLL3	0.646288
MARS	0.646008
TYMS	0.64575
BOP1	0.645521
FAM86JP	0.645279
OTUD6B	0.645199
LOC10028731[illegible]	0.64479
ZFAND2A	0.644532
MED27	0.644196
CDC20	0.644023
MSN	0.643905
METTL21D	0.643878

NOM1	0.643872
FZD8	0.643752
SLC36A1	0.643097
ENTPD5	0.642653
CDKN2D	0.642248
THAP10	0.642088
TTPAL	0.641572
CLPB	0.641336
FAM72A	0.641327
PRPF8	0.640366
P2RX5,P2RX5-	0.640221
NUPL1	0.640072
C18orf25	0.639795
SYNJ2	0.63931
SLX4	0.63878
WBSCR22	0.638483
GPR137C	0.638381
FUBP1	0.637808
POLA1	0.637327
MARK1	0.637116
RRP1B	0.636818
PASK	0.636631
ADAM23	0.636591
MAD2L1	0.636182
MPHOSPH9	0.635815
ASAP1	0.635468
GPR89A	0.634937
DOT1L	0.634772
NCAPD2	0.634648
EIF3C	0.634604
MPHOSPH6	0.633635
CEP78	0.633117
EML1	0.633031
RUNX1	0.631722
TUBG1	0.631673
BTG3	0.631379
FBXO43	0.630804
ERVMER34-1	0.630674
IPO5	0.630623
HEATR8,HEAT	0.629731

TRAPPC10	0.629575
TACC3	0.629179
TYRO3	0.629137
MAPRE1	0.627786
CENPF	0.627141
AMPH	0.62677
PRIM1	0.626454
MLF1IP	0.626223
HSPA8	0.626108
PCNT	0.626083
MAN2A1	0.625254
IRX5	0.625149
POLR3F	0.625112
INPP4B	0.625107
NOTCH2	0.624953
PIGA	0.624217
ABCE1	0.624188
DNA2	0.624186
KIAA1731	0.624054
ATP10D	0.623845
RFWD3	0.623633
ZNF844	0.623517
PEAK1	0.623111
AKAP2,PALM2,	0.622796
ROCK2	0.622523
IFFO2	0.621746
PTN	0.62137
KLF6	0.61955
NT5E	0.619487
FAM167A	0.619237
VRK1	0.619192
PHF19	0.618903
C3orf80	0.618691
MTHFD1L	0.618585
LOC595101	0.618385
MOB3B	0.618197
FARSB	0.617557
OCIAD2	0.617359
ORC3	0.61708
HAUS7	0.616136

CCND3	0.615208
MDM1	0.615011
PSMD12	0.614189
ACTN1	0.614022
TUBB	0.614002
GPD2	0.613587
SYDE2	0.613464
HNRNPA3,MIR	0.613195
GFM1,LOC100	0.613027
N4BP2	0.612965
RHOC	0.611971
FAM178A	0.611863
TOMM40	0.611819
FOXM1	0.611279
GEMIN5	0.6112
MLL2	0.610643
XPO6	0.610529
PSMG3	0.610222
AAK1	0.61018
HECTD2	0.61017
RCC1,SNHG3	0.609812
NABP1	0.609465
ST7L	0.609399
MCAM	0.609221
ADAT1	0.609194
EPG5	0.608995
PFDN4	0.608697
TPM3	0.6085
IDH3A	0.608492
GREB1L	0.608447
HSPA4L	0.608153
TOPBP1	0.608113
SKP2	0.606798
CLMP	0.606541
FOXJ2,NECAP	0.605876
HMGXB4	0.605695
NF2	0.60557
STC2	0.605488
ZNF583	0.605328
RAB31	0.604846

PAWR	0.604534
GDAP1	0.604352
PLEC	0.604107
RASAL2	0.603747
LHFPL2	0.602523
GNB4	0.60177
FAM111B	0.601046
TMX3	0.600879
DDX39A	0.600852
PVR	0.600789
HERC1	0.600353
C17orf85	0.600147
NUP85	0.600064
PRICKLE2	0.599338
NUP107	0.599189
TMOD2	0.598555
SENP1	0.598423
FANCC	0.598416
CEP85L	0.598238
ANKLE2	0.597818
KIF20B	0.597789
GMNN	0.597603
NDUFAF2	0.597582
MICALL2	0.597279
CKS2	0.597201
NEDD4	0.596597
SLC35D1	0.596112
CDC7	0.595891
ASCC3	0.595735
WDR1	0.595644
SMURF2	0.595447
ARID5A	0.595055
PDLIM3	0.594886
DCLK2	0.594875
ATP1B3	0.594631
HELLS	0.594058
SUSD5	0.594052
C16orf59	0.593509
STT3A	0.593268
SERTAD4	0.593165

UFD1L	0.592839
C11orf24	0.592247
LSG1	0.59203
NOLC1,PPRC1	0.591482
TAP2	0.591356
HS6ST2	0.590262
DPYSL5	0.589747
LINC00346	0.589661
EPHB2	0.589509
DDAH1	0.589448
RRP15	0.588621
BMP2K	0.588494
ERGIC2	0.588127
CCT6A	0.58793
HDAC9	0.587908
GLT25D1	0.587434
BCAP29	0.587412
ASPM	0.587189
DCLRE1C	0.587168
LHX1	0.586616
NUP214	0.58639
BRIX1	0.585964
F2RL1	0.585728
CSE1L	0.585554
TCP1	0.585087
PFAS	0.585078
KIAA0020	0.58499
NELL2	0.58448
MTHFD1	0.583871
ITPR2	0.583119
GRB10	0.58239
NIP7	0.582085
PTPRD	0.582011
BIRC5	0.581998
DCP2	0.581749
MRPS11	0.581471
MTX3	0.58135
HMGB1	0.580844
KRT80	0.580812
USP43	0.580675

MIR4687,RRM1	0.580656
FAM102A	0.580646
FLVCR1	0.580634
GPR4	0.580365
SGOL2	0.580317
NAA25	0.580286
C6orf228	0.580084
LINC00152	0.579411
CKAP5	0.57927
PSMB2	0.57822
LIFR	0.578157
HSPD1	0.577254
LAS1L	0.577184
BDP1	0.57675
ATF4	0.575812
FAM126B	0.575634
LOC10021600[illegible]	0.575603
ACVR1	0.575061
ITGAV	0.574912
BEND3	0.574877
IPO7	0.574336
GALNT2	0.574248
COTL1	0.574019
MYO5B	0.573962
PROSER1	0.573798
ABCB10	0.573782
HMGB2	0.573483
BMPR1B	0.573441
TSHZ3	0.573189
CBL	0.5728
ZNHIT6	0.572774
LTV1	0.572665
SNRPE	0.572364
KLF4	0.571732
ERI2	0.571713
GINS4	0.57128
HNRNPL	0.571034
IQCJ,IQCJ-SC	0.570612
WDFY2	0.570244
ARID3B	0.570027

UBR7	0.57
TFRC	0.569067
EXO5	0.568901
PLAGL2	0.568379
MED14	0.567684
CSRP2	0.567506
GPR180	0.567314
ZCCHC2	0.567202
TMEM104	0.566934
CYP27C1	0.566321
POLE3	0.566249
SASS6	0.566132
LOC10029414[illegible]	0.565915
C17orf51	0.565352
IARS	0.565163
SNRPG	0.564269
EIF2C2	0.563255
PPARGC1B	0.563145
PCSK5	0.563077
NLE1	0.562662
RRP12	0.561603
EMP2	0.561568
CTNNAL1	0.561374
ALKBH8	0.561126
STIP1	0.560677
ZW10	0.560198
OAS3	0.560099
DDX11	0.560031
TMTC3	0.559982
CDR2	0.559942
RPL22L1	0.559855
FGFR1OP	0.559711
EIF4E	0.559623
PLAA	0.559084
MT1X	0.558966
BZW1	0.558816
DKC1	0.558502
LZIC	0.557978
ODC1	0.55774
ARMC4	0.557677

IGF2R	0.5571
CTSL2	0.556818
RHOB	0.556734
KIFC1,PHF1	0.556393
HN1	0.556287
PHLPP2	0.555123
QTRTD1	0.554308
TOP3A	0.553954
POLD3,RNF16	0.553681
MICAL3	0.553517
IGHMBP2	0.553144
BCLAF1	0.552914
TMEM48	0.552647
SBDS	0.55258
TIMM21	0.55256
ABHD5	0.552189
NHEJ1	0.551977
EIF4G2	0.551802
KIAA1462	0.551487
MIR621,SLC25	0.551189
CHAF1B	0.551013
OTUD3	0.550507
RFT1	0.550503
ZNF81	0.550225
ARHGAP10	0.549884
SNRNP40	0.549845
PITRM1	0.54912
HSPA12A	0.548767
CCDC58	0.54875
PDLIM7	0.548732
HIGD1A	0.54861
NCAPH2	0.547742
RASSF8	0.547737
NOP2	0.54753
AGAP1	0.547167
PGBD1	0.547013
ARSJ	0.546951
MGC27345,RB	0.546663
LRRC20	0.546301
KIF22	0.546192

SGOL1	0.545604
SAMD4B	0.545504
DHODH	0.545331
PRRC2C	0.544726
TFDP1	0.544439
SLC35B1	0.544026
ZAK	0.543734
SEMA4F	0.543548
LRRK1	0.543473
CHEK1	0.543414
C2orf69	0.542972
SMC6	0.54241
PHTF2	0.542271
FAM83D	0.542223
SSX2IP	0.541949
RBBP5	0.541356
LETM2	0.541155
AATF	0.541026
IPO13	0.540948
GPC1	0.540302
NOL12	0.540014
GCN1L1	0.539911
SPAG9	0.53958
CDK5RAP2	0.53956
MNF1	0.539244
MATN3	0.538775
RMI1	0.538534
NUP153	0.538104
SP2	0.537851
ARL13B	0.537181
CALML4,CLN6	0.53705
SOGA2	0.53705
GEMIN6	0.536812
PNN	0.536112
DLC1	0.535843
DZIP1L	0.535801
MMGT1	0.535741
XPOT	0.53558
TTK	0.535555
MAP3K4	0.535514

CIT,MIR1178	0.535378
RECQL	0.534896
ZFP28	0.534831
MAPKAP1	0.534311
TGM2	0.534257
ITPRIPL2	0.534199
PRADC1	0.534056
ELOVL1	0.534007
ALDH1A3	0.533959
ZNF347	0.533901
ACAP3	0.533705
CKS1B	0.532984
GNPNAT1	0.532634
GJA1	0.532099
SFMBT1	0.531944
HHAT	0.531845
LOC10050612	0.531817
POLR2A	0.531721
NUF2	0.531428
SRPRB	0.531293
DCBLD2	0.53109
KCMF1	0.531013
PVRL1	0.530836
JAK2	0.530819
NPLOC4	0.530276
LARP4	0.530079
LIN9	0.529986
TPRKB	0.529973
HIATL1	0.529926
RB1	0.529152
TEX261	0.528926
MZT1	0.528865
SCML1	0.52857
WDR5	0.528141
TMEM38B	0.528004
ZNF441	0.527769
FAM65A	0.527712
BRI3BP	0.527668
CEP97	0.527105
RFC3	0.526352

FSTL1,MIR198	0.526132
MGST1	0.52607
SRPR	0.526006
UTP11L	0.525999
SIPA1L1	0.525976
LYSMD4	0.525826
DHDDS	0.525754
NCBP1	0.525517
RAN	0.525498
HIRIP3	0.524993
USP36	0.524545
ANXA5	0.52428
ABHD3	0.523735
EIF4G1	0.523439
ANAPC7	0.52266
BRPF1	0.522179
ZWILCH	0.521871
TNRC6C	0.521143
RAD54L	0.520615
DOCK5	0.520272
ENTPD1	0.520094
HMMR	0.519907
ANP32E	0.519525
SGMS1	0.519257
C22orf25	0.519026
PRPF4	0.518691
NDST1	0.518534
MYBBP1A	0.518216
HERC2	0.517976
CDON	0.517928
CALM2	0.517682
TCERG1	0.517673
AURKA	0.517603
CEP72	0.517093
LIN52	0.516507
PLEKHA6	0.516312
TBP	0.515809
ADRM1	0.515432
SUCO	0.515185
HEG1	0.514842

CENPM	0.514833
POC1A	0.514676
SMS	0.514262
AFAP1L1	0.514133
SETD8	0.514054
STRA13	0.513509
ARHGEF10	0.513451
INPP5A	0.51333
ZFP30	0.512537
STAM	0.512509
NEURL1B	0.512442
ENDOD1	0.5118
U2SURP	0.511719
TMEM206	0.511567
SUPT16H	0.51148
WEE1	0.511251
FAM126A	0.510664
ACTL6A	0.510602
DPH2	0.510403
FEM1C	0.510007
CDCA3	0.50933
ATG16L1	0.509257
B4GALT2	0.509046
BNIP1	0.508897
DNAJC10	0.50875
GNL2	0.508714
BVES	0.508629
PER3	0.50836
UACA	0.508168
TUBB4B	0.507946
EPHA2	0.50768
MBOAT2	0.507634
RAD23B	0.507379
PAICS	0.507225
PDCD5	0.507191
SOAT1	0.506775
SON	0.50661
CTDSPL2	0.506411
AP1S2	0.505831
NRF1,RNA5SP	0.505378

MIR1825,POFU	0.505125
COPS3	0.505063
HDGFRP3	0.504954
SLC25A22	0.504798
TTLL4	0.504771
ZNF585A	0.504722
C10orf47	0.504689
NR2C1	0.504588
NUP35	0.504557
LPP	0.504414
LRRC14	0.504245
RABGGTB	0.503958
ATXN7	0.50369
SNRPA1	0.503653
MIP,TIMELESS	0.503652
NDOR1	0.502819
C1orf151-NBL1	0.502546
LCAT,SLC12A4	0.502102
ARHGAP29	0.501931
KLHL20	0.501584
KLHL15	0.501198
NUP93	0.500726
TMEM54	0.500411
RANBP2	0.500222
FAM98A	0.49956
FEN1	0.499505
VBP1	0.499484
PVRL3	0.499344
TMEM208	0.498734
NCL	0.498353
TIMM17A	0.498329
TARDBP	0.498104
RPL21	0.497871
DSCC1,TAF2	0.497857
CCDC88A	0.497848
NOMO1	0.497431
SMG6	0.497179
KIF14	0.496851
AADAT	0.496307
C20orf72	0.495933

LOC388796	0.495519
HNRNPAB	0.495372
ZNF280C	0.495344
CEBPG	0.495335
NVL	0.495113
TNPO2	0.494531
FUT4,PIWIL4	0.494404
ZNF271	0.494238
HAUS3,POLN	0.494144
H2AFZ	0.494134
NID2	0.49379
C5orf30	0.493748
RRP7A,RRP7B	0.493564
ABCF2	0.493475
TRIM16	0.493266
DVL1	0.493253
RNF168	0.493164
NCEH1	0.492809
TAF9B	0.49278
MTFP1	0.492674
PRPS2	0.492468
TPR	0.492318
SMC3	0.492102
PTBP1	0.491848
TACC1	0.491845
LARS2	0.491815
GRWD1	0.49164
MED20	0.49137
JMJD6	0.490763
FAM58A	0.490454
BRCC3	0.490432
KIF5B	0.48984
EIF1AX	0.489787
USP1	0.489559
NR1D2	0.488911
BAZ1A	0.48876
LYAR	0.487611
RNASEH1	0.487162
PDPK1	0.487025
TMED5	0.486888

SLC38A9	0.486741
ABHD2	0.486571
PSMD6	0.486429
RIF1	0.486408
SPECC1L	0.486146
ZNF280B	0.486022
PPTC7	0.48596
NEDD1	0.485508
C9orf91	0.485114
C5orf34	0.484825
PTPLB	0.484613
JOSD1	0.484526
C1orf109	0.484438
ITFG2	0.484408
ADPGK	0.484123
METTL1	0.48336
PSMD7	0.483276
CDK8	0.482881
HNRPDL	0.482584
DENND1B	0.482126
SZT2	0.481855
WDR36	0.481795
GGCT	0.481695
DIXDC1,DLAT	0.481639
LSM6	0.481579
LOC10099662	0.481452
NPNT	0.481265
MBOAT1	0.481057
FSBP,RAD54B	0.481052
ATAD2B	0.480568
SMCHD1	0.480326
HTT	0.480222
PTPDC1	0.480076
TMEM97	0.479926
PSME3	0.479924
IPO9	0.479675
LRP12	0.479441
TBC1D2B	0.479321
FAM35A	0.479156
DCK	0.478378

GOLT1B	0.477947
PYCR1	0.477689
INTS6	0.477267
SF3B3	0.477069
MARVELD2	0.476991
SMPD4	0.476919
WDR44	0.476718
EPB41L2	0.476599
GTF3C6	0.476587
HEATR1	0.476094
SF3A3	0.476081
OPA1	0.475811
SNRPD1	0.475619
HSPA9	0.475379
NSFL1C	0.475326
GRPEL1	0.475301
RPP14	0.475141
CDC42SE1	0.474686
TRAIP	0.474204
SMAD9	0.47397
ZNF699	0.473719
ERO1L	0.473547
GLI3	0.472966
PLK2	0.472862
RAB23	0.47277
ZNF256	0.472534
EIF5A2	0.471585
SEMA3C	0.471116
USP47	0.470912
CPSF2	0.470912
UGGT2	0.470662
IQGAP1,ZNF7	0.47053
MED13	0.470213
VAPB	0.470147
CEP135	0.469355
ZNF823	0.469302
TMEM201	0.46893
COPS8	0.468762
GINS2	0.468688
LARS	0.468649

COL27A1	0.468235
CCDC14	0.468225
DIP2B	0.467853
FH	0.467802
CLDND1	0.467497
CHN1	0.46683
GRPEL2	0.466772
PLAT	0.466457
SEPT10	0.466373
HAT1	0.466111
DGKZ	0.466073
XPO1	0.466061
DICER1	0.465875
PRKCE	0.465709
TRIM24	0.465045
TTC7B	0.464692
ITPRIP	0.464591
ABTB2	0.463974
AHCTF1	0.463941
ZNF678	0.463802
CEP350	0.463762
KPNA3	0.463711
ARHGAP21	0.463563
H2AFY	0.462172
TWF1	0.462107
GBF1	0.462004
C19orf48	0.461588
SLC19A1	0.461374
CLOCK	0.461374
TOMM5	0.461109
DENR	0.460972
RPS6KA3	0.460809
SLC4A7	0.460652
NUP98	0.460517
YKT6	0.460454
NUDT19	0.460394
NIN	0.459869
APMAP	0.45934
PDRG1	0.459202
SNX10	0.45911

CCNE1	0.458878
CCT2	0.458802
THAP9-AS1	0.458756
USP45	0.458726
L3MBTL2	0.458681
SFR1	0.45859
B3GALTL	0.458587
CENPA	0.457907
SLC25A25	0.457764
PRKAR2A	0.457643
COL17A1,MIR9	0.45721
TNPO3	0.456912
VANGL1	0.456617
LDLRAD3	0.456324
SNX18	0.456068
C10orf2	0.456023
EIF5	0.455756
OGFOD1	0.455316
ARAP2	0.455227
SLC8A1	0.455148
FUNDC2	0.45504
MGAT5	0.454993
DCTN5,PLK1	0.454138
CPOX	0.453779
FRAS1	0.453385
MPDU1	0.453357
ASH2L	0.453294
CDH24	0.453142
MRPL18	0.453024
RNASEH2A	0.451938
NAT10	0.45178
FAM82A2	0.451745
CEP170	0.451671
DLGAP5	0.451125
BLZF1	0.451062
CREBBP	0.451021
CCDC86	0.450875
DHFR	0.450602
C8orf33	0.450554
SNRNP27	0.450278

SLCO1B3,SLC(	0.450034
C1orf212	0.44946
KIAA0664	0.448942
CLASP2	0.448198
RRS1	0.447958
DHX9	0.447934
GPAM	0.447715
PIGK	0.447595
GSTCD	0.447531
EPDR1	0.447439
RPS26	0.447419
MCM2	0.44736
ZBTB2	0.447281
B3GNT5	0.447204
TXNRD1	0.447136
FBF1	0.446502
TGOLN2	0.446056
DPYSL3	0.445546
RAB3B	0.44511
DLST	0.444809
TXN	0.444242
SLAIN2	0.444124
DESI2	0.444
MFN2	0.443279
KIAA0922	0.443211
WWC3	0.443053
CEP250	0.442802
TDG	0.442786
ITGA5	0.442674
MARS2	0.442591
ANKRD33B	0.442231
ACP1	0.441906
DDX51	0.441824
GALNT10	0.441806
DENND4B	0.441734
NR3C1	0.441595
LRRC8C	0.441454
SEC61G	0.441442
RAPGEF2	0.441199
DLG5	0.440624

FLNC	0.440552
CCDC34	0.440454
PFDN1	0.440319
AGPS	0.439618
ATR	0.439422
TK1	0.439407
MLL	0.439367
NRP2	0.439354
TTC13	0.439169
NAA50	0.439033
NPM1	0.438526
TUB	0.438387
TMEM51	0.437795
FOXN2	0.437381
TRMT6	0.437345
MED15	0.437129
DNAJB2	0.436798
MIR3658,UCK2	0.436546
CHD7	0.435843
FAM171A1	0.435732
TMCC1	0.435402
NBPF14	0.435048
LARP4B	0.435011
EIF5A	0.434888
MCM3AP	0.434541
MESDC1	0.434198
BZW2	0.434133
SF3A1	0.433983
RBX1	0.433883
TMEM2	0.433414
USP38	0.43341
MTMR2	0.433216
EXTL3	0.433191
UPF1	0.433162
GCNT2	0.432771
PDS5B	0.432175
DNAJC21	0.432144
RAPH1	0.432094
PTPN11	0.431989
ADAMTS15	0.431707

AP3M1	0.431525
DYRK1A	0.431496
CCSAP	0.431302
HN1L	0.431261
FGFR1	0.43114
FAM219A	0.430387
PGP	0.428895
PDS5A	0.428879
PPWD1	0.428809
NDUFA12	0.428783
POLR2K	0.428272
GAS6	0.428039
FKBP4	0.428004
GATAD2A	0.427619
ATL2	0.427563
AMOTL1	0.426897
ZFX	0.426733
ATP13A3	0.42673
RNF138	0.426555
NANP	0.42643
MAP3K2	0.426313
MAPK6	0.426204
LRRC8A	0.426084
DDX55	0.425613
BYSL	0.425609
DEPDC1B	0.425525
PANX1	0.425193
CDK5RAP1	0.424727
HBS1L	0.424674
PSMC6	0.423917
SELT	0.42375
PAQR3	0.423554
MCM4	0.423525
ABR	0.423499
SH3PXD2A	0.423205
GTF2A2	0.423118
ZBTB40	0.423068
PTTG1	0.422969
RHEB	0.422808
CHID1	0.422758

SPCS3	0.422501
CACYBP	0.422369
PIGU	0.421649
ISOC1	0.421648
TEX2	0.421395
DENND6A	0.420994
HSPA1A	0.420866
DIEXF	0.420719
SLC35F2	0.42044
NCLN	0.419623
PTPRK	0.419554
TPD52L2	0.418995
HRH1	0.418909
IQCB1	0.418881
UHRF1BP1L	0.418643
CHD9	0.418642
NSF	0.418407
KIAA0284	0.418343
PPP4R1	0.418311
ARMC1	0.417975
B4GALT4	0.417931
ZC3H13	0.417713
PHIP	0.417649
PEX26	0.41738
DAZAP1	0.417341
RC3H2	0.416668
RAB22A	0.416295
TUBGCP5	0.416064
SIVA1	0.415994
ASL	0.415382
FIGN	0.415361
USP16	0.414409
UBE3C	0.413728
ATP13A2	0.413375
PSMD14	0.413072
STAM2	0.412625
GTPBP1	0.41244
LNPEP	0.412421
TRIM59	0.412278
IER3IP1	0.412126

LUZP1	0.412107
IGF2BP1	0.412062
DRG1	0.411997
SENP2	0.411849
CNNM4	0.411768
TLN2	0.41152
KIAA0368	0.411253
DOCK7	0.411066
ELAC2	0.410924
MTR	0.41091
MIR632,ZNF20	0.410206
ATP6V0B	0.410058
CBX5,MIR3198	0.409892
MIS18BP1	0.409501
BUB3	0.40931
GTPBP4	0.409259
FNIP2	0.408787
CDT1	0.408757
HECTD1	0.40856
CAP1	0.408388
AGPAT9	0.407628
DUS1L	0.407288
ANKRD13A	0.407116
NELF	0.406716
CD44	0.406684
CCDC93	0.406578
TRRAP	0.40589
GLRX3	0.405707
FAM123B	0.405565
RPL32,SNORA	0.405561
DENND5A	0.405316
RPL36	0.405032
TSR1	0.405008
EML4	0.404774
IDE	0.404612
UPF3B	0.404595
AARSD1,PTGE	0.404463
WDR43	0.404075
PTPLAD1	0.403884
TAF5L	0.403557

LRRC58	0.403489
NAA10	0.402581
ARHGEF11	0.402444
ARFGAP1	0.402226
UHMK1	0.402155
QSOX2	0.402071
TMX1	0.401886
FRYL	0.401826
GOT2	0.401758
SVIL	0.400729
TMED8	0.400672
RPIA	0.400568
NDUFA6	0.400519
JARID2	0.400275
GDI1	0.400238
HSPG2	0.3997
PPP1R12A	0.399132
NBPF10	0.397905
TJP1	0.397898
VPRBP	0.39766
MAPKBP1,MGA	0.396375
SLC30A6	0.396242
UBQLN1	0.395806
MRPL3	0.395657
AHSA1	0.395394
MRPS10	0.395278
TIMM50	0.39521
MDFIC	0.394988
TMEM64	0.39438
RPTOR	0.394112
EIF4EBP1	0.393467
PRDX3	0.393187
PPP1R14B	0.393149
CHD1	0.392636
BRPF3	0.392514
C5orf22	0.392341
ZNF281	0.392286
USP37	0.391775
SPEN	0.391524
SMNDC1	0.390504

RALGAPB	0.390051
MTIF2	0.389721
WIPF1	0.389661
CNTF,ZFP91,Z	0.389559
MRPS16	0.389009
CCT8	0.38856
MBNL1	0.388209
BMS1	0.38819
PM20D2	0.387963
ZFR	0.387597
MRPL37	0.387458
PSMA1	0.387068
SUZ12	0.387054
CDK12	0.38693
ZNF268	0.38684
WDR75	0.386375
GGH	0.386271
PKIA	0.386263
RPL7L1	0.38597
CLPTM1L	0.385709
IPO4	0.385495
ERLIN1	0.385037
SNX5	0.384705
CHST11	0.384652
UBN1	0.384628
LETM1	0.384231
CDC27	0.383948
PCF11	0.383383
ZDHHC5	0.382993
NSUN2	0.382851
ASCC2	0.382783
CCT3	0.38277
VOPP1	0.382542
CAMSAP2	0.382487
GLE1	0.381491
SGCB	0.381444
NBN	0.381064
OTUD7B	0.379297
ASXL2	0.379244
SAP130	0.378994

PDE12	0.378426
CHTF18	0.378057
AMMECR1L	0.377879
DDX50	0.377416
OTUD4	0.377368
CPSF7	0.377164
CTNNBL1	0.376025
U2AF2	0.375139
USP12	0.374934
TMED9	0.37377
E2F1	0.373058
DNAJA3	0.37205
EPRS	0.371386
GYS1	0.371109
SLC29A1	0.358972

(K) Genes significantly downregulated by sh-ZNF498

Gene list	log2-fold-change
HIST1H2AM	-2.16694
SHISA3	-2.05237
RN7SK	-2.01549
CHCHD3	-1.91745
CXCR4	-1.69744
ANKRD1	-1.59602
ANGPTL4	-1.58732
RN7SL1	-1.33026
MAGEB2	-1.26996
NTSR1	-1.21184
RASEF	-1.17438
GBP1	-1.16422
DCAF12L1	-1.15836
CREBL2	-1.14416
ZNF204P	-1.0992
FKBP7	-1.09365
TM4SF18	-1.08337
IL1R2	-1.06574
FOS	-1.06105
LINC00669	-1.04156
ARHGAP24	-0.961295
EGR1	-0.950549

PHLDA2	-0.92178
SLC47A1	-0.902915
ETV1	-0.881212
SNHG10	-0.879757
SYK	-0.87451
EGLN3	-0.865182
STC1	-0.852568
KCNS3	-0.846397
EMP2	-0.846199
TEX19	-0.843125
SYT7	-0.837023
MED29	-0.825075
CAMK2B	-0.823695
SPP1	-0.822315
MAL	-0.812043
PAIP2B	-0.802957
C14orf132	-0.796236
NRGN	-0.783537
CADM1	-0.78352
SEC11C	-0.775276
ECSCR	-0.741465
POLR2L	-0.733148
PNMT	-0.732519
HEG1	-0.732338
TTC9C	-0.726298
LCN2	-0.719659
AP1S3	-0.711726
KIAA1737	-0.708209
SLPI	-0.698145
G0S2	-0.696503
ANO6	-0.692066
FOSL1	-0.674565
PRPF4	-0.662694
COX8A	-0.656529
MMP1	-0.6513
SLC25A24	-0.650715
SETD7	-0.639381
DCAF11	-0.636699
EPS8	-0.627233
ABCA3	-0.603971

MXRA5	-0.600632
LSR	-0.599152
PPIA	-0.593158
COX5B	-0.592055
ANXA4	-0.574611
EPAS1	-0.562457
MIR3610,RAD2	-0.552964
MIF	-0.527916
SUPT16H	-0.525799

(L) Genes significantly upregulated by sh-ZNF498

Gene list	log2-fold-change
COL6A3	2.77051
KCNJ15	2.399
BGN	2.37943
TIE1	2.20542
LOC10050742(	2.03697
GNGT2	1.79258
COL5A1	1.68537
GDF6	1.64616
FOXS1	1.64002
SCEL	1.56914
CYP24A1	1.5257
MAP7D2	1.49449
WDR69	1.47076
OSCAR	1.45889
MCAM	1.41312
LOC10050637	1.39354
COL1A1	1.38455
RASGRP3	1.3818
KRT81	1.37802
MYO7B	1.37137
SLC22A2	1.36651
PI16	1.32759
SYT11	1.31392
TLL2	1.29443
GPR17	1.27762
ANXA8L1,LOC(	1.27716
EFEMP1	1.25891
ZNF365	1.23297

SERPINE1	1.22874
AQP3	1.2215
ACTBL2	1.22045
FAM198B	1.20148
LPAR5	1.16814
DOCK2	1.15713
HHIP	1.14513
HS3ST3B1	1.14499
PLVAP	1.11854
SIK1	1.11086
AOX1	1.10356
SPOCK1	1.0927
GPR132	1.08908
ADAMTS5	1.07681
RBP1	1.07667
HECW1	1.06787
DCLK2	1.06751
PPARGC1A	1.05282
KRT17	1.04449
NOTCH3	1.02057
PTPRR	1.01797
NMRK2	1.00848
PLCB2	1.00835
UNC13D	1.00485
LINC00319	0.988494
FAM83A	0.984955
MMP13	0.971286
SLIT3	0.963049
ATP10A	0.95897
C1orf116	0.95752
PPYR1	0.948986
SLCO2B1	0.942544
FBN1	0.932593
AMTN	0.911167
LMCD1	0.909057
NKAIN4	0.901061
MATN3	0.869076
HSPG2	0.8681
MMP2	0.865732
SERPINB5	0.865418

DCHS1	0.860875
CCDC80	0.860532
MYOM3	0.852049
CPAMD8	0.851207
SYTL1	0.839517
PRUNE2	0.831227
HPGD	0.823411
ALS2CL	0.79454
LRP1	0.784859
MAP1A	0.77249
ADTRP	0.768929
SEMA7A	0.764794
ANPEP	0.758536
TGFBI	0.751422
ZNF853	0.750765
N4BP2	0.742642
DKK1	0.736297
CLDN16	0.735118
AHNAK2	0.726242
PGM2L1	0.717799
MMRN2	0.717363
BMF	0.691095
IGDCC4	0.690101
ITGA4	0.683153
COL4A2	0.682978
RASSF2	0.674403
TLN2	0.670978
TNS1	0.663364
MAP2	0.658048
THBS1	0.655575
ARHGEF40	0.651883
ABLIM3	0.649594
NID1	0.648765
TANC2	0.648097
TGM2	0.637647
DKK3	0.637596
NAV1	0.623766
CAMKK1	0.619115
MSMO1	0.594457
NDST1	0.59336

CAPN5	0.576521
BMP1	0.574716
GSN	0.570054
SACS	0.558792
CLIP4	0.552412
PLXND1	0.545515
HMGCS1	0.543246
MICAL2	0.534226
AKAP12	0.530934

Supplementary Table S3; GSEA analysis about TGF−β, Wnt and Notch pathway
Orange shaddow indicates ES positive and FDR q val < 0.25.
Light greeen shaddow indicates ES negative and FDR q val < 0.25.

a) Analysis about TGF−β pathway.

	MSL3		ZNF691		VPS45	
NAME	ES	FDR q−val	ES	FDR q−val	ES	FDR q−val
BIOCARTA_TGFB_PATHWAY	0.5403811	0.27684477	0.477841	0.72657055	−0.22978772	0.9995102
COULOUARN_TEMPORAL_TGFB1_SIGNATURE_UP	0.3615901	0.32034245	−0.33831945	0.96670735	0.27652997	0.67668533
GSE39820_CTRL_VS_TGFBETA1_IL6_CD4_TCELL_DN	0.30992135	0.44028	−0.27730408	0.8447982	−0.3285302	0.50310874
GSE39820_CTRL_VS_TGFBETA1_IL6_IL23A_CD4_TCELL_DN	0.32284978	0.3868936	−0.31539893	0.9189054	−0.22362721	1
GSE39820_CTRL_VS_TGFBETA3_IL6_CD4_TCELL_UP	−0.26472372	1	−0.23603275	0.97765905	0.27740258	0.6680788
GSE39820_CTRL_VS_TGFBETA3_IL6_IL23A_CD4_TCELL_UP	−0.30995017	1	−0.20855479	1	0.3504764	0.43230367
GSE39820_TGFBETA1_IL6_VS_TGFBETA1_IL6_IL23A_TREATED_CD4_TCELL_UP	0.40121773	0.085057825	0.21942785	1	−0.20159996	1
GSE39820_TGFBETA1_VS_TGFBETA3_IN_IL6_IL23A_TREATED_CD4_TCELL_UP	0.27733058	0.64752054	−0.28941548	0.8443031	0.27025402	0.6085698
GSE39820_TGFBETA1_VS_TGFBETA3_IN_IL6_TREATED_CD4_TCELL_UP	0.3094584	0.43635628	0.3344197	0.84029394	0.28738606	0.69111854
GSE39820_TGFBETA3_IL6_VS_TGFBETA3_IL6_IL23A_TREATED_CD4_TCELL_UP	0.33542925	0.3217461	−0.28736314	0.8834223	−0.3110829	0.6088518
GSE7460_CTRL_VS_TGFB_TREATED_ACT_CD8_TCELL_UP	0.3582939	0.20473683	−0.24276055	0.96914697	0.27951345	0.6511039
GSE7460_CTRL_VS_TGFB_TREATED_ACT_FOXP3_HET_TCONV_UP	−0.26859745	1	0.24731466	1	0.3113479	0.44940132
GSE7460_CTRL_VS_TGFB_TREATED_ACT_FOXP3_MUT_TCONV_UP	0.31357145	0.42904115	0.24479547	1	−0.34051073	0.547993
GSE7460_CTRL_VS_TGFB_TREATED_ACT_TCONV_UP	0.2702862	0.6862889	−0.2915909	0.8811646	−0.24313158	0.94089633
GSE7460_CTRL_VS_TGFB_TREATED_ACT_TREG_UP	−0.23057917	0.94030744	0.22740935	1	0.3589767	0.8185762
GSE7460_FOXP3_MUT_VS_WT_ACT_WITH_TGFB_TCONV_UP	0.2656412	0.691557	−0.35949907	0.694746	−0.27796456	0.82515514
GSE7460_TREG_VS_TCONV_ACT_WITH_TGFB_UP	−0.23324488	1	−0.3232621	1	−0.2512833	0.9600649
GSE7460_WT_VS_FOXP3_HET_ACT_WITH_TGFB_TCONV_UP	0.36845735	0.19962642	−0.284465	0.8542935	−0.28789485	0.79496974
HALLMARK_TGF_BETA_SIGNALING	0.5689209	0.011948913	0.3137297	1	−0.35475713	0.64365995
JAZAG_TGFB1_SIGNALING_UP	−0.2455987	0.89352214	0.27858344	1	0.36946192	0.34684515
JAZAG_TGFB1_SIGNALING_VIA_SMAD4_UP	0.37647521	0.26503608	0.26604137	1	−0.3369867	0.6172237
KARAKAS_TGFB1_SIGNALING	0.6831599	0.030115526	0.39972362	1	0.4139961	0.64510536
KARLSSON_TGFB1_TARGETS_UP	0.34089252	0.37636	0.22460715	1	0.3462242	0.4669323
KEGG_TGF_BETA_SIGNALING_PATHWAY	0.5402862	0.014567942	0.37790832	0.97032684	−0.4087919	0.36949003
LABBE_TARGETS_OF_TGFB1_AND_WNT3A_UP	0.4382159	0.07989972	−0.43711746	0.33164203	−0.45161828	0.10913716
LABBE_TGFB1_TARGETS_UP	0.3547772	0.3511142	−0.33854786	0.8153721	−0.36835358	0.52013934
MCBRYAN_PUBERTAL_TGFB1_TARGETS_UP	0.4408747	0.0507683	0.2812999	0.99237025	−0.34967306	0.54542977
PID_TGFBR_PATHWAY	0.55508965	0.022571694	0.390575	0.6208283	0.2572577	0.9277079
PLASARI_TGFB1_SIGNALING_VIA_NFIC_10HR_UP	0.3021211	0.76678586	−0.40769216	0.9942093	−0.4284113	0.4634889
PLASARI_TGFB1_SIGNALING_VIA_NFIC_1HR_UP	0.5095106	0.12169546	0.5175032	0.5452426	−0.53674126	0.13614236
PLASARI_TGFB1_TARGETS_10HR_UP	0.44301108	0.03098119	−0.46609718	0.12422489	−0.47141936	0.030542627
PLASARI_TGFB1_TARGETS_1HR_UP	0.4215751	0.36143988	−0.5522924	0.22344385	−0.6597626	0.015069796
REACTOME_DOWNREGULATION_OF_TGF_BETA_RECEPTOR_SIGNALING	0.4073083	0.6340184	−0.27393168	0.9914002	0.41068345	0.6402698
REACTOME_SIGNALING_BY_TGF_BETA_RECEPTOR_COMPLEX	0.32258034	0.6779398	−0.2891549	0.9509344	0.35954398	0.500554
REACTOME_TGF_BETA_RECEPTOR_SIGNALING_ACTIVATES_SMADS	0.42707115	0.4869164	0.27592072	1	0.39654353	0.5923773
TGFB_UP.V1_UP	0.42301112	0.04976303	−0.294912	0.8568111	−0.35369998	0.47671142
TRANSFORMING_GROWTH_FACTOR_BETA_RECEPTOR_SIGNALING_PATHWAY	0.5248387	0.08648107	0.30031395	1	−0.4323284	0.55539924
VERRECCHIA_DELAYED_RESPONSE_TO_TGFB1	0.53273493	0.058297176	0.3468315	1	−0.47941568	0.301777
VERRECCHIA_EARLY_RESPONSE_TO_TGFB1	0.680585	0	0.3653835	0.6790151	−0.46614	0.27915487
VERRECCHIA_RESPONSE_TO_TGFB1_C1	0.65099365	0.027478533	0.28340515	0.98314774	−0.37731293	0.9431669
VERRECCHIA_RESPONSE_TO_TGFB1_C2	0.6977464	0.010430511	0.45462587	0.69029784	−0.62633795	0.087673545

VERRECCHIA_RESPONSE_TO_TGFB1_C5	0.47096065	0.36216813	0.39869124	1	−0.4671716	0.48957118

	ITGB3BP		TLE2		ZNF498	
NAME	ES	FDR q−val	ES	FDR q−val	ES	FDR q−val
BIOCARTA_TGFB_PATHWAY	0.3634821	0.97032285	0.3076893	0.9821319	0.6819432	0.05775441
COULOUARN_TEMPORAL_TGFB1_SIGNATURE_UP	−0.3294671	0.64927965	0.31608036	0.55799353	0.4652232	0.09590269
GSE39820_CTRL_VS_TGFBETA1_IL6_CD4_TCELL_UP	0.25801522	0.95647043	0.33983153	0.2996092	−0.28432623	0.7718209
GSE39820_CTRL_VS_TGFBETA1_IL6_IL23A_CD4_TCELL_UP	0.292941	0.8255734	0.47263452	0.017686283	0.32437637	0.42068172
GSE39820_CTRL_VS_TGFBETA3_IL6_CD4_TCELL_UP	−0.3185336	0.82406175	0.46404117	0.01659165	−0.3416211	0.57380825
GSE39820_CTRL_VS_TGFBETA3_IL6_IL23A_CD4_TCELL_UP	0.32210213	0.62935543	0.5343883	5.00E−04	0.25923178	0.8512007
GSE39820_TGFBETA1_IL6_VS_TGFBETA1_IL6_IL23A_TREATED_CD4_TCELL_UP	0.24454567	0.9676859	0.33144516	0.34533182	0.37981126	0.15502831
GSE39820_TGFBETA1_VS_TGFBETA3_IN_IL6_IL23A_TREATED_CD4_TCELL_UP	−0.24598199	0.9852032	0.28149623	0.5647332	−0.2555241	0.917931
GSE39820_TGFBETA1_VS_TGFBETA3_IN_IL6_TREATED_CD4_TCELL_UP	0.213852	1	0.43592358	0.034901656	0.42810524	0.08614361
GSE39820_TGFBETA3_IL6_VS_TGFBETA3_IL6_IL23A_TREATED_CD4_TCELL_UP	0.3237918	0.65904444	0.33362776	0.35282886	0.36047873	0.19789575
GSE7460_CTRL_VS_TGFB_TREATED_ACT_CD8_TCELL_UP	−0.36676624	0.6344128	0.31455034	0.46008578	−0.322133	0.58842826
GSE7460_CTRL_VS_TGFB_TREATED_ACT_FOXP3_HET_TCONV_UP	−0.31016278	0.6816038	0.30922383	0.4738329	0.24007162	0.9116174
GSE7460_CTRL_VS_TGFB_TREATED_ACT_FOXP3_MUT_TCONV_UP	0.25961122	0.98178965	−0.33996123	0.6843642	0.305904	0.50106984
GSE7460_CTRL_VS_TGFB_TREATED_ACT_TCONV_UP	0.34813124	0.66560674	−0.24823833	1	0.26975438	0.76914936
GSE7460_CTRL_VS_TGFB_TREATED_ACT_TREG_UP	−0.32161823	0.8131395	0.55373436	0	−0.24065024	0.9708549
GSE7460_FOXP3_MUT_VS_WT_ACT_WITH_TGFB_TCONV_UP	−0.2544812	0.9530237	0.3412039	0.31689003	0.26701543	0.80276597
GSE7460_TREG_VS_TCONV_ACT_WITH_TGFB_UP	0.36865616	0.5848241	0.30801085	0.5100858	0.3113688	0.49870822
GSE7460_WT_VS_FOXP3_HET_ACT_WITH_TGFB_TCONV_UP	0.3498785	0.5756246	−0.33336717	0.6615155	0.28175047	0.6712838
HALLMARK_TGF_BETA_SIGNALING	−0.4117195	0.641973	0.32887632	0.58483905	0.5447834	0.058139697
JAZAG_TGFB1_SIGNALING_UP	−0.28349963	0.9343974	0.30801827	0.56399053	−0.2852892	0.88177514
JAZAG_TGFB1_SIGNALING_VIA_SMAD4_UP	−0.30443823	0.94596034	−0.36042345	0.656004	−0.37233016	0.59089905
KARAKAS_TGFB1_SIGNALING	−0.47396865	0.6890506	0.43466264	0.57321614	0.623503	0.11126467
KARLSSON_TGFB1_TARGETS_UP	−0.4227017	1	0.66039264	0	0.3428562	0.43332914
KEGG_TGF_BETA_SIGNALING_PATHWAY	0.37419665	0.6188694	−0.35591006	0.72949165	0.33452576	0.50426424
LABBE_TARGETS_OF_TGFB1_AND_WNT3A_UP	0.3360171	0.6841063	−0.33232063	0.7588593	0.44040895	0.087277785
LABBE_TGFB1_TARGETS_UP	0.4061855	0.60446846	−0.3342732	0.7785406	−0.30784485	0.74184275
MCBRYAN_PUBERTAL_TGFB1_TARGETS_UP	0.29370376	0.8032177	0.34772587	0.32136998	0.37845734	0.16579674
PID_TGFBR_PATHWAY	−0.27132845	0.9813654	−0.26327148	0.98243815	0.4684817	0.13417935
PLASARI_TGFB1_SIGNALING_VIA_NFIC_10HR_UP	0.51319534	0.61817986	−0.3469239	0.8435724	0.343913	0.6714268
PLASARI_TGFB1_SIGNALING_VIA_NFIC_1HR_UP	0.52053183	0.47474772	−0.37658462	0.8290475	0.53078496	0.113472074
PLASARI_TGFB1_TARGETS_10HR_UP	0.36331707	0.5584864	0.41230324	0.052621383	0.37998188	0.14497031
PLASARI_TGFB1_TARGETS_1HR_UP	−0.43555084	0.66914535	0.3678949	0.5815973	−0.35824394	0.7319573
REACTOME_DOWNREGULATION_OF_TGF_BETA_RECEPTOR_SIGNALING	−0.4485863	0.6269628	0.42296308	0.5996832	0.30755353	0.9804657
REACTOME_SIGNALING_BY_TGF_BETA_RECEPTOR_COMPLEX	−0.37480965	0.7314499	0.37056383	0.4460543	0.40275094	0.26420766
REACTOME_TGF_BETA_RECEPTOR_SIGNALING_ACTIVATES_SMADS	−0.41608867	0.78579754	0.43724316	0.5903038	0.36458406	0.8662023
TGFB_UP.V1_UP	0.27387977	0.86086863	0.45138234	0.02333683	0.46689788	0.054184854
TRANSFORMING_GROWTH_FACTOR_BETA_RECEPTOR_SIGNALING_PATHWAY	−0.26756954	0.98312867	−0.29615748	0.9955149	0.44353488	0.2601834
VERRECCHIA_DELAYED_RESPONSE_TO_TGFB1	−0.29438713	0.99316424	−0.34848958	0.8396581	0.51661384	0.10322032
VERRECCHIA_EARLY_RESPONSE_TO_TGFB1	0.3212285	0.8898317	0.33978623	0.58131456	0.60393035	0.032853488
VERRECCHIA_RESPONSE_TO_TGFB1_C1	−0.3870836	0.9670639	−0.3522351	0.9907619	0.6903008	0.07760037
VERRECCHIA_RESPONSE_TO_TGFB1_C2	0.42676243	0.7853702	0.4130634	0.5761999	0.585087	0.09673534
VERRECCHIA_RESPONSE_TO_TGFB1_C5	−0.4070195	0.9782493	−0.39387766	0.85692155	0.59533983	0.09301672

b) Analysis about Wnt pathway.

NAME	MSL3 ES	MSL3 FDR q-val	ZNF691 ES	ZNF691 FDR q-val	VPS45 ES	VPS45 FDR q-val
BIOCARTA_WNT_PATHWAY	0.50068516	0.24726693	0.40559503	0.55800956	0.26494917	0.9658848
HALLMARK_WNT_BETA_CATENIN_SIGNALING	0.40808997	0.32803485	0.50642127	0.19558854	0.30884558	1
KEGG_WNT_SIGNALING_PATHWAY	0.35161358	0.21859159	0.29515782	0.5391811	-0.22248736	1
LABBE_TARGETS_OF_TGFB1_AND_WNT3A_UP	0.4382159	0.16877232	-0.43711746	0.10804182	-0.45161828	0.2764436
LABBE_WNT3A_TARGETS_UP	0.38479647	0.26081967	0.26193935	0.7111867	-0.3869322	0.50717163
MORF_WNT1	0.29834503	0.5672574	-0.30149165	0.53103656	-0.36487234	0.58450246
PID_BETA_CATENIN_DEG_PATHWAY	0.53747255	0.21090178	0.47184977	0.4739237	0.3335619	0.95564145
PID_BETA_CATENIN_NUC_PATHWAY	0.45907766	0.15456228	0.29220486	0.61563593	-0.33250704	0.78693783
PID_WNT_CANONICAL_PATHWAY	0.27568805	0.9761062	-0.46176985	0.34014586	0.52560806	0.4529787
PID_WNT_NONCANONICAL_PATHWAY	0.39986426	0.42729422	0.32675785	0.6661658	-0.21913823	0.99517626
PID_WNT_SIGNALING_PATHWAY	0.53348315	0.14695922	0.4910206	0.31809035	-0.30748686	1
REACTOME_SIGNALING_BY_WNT	-0.24062428	0.91663986	0.3499914	0.45545292	0.40496498	0.2813934
SANSOM_WNT_PATHWAY_REQUIRE_MYC	0.6148553	0.002785715	0.5707256	0.008452381	0.31654623	1
ST_WNT_BETA_CATENIN_PATHWAY	0.54881716	0.24948101	0.44606385	0.47015122	-0.27723423	1
ST_WNT_CA2_CYCLIC_GMP_PATHWAY	0.5121965	0.22752231	0.3837594	0.56898904	-0.5685028	0.3297095
WILLERT_WNT_SIGNALING	0.46251318	0.32940158	0.45677087	0.48588544	0.33245826	1
WNT_SIGNALING	0.42880502	0.14114054	0.38952115	0.31522733	-0.30351186	0.85607326
WNT_UP.V1_UP	-0.32198775	0.5471007	-0.41968238	0.21307546	-0.26259094	0.9369174

NAME	ITGB3BP ES	ITGB3BP FDR q-val	TLE2 ES	TLE2 FDR q-val	ZNF498 ES	ZNF498 FDR q-val
BIOCARTA_WNT_PATHWAY	0.27822173	1	0.43097696	0.39346018	-0.30131975	1
HALLMARK_WNT_BETA_CATENIN_SIGNALING	0.33701003	0.9373706	-0.32584423	0.99249893	-0.230408	0.9891348
KEGG_WNT_SIGNALING_PATHWAY	0.34628886	0.67934775	-0.3134428	0.9966895	0.28381112	0.649148
LABBE_TARGETS_OF_TGFB1_AND_WNT3A_UP	0.3360171	0.7487376	-0.33232063	1	0.44040895	0.46758547
LABBE_WNT3A_TARGETS_UP	-0.27526447	0.733577	0.3471952	0.45359927	0.2913364	0.7125552
MORF_WNT1	0.3666987	0.75224817	0.3254184	0.366024	0.41090497	0.47075042
PID_BETA_CATENIN_DEG_PATHWAY	0.30222023	0.97779393	-0.34343016	0.9316789	0.48040065	0.7929425
PID_BETA_CATENIN_NUC_PATHWAY	-0.3441435	0.48604485	0.2898411	0.67021835	0.34396508	0.693355
PID_WNT_CANONICAL_PATHWAY	0.42281795	0.97160846	-0.34575558	0.95642847	0.44357908	0.5533377
PID_WNT_NONCANONICAL_PATHWAY	-0.4309962	0.475984	-0.32898748	1	0.39542663	0.6090444
PID_WNT_SIGNALING_PATHWAY	0.53490955	0.28163618	-0.36698306	1	0.49111214	0.4959272
REACTOME_SIGNALING_BY_WNT	-0.5146342	0.060307786	0.5178634	0.07184874	-0.5555216	0.032171216
SANSOM_WNT_PATHWAY_REQUIRE_MYC	0.5596827	0.06555607	0.43950275	0.2566964	0.2893307	0.8059861
ST_WNT_BETA_CATENIN_PATHWAY	0.32397312	1	0.41881448	0.4143618	0.4130237	0.7180088
ST_WNT_CA2_CYCLIC_GMP_PATHWAY	0.360774	1	-0.54701483	0.38358778	-0.48733875	0.5493748
WILLERT_WNT_SIGNALING	0.33959723	0.9845459	-0.37513247	1	-0.3282184	1
WNT_SIGNALING	0.36339325	0.6808366	-0.28896946	1	-0.30583572	0.89029896
WNT_UP.V1_UP	0.29095387	0.89968854	-0.3841682	0.38784072	-0.3956567	0.27318844

c) Analysis about Notch pathway.

NAME	MSL3 ES	MSL3 FDR q-val	ZNF691 ES	ZNF691 FDR q-val	VPS45 ES	VPS45 FDR q-val
HALLMARK_NOTCH_SIGNALING	0.39970455	0.50415075	-0.30722243	1	0.30123582	1
KEGG_NOTCH_SIGNALING_PATHWAY	0.33367792	0.5833455	-0.32325235	1	0.32301956	1
NGUYEN_NOTCH1_TARGETS_UP	0.5123029	0.38856462	0.36748326	0.7100778	-0.45039618	1

NOTCH_DN.V1_UP	0.33975208	0.42480552	-0.3767208	0.5930178	-0.2829439	0.60900193
PID_NOTCH_PATHWAY	0.5386918	0.07561037	-0.28863004	1	-0.34330997	0.7099428
REACTOME_ACTIVATED_NOTCH1_TRANSMITS_SIGNAL_TO_THE_NUCLEUS	0.45448053	0.41499186	-0.41958913	0.89529645	-0.4239685	0.75111973
REACTOME_NOTCH1_INTRACELLULAR_DOMAIN_REGULATES_TRANSCRIPTION	0.41603547	0.45211473	-0.26974142	0.9276956	-0.26309404	0.92541456
REACTOME_PRE_NOTCH_EXPRESSION_AND_PROCESSING	0.38369974	0.5329762	0.3677576	0.96959716	0.27121586	1
REACTOME_PRE_NOTCH_TRANSCRIPTION_AND_TRANSLATION	0.33194354	0.80433846	0.30659425	0.86089814	0.25901774	0.9667589
REACTOME_SIGNALING_BY_NOTCH	0.39099094	0.3189336	-0.24855095	0.94373643	-0.24031445	0.97611856
REACTOME_SIGNALING_BY_NOTCH1	0.42522132	0.33775452	-0.32079116	1	-0.32878363	0.67414916
VILIMAS_NOTCH1_TARGETS_UP	0.34052703	0.56567496	-0.5524359	0.10098593	-0.50375295	0.32342023

	ITGB3BP		TLE2		ZNF498	
NAME	ES	FDR q-val	ES	FDR q-val	ES	FDR q-val
HALLMARK_NOTCH_SIGNALING	0.31935933	0.83030385	-0.37403646	0.80402285	0.31743002	1
KEGG_NOTCH_SIGNALING_PATHWAY	-0.26978302	0.93780166	-0.3590205	0.71470046	0.38923326	0.72365975
NGUYEN_NOTCH1_TARGETS_UP	-0.5192529	0.9038287	0.47246212	0.3763515	0.50879115	0.5752291
NOTCH_DN.V1_UP	-0.31945366	0.71928084	-0.4105551	0.25005195	0.2687823	1
PID_NOTCH_PATHWAY	0.3203501	0.75950414	-0.28769416	0.8827379	0.2561195	1
REACTOME_ACTIVATED_NOTCH1_TRANSMITS_SIGNAL_TO_THE_NUCLEUS	-0.4180081	0.6673923	-0.5864395	0.19252507	-0.43735707	1
REACTOME_NOTCH1_INTRACELLULAR_DOMAIN_REGULATES_TRANSCRIPTION	-0.2877716	1	-0.3255602	0.81797713	-0.28467304	0.8769092
REACTOME_PRE_NOTCH_EXPRESSION_AND_PROCESSING	0.41334668	0.4481293	0.3826046	0.46158862	0.41669214	0.7750863
REACTOME_PRE_NOTCH_TRANSCRIPTION_AND_TRANSLATION	0.50632274	0.34445304	0.53116864	0.3348121	0.46344584	0.8325777
REACTOME_SIGNALING_BY_NOTCH	-0.2466072	1	-0.33128053	0.8068371	0.27011403	1
REACTOME_SIGNALING_BY_NOTCH1	-0.30381203	1	-0.42900053	0.3763364	-0.27893782	1
VILIMAS_NOTCH1_TARGETS_UP	0.4883157	0.29421756	-0.5600983	0.13145483	-0.36271894	0.72057426

Supplementary Table S4: Primer list that is used in this research

Subcloning at shRNA screen

Forward primer	ATCTCGACGGTATCGATGGCCGCCCCCTTCA
Reverse primer	CATGGTGGCGTCTAGACCAAGTGACGATCACAGCGAT
Primer for sequencing	GACTATCATATGCTTACCGT

Subcloning at construction of LentiORF

ITGB3BP_ORF_Fw	AGACACCGACTCTAGATGCCTTTTGCTCCCGTTG
ITGB3BP_ORF_Rv	CCGCGGGCCCTCTAGGTTTAAAATGGCTTTAAGGAAT
MSL3_ORF_Fw	AGACACCGACTCTAGATGAGCGCGAGCGAGGGC
MSL3_ORF_Rv	CCGCGGGCCCTCTAGATAAATTGCCCGGGGGTTC
ZNF498_ORF_Fw	AGACACCGACTCTAGATGCTTAAAGAGCATCCAGA
ZNF498_ORF_Rv	CCGCGGGCCCTCTAGCTGCACCAGCGGCTCCTG
ZNF691_ORF_Fw	AGACACCGACTCTAGATGGGCAGTGAGAAGGAG
ZNF691_ORF_Rv	CCGCGGGCCCTCTAGGCTGGAATCTTTCCCAGC
TLE2_ORF_fwd	AGA CAC CGA CTC TAG ATG TAC CCC CAG GGA AGG
TLE2_ORF_Rev	CCGCGGGCCCTCTAGAGTAGACCACCTCATACACG
VPS45_ORF_fwd	AGACACCGACTCTAGATGAACGTGGTTTTTGCTGT
VPS45_ORF_Rev	CCGCGGGCCCTCTAGATCTTCTGCTCGCTGACCT

Primer for RT-PCR

MGAT5_Fw	GCTCATCTGCGAGCCTTCT
MGAT5_Rv	TTGGCAGGTCACCTTGTACTT
ZNF691_Fw	CTCTGCTGAGTATTACAGGCATTT
ZNF691_Rv	TCAGGCAGGTGTGGTTCTG
KIAA0101_Fw	CCCAGAAAGGTGCTTGGTT
KIAA0101_Rv	CCTCGATGAAACTGATGTCG
MSL3_Fw	GCCACATGCCAACATGAAC
MSL3_Rv	TCCATCCACCATCTCCTTACA
GIPC2_Fw	CTTCGCCTGAGATCAAAAGG
GIPC2_Rv	ATTGCCTTTGCTTTGGTTTC
ENPP4_Fw	TGTGCTAAATGAATCATCACAAAAAT
ENPP4_Rv	TTGTAGCCTTTGTGAAATGCAG
VPS45_Fw	GCTGAAGCTGATGAACAGGA
VPS45_Rv	TGTGGGTTCACAGCAATGTAA
DUSP7_Fw	TGGACCTAAGAGGGCTGAAA
DUSP7_Rv	TTTCAATAGCTGCCTGTTTGG
RBM15_Fw	TGGGAACCCAATAACAGTGG
RBM15_Rv	TCTTCTACAGTCTCATTGGGAAAA
PPP4R2_Fw	GTGAGGGACTCCGGGAAG
PPP4R2_Rv	TGATCCAGGACAGGACAAACT
STOX2_Fw	GAGAGCCACCCATTCAGC
STOX2_Rv	TGATCTCTACAGGTGCAGAAACAT
LMNA_Fw	GGCTAGCTTCTGCTTTTCTGC
LMNA_Rv	CTGCCTCTCAGGCACCAT
OR10J5_Fw	AGGCTGTGCTACACAAATGTTC
OR10J5_Rv	ATAGCGGTCATACCCCATTG
MYPOP_Fw	CCGCCAAGATCAACGGTAT
MYPOP_Rv	GTTCCAGCGCTTCTGCAC
ATPIF1_Fw	GGGCCTTCGGAAAGAGAG
ATPIF1_Rv	TTCAAAGCTGCCAGTTGTTC
MAST4_Fw	CCAAGAAGAAAGAAAGTCTCGAA

MAST4_Rv	CCGATGACAGGGATCTGTTC
RTN2_Fw	TCAGTCTCGGAACCACGAG
RTN2_Rv	GGGATGCTCTCCAAGCTGT
TNFSF9_Fw	TCAGGCTCCGTTTCACTTG
TNFSF9_Rv	CAGGTCCACGGTCAAAGC
C1orf109_Fw	GACAAGCTAGGGGAAAGGCTA
C1orf109_Rv	TGCTCATAGATCTGAAACACTCG
DBH_Fw	ATGGAGCTGGGACTGGTGTA
DBH_Rv	GTGAGGATGAAGGCGGTCT
C9orf64_Fw	ATTGACAGCGGAGGCGTA
C9orf64_Rv	TTCAGCTCATGAAGGGCTTT
PRKAG2_Fw	GCAAAAGGAAGAAAACTTTGTCA
PRKAG2_Rv	TCCCGGTTCTGGTGTCTC
CBX5_Fw	ATGTTCAGGCCAAACCACAG
CBX5_Rv	AAAATCTCAATAAGAAAATCCCAAAA
FGR_Fw	TGGAAAGATTGGGAGAAAGG
FGR_Rv	CGCTTTCCCGAATGAGAA
RBPMS_Fw	TGTGCCCTTAGAAAGCCCTA
RBPMS_Rv	CACACTTGGCACACAGTTGA
BDKRB1_Fw	CACGGGATGTAATTCAATGGT
BDKRB1_Rv	TGCGTCTCATAAATCAGGTGTC
ITGB3BP_Fw	CCAACAACTGGAACTTGTCAAA
ITGB3BP_Rv	GTTAAACTGGGGTGATTCAATTTT
XPA_Fw	GACACAGGAGGAGGCTTCATT
XPA_Rv	TCGCATATTACATAATCAAATTCCATA
ZNF498_Fw	CGAGGAGCAGCTCAGTCAG
ZNF498_Rv	CCTGCAGAACAGGTAGTGCTT
NDUFB6_Fw	AGAGCTGAGAAGGCGATGG
NDUFB6_Rv	CATAGGCCCCATCTTCTGTG
CSNK2A2_Fw	CCATGGAGCACCCATACTTC
CSNK2A2_Rv	CACAGCATTGTCTGCACAAG
PAN2_Fw	GGGCTTCATTGGCTATGC
PAN2_Rv	TCTGACTCCTTGAGTCTGTAGGG
HYDIN_Fw	ATAACTGAGGAAGTGCCAGAAGAC
HYDIN_Rv	CTCCATCTGGAGATGAGCACT
PPP4R2_Fw	GTG AGG GAC TCC GGG AAG
PPP4R2_Rv	TGA TCC AGG ACA GGA CAA ACT
ODF2L_Fw	CCACGGTGTACCAGTGAAAG
ODF2L_Rv	CCGCTTCCTTAAGTGTTGCTT
ZNF235_Fw	AACCCAAAGAGGTAAGCATTCA
ZNF235_Rv	CTTGATTTGCCAGCATGAAA
FAM175A_Fw	TGAGAAAAGGAGAGGAGCACA
FAM175A_Rv	CCTGAGGGTCTTTTTGGATG
CCT6B_Fw	TGCCATAAGAGATGGACTTCG
CCT6B_Rv	TCAGCCATTGCCACTTCA
CRELD2_Fw	TCCTACACGTGCGAAGAGTG
CRELD2_Rv	TCGCGTAGCCAGAGATACAC
KIF17_Fw	GTGTGAGCACATCATGGAGAC
KIF17_Rv	TTGTTCATCAGCGTGTAGCC
MAPRL16_Fw	CGGAGTTTACAGAAGGCAATTT
MAPRL16_Rv	GATTGTCAGGCGCATCATT
SHMT2_Fw	GGGAGAAGGACAGGCAGTG
SHMT2_Rv	ACCCTCCGAGTACTTGTTGTTC

HS6ST3_Fw	GCCATCATGGAGAAGAAGGA
HS6ST3_Rv	CCCGTAACATTGTGATGTAATAGAA
CES3_Fw	TTCCTCATGGGTGTCAACAA
CES3_Rv	TCATCTGCTCCATTGTATCCAG
MAPRE2_Fw	AAGTCTCACCATGCAAACTCC
MAPRE2_Rv	GGTGTGGATCCTGGTTTAGC
TLE2_Fw	CAGATTATCCCCTTCCTGACC
TLE2_Rv	CTGCTTGGCGCGTTCTAC
KIF11_Fw	GCCATGCTAGAAGTACATAAGACCT
KIF11_Rv	TCCAAGTGCTACTGTAGTAATGGTATC
CTNNBP2_Fw	CCTGAAGCACAGACAAATGG
CTNNBP2_Rv	TTTCACTGGGATCAGACAAGC
CELSR1_Fw	AGTGCTTCGAGGACTTCACTG
CELSR_Rv	AGGAGGTATCAAGGAAAGAGCAT
CYB561D2_Fw	CATCTTTGTGGCTGTGCTTG
CYB561D2_Rv	CAGTAGTGCCTCGGTCATCA
C14orf119_Fw	GGTGCCCTTCGAGAGAAAA
C14orf119_Rv	GGGAAGGATAGTGGCATTGA
IRF9_Fw	ATGCAGGCAAGCAGGACTT
IRF9_Rv	TCCTGTGTCCCCCTCCTTAT
UFM1_Fw	CCCAGATCACCAGGCTTCTA
UFM1_Rv	CACTTTGCTACCCCCTTCTG
CSH1_Fw	TCCCTGCTCCTGGCTTTT
CSH1_Rv	GGAGCATAGCGTGGTCAAA
PTRH2_Fw	AGATCTTTGCAATAAGGAGCTGA
PTRH2_Rv	GCCTCAACGCCTTTACCTCT
SRRM2_Fw	GGCCTAATCCTGACATCCTG
SRRM2_Rv	ATTTGCTGTTCCTCGTACCC
CELA2A_Fw	GTGGTTGGCGGTGAAGAA
CELA2A_Rv	GGAGCTGTACTGCAGGGAGA
ACTB_Fw	CCAACCGCGAGAAGATGA
ACTB_Rv	CCAGAGGCGTACAGGGATAG
MOB2	TaqMan(R) Gene Expression Assays, Inventoried ArrayID Hs00364895_m1
MC5R	TaqMan(R) Gene Expression Assays, Inventoried ArrayID Hs00271882_s1

Supplementary Table S5: TRC shRNA clones used for excluding off-target effect.

gene		TRC shRNA clones
MSL3	sh1	TRCN0000022104
	sh2	TRCN0000022105
	sh3	TRCN0000022106
ZNF691	sh1	TRCN0000020944
	sh2	TRCN0000020948
	sh3	TRCN0000020946
VPS45	sh1	TRCN0000156836
MOB2	sh1	TRCN0000125834
	sh2	TRCN0000125835
	sh3	TRCN0000125836
DUSP7	sh1	TRCN0000007175
	sh2	TRCN0000007176
	sh3	TRCN0000007177
MAST4	sh1	TRCN0000021449
	sh2	TRCN0000021450
	sh3	TRCN0000021452
MGAT5	sh1	TRCN0000036059
	sh2	TRCN0000036061
	sh3	TRCN0000036062
GIPC2	sh1	TRCN0000159402
	sh2	TRCN0000159619
	sh3	TRCN0000159782
KIAA0101	sh1	TRCN0000121547
	sh2	TRCN0000122084
	sh3	TRCN0000143189
ENPP4	sh1	TRCN0000048899
	sh2	TRCN0000048900
ITGB3BP	sh1	TRCN0000057733
	sh2	TRCN0000057734
	sh3	TRCN0000057735
	sh4	TRCN0000057736
TLE2	sh1	TRCN0000019629
	sh2	TRCN0000019630
	sh3	TRCN0000019631
	sh4	TRCN0000019632
ZNF498	sh1	TRCN0000107690
	sh2	TRCN0000107691
	sh3	TRCN0000107692
	sh4	TRCN0000107693
	sh5	TRCN0000107694
PAN2	sh1	TRCN0000004427
	sh2	TRCN0000004428

	sh3	TRCN0000004429
	sh4	TRCN0000004430
	sh5	TRCN0000010875
CSNK2A2	sh1	TRCN0000000611
	sh2	TRCN0000000612
	sh3	TRCN0000000613
	sh4	TRCN0000000614
	sh5	TRCN0000000615
NDUFB6	sh1	TRCN0000028417
	sh2	TRCN0000028448
	sh3	TRCN0000028428
ZNF235	sh1	TRCN0000013388
	sh2	TRCN0000013389
	sh3	TRCN0000013390
	sh4	TRCN0000013392
XPA	sh1	TRCN0000083193
	sh2	TRCN0000083194
	sh3	TRCN0000083195
ODF2L	sh1	TRCN0000121940
	sh2	TRCN0000122238
	sh3	TRCN0000139780
FAM175A	sh1	TRCN0000122051
	sh2	TRCN0000122344
	sh3	TRCN0000139032
HYDIN	sh1	TRCN0000128414
	sh2	TRCN0000128953
	sh3	TRCN0000130229
KIF17	sh1	TRCN0000108345
	sh2	TRCN0000108346
CCT6B	sh1	TRCN0000140500
	sh2	TRCN0000142279
	sh3	TRCN0000142351
CRELD2	sh1	TRCN0000055913
	sh2	TRCN0000055915
	sh3	TRCN0000055916
	sh4	TRCN0000055917
HBA2	sh1	TRCN0000029099
	sh2	TRCN0000029100
	sh3	TRCN0000029101
SHMT2	sh1	TRCN0000034804
	sh2	TRCN0000034805
	sh3	TRCN0000034806
MRPL16	sh1	TRCN0000133992
	sh2	TRCN0000134265
	sh3	TRCN0000135996

CES3	sh1	TRCN0000046713
	sh2	TRCN0000046714
	sh3	TRCN0000046715
HS6ST3	sh1	TRCN0000036359
	sh2	TRCN0000036360
	sh3	TRCN0000036361
MAPRE2	sh1	TRCN0000116507
	sh2	TRCN0000116508
	sh3	TRCN0000116509

Supplementary Table S6: Gene sets used in this research

Gene set about TGF-β pathway
BIOCARTA_TGFB_PATHWAY
COULOUARN_TEMPORAL_TGFB1_SIGNATURE_UP
GSE39820_CTRL_VS_TGFBETA1_IL6_CD4_TCELL_DN
GSE39820_CTRL_VS_TGFBETA1_IL6_IL23A_CD4_TCELL_DN
GSE39820_CTRL_VS_TGFBETA3_IL6_CD4_TCELL_UP
GSE39820_CTRL_VS_TGFBETA3_IL6_IL23A_CD4_TCELL_UP
GSE39820_TGFBETA1_IL6_VS_TGFBETA1_IL6_IL23A_TREATED_CD4_TCELL_UP
GSE39820_TGFBETA1_VS_TGFBETA3_IN_IL6_IL23A_TREATED_CD4_TCELL_UP
GSE39820_TGFBETA1_VS_TGFBETA3_IN_IL6_TREATED_CD4_TCELL_UP
GSE39820_TGFBETA3_IL6_VS_TGFBETA3_IL6_IL23A_TREATED_CD4_TCELL_UP
GSE7460_CTRL_VS_TGFB_TREATED_ACT_CD8_TCELL_UP
GSE7460_CTRL_VS_TGFB_TREATED_ACT_FOXP3_HET_TCONV_UP
GSE7460_CTRL_VS_TGFB_TREATED_ACT_FOXP3_MUT_TCONV_UP
GSE7460_CTRL_VS_TGFB_TREATED_ACT_TCONV_UP
GSE7460_CTRL_VS_TGFB_TREATED_ACT_TREG_UP
GSE7460_FOXP3_MUT_VS_WT_ACT_WITH_TGFB_TCONV_UP
GSE7460_TREG_VS_TCONV_ACT_WITH_TGFB_UP
GSE7460_WT_VS_FOXP3_HET_ACT_WITH_TGFB_TCONV_UP
HALLMARK_TGF_BETA_SIGNALING
JAZAG_TGFB1_SIGNALING_UP
JAZAG_TGFB1_SIGNALING_VIA_SMAD4_UP
KARAKAS_TGFB1_SIGNALING
KARLSSON_TGFB1_TARGETS_UP
KEGG_TGF_BETA_SIGNALING_PATHWAY
LABBE_TARGETS_OF_TGFB1_AND_WNT3A_UP
LABBE_TGFB1_TARGETS_UP
MCBRYAN_PUBERTAL_TGFB1_TARGETS_UP
PID_TGFBR_PATHWAY
PLASARI_TGFB1_SIGNALING_VIA_NFIC_10HR_UP
PLASARI_TGFB1_SIGNALING_VIA_NFIC_1HR_UP
PLASARI_TGFB1_TARGETS_10HR_UP
PLASARI_TGFB1_TARGETS_1HR_UP
REACTOME_DOWNREGULATION_OF_TGF_BETA_RECEPTOR_SIGNALING
REACTOME_SIGNALING_BY_TGF_BETA_RECEPTOR_COMPLEX
REACTOME_TGF_BETA_RECEPTOR_SIGNALING_ACTIVATES_SMADS
TGFB_UP.V1_UP
TRANSFORMING_GROWTH_FACTOR_BETA_RECEPTOR_SIGNALING_PATHWAY
VERRECCHIA_DELAYED_RESPONSE_TO_TGFB1
VERRECCHIA_EARLY_RESPONSE_TO_TGFB1
VERRECCHIA_RESPONSE_TO_TGFB1_C1
VERRECCHIA_RESPONSE_TO_TGFB1_C2
VERRECCHIA_RESPONSE_TO_TGFB1_C5

Gene set about Wnt pathway
BIOCARTA_WNT_PATHWAY
HALLMARK_WNT_BETA_CATENIN_SIGNALING
KEGG_WNT_SIGNALING_PATHWAY
LABBE_TARGETS_OF_TGFB1_AND_WNT3A_UP
LABBE_WNT3A_TARGETS_UP
MORF_WNT1
PID_BETA_CATENIN_DEG_PATHWAY
PID_BETA_CATENIN_NUC_PATHWAY
PID_WNT_CANONICAL_PATHWAY
PID_WNT_NONCANONICAL_PATHWAY
PID_WNT_SIGNALING_PATHWAY
REACTOME_SIGNALING_BY_WNT
SANSOM_WNT_PATHWAY_REQUIRE_MYC
ST_WNT_BETA_CATENIN_PATHWAY
ST_WNT_CA2_CYCLIC_GMP_PATHWAY
WILLERT_WNT_SIGNALING
WNT_SIGNALING
WNT_UP.V1_UP

Gene set about Notch pathway
HALLMARK_NOTCH_SIGNALING
KEGG_NOTCH_SIGNALING_PATHWAY
NGUYEN_NOTCH1_TARGETS_UP
NOTCH_DN.V1_UP
PID_NOTCH_PATHWAY
REACTOME_ACTIVATED_NOTCH1_TRANSMITS_SIGNAL_TO_THE_NUCLEUS
REACTOME_NOTCH1_INTRACELLULAR_DOMAIN_REGULATES_TRANSCRIPTION
REACTOME_PRE_NOTCH_EXPRESSION_AND_PROCESSING
REACTOME_PRE_NOTCH_TRANSCRIPTION_AND_TRANSLATION
REACTOME_SIGNALING_BY_NOTCH
REACTOME_SIGNALING_BY_NOTCH1
VILIMAS_NOTCH1_TARGETS_UP

Gene set about Hedgehog pathway
HALLMARK_HEDGEHOG_SIGNALING
KEGG_HEDGEHOG_SIGNALING_PATHWAY
PID_HEDGEHOG_2PATHWAY
PID_HEDGEHOG_GLI_PATHWAY
YAUCH_HEDGEHOG_SIGNALING_PARACRINE_UP

Supplementary Material and Method

Stable knockdown and overexpression of target genes

To establish stable cells that overexpress some target genes, we generated the lentiviral open reading frame (ORF) plasmids by editing the LentiORF plasmids (GE Healthcare Life Science).

First, we purchased the ORF of the target genes from Kazusa RNA Res Inst. Chiba, Japan. We amplified the ORFs by PCR. Cycling parameters were 98°C for 5 minutes, followed by 30 cycles of 98°C for 10 seconds, 63°C for 10 seconds and 72°C for 30 seconds, followed by 72°C for 10 minutes. As a cloning vector, we enzymatically cut precision LentiORF at the XbaI site. We subcloned PCR amplicons into the cloning vector using the InFusion Kit as recommended by the manufacturer, thus generating the lentiviral ORF plasmids. We transfected these into cells as recommended by the manufacturer. Stably transfected cells were selected with blasticidin (10 µg/ml, Thermo Fisher Scientific). All primers are listed in Table S4 and were purchased from Greiner.

Gene expression microarray datasets and copy number dataset

Microarray datasets were obtained from the Gene Expression Omnibus website (http://www.ncbi.nlm.nih.gov/geo). The International Cancer Genome Consortium dataset includes 111 samples from 94 HGSOC patients. RNAseq data and patient information were downloaded from the ICGC website. We used datasets GSE2109, TCGA, and GSE3149 to analyze overall survival. To analyze mRNA expression of the primary site and disseminated site, we used the GSE2109 dataset

which contained 75 primary HGSOCs and 43 disseminated HGSOC tumor samples. To analyze the influence of chemotherapy, we used the ICGC and GSE15622 datasets. The ICGC dataset contains 17 paired samples obtained before and after chemotherapy. GSE15622 is a gene expression microarray dataset composed of laparoscopic biopsy specimens from ovarian cancers that were subsequently treated with paclitaxel monotherapy (n=20) or carboplatin monotherapy (n=15). Among them, six patients showed resistance to paclitaxel. To analyze the copy number alterations in the HGSOC samples in TCGA, the cBioPortal for Cancer Genomics database (http://www.cbioportal.org/public-portal/) was used.

RNA sequencing

Library preparation and sequencing

mRNA was converted into a library of template molecules suitable for subsequent cluster generation using the Illumina TruSeq RNA Sample Preparation Kit v2. The first step in the workflow involves purifying the poly-A containing mRNA molecules using poly-T oligo-attached magnetic beads. Following purification, the mRNA is fragmented into small pieces using divalent cations under elevated temperature. The cleaved RNA fragments are copied into first strand cDNA using reverse transcriptase and random primers. This is followed by second strand cDNA synthesis using DNA Polymerase I and RNase H. These cDNA fragments then go through an end repair process, the addition of a single 'A' base, and then ligation of the adapters. The products are then

purified and enriched with PCR to create the final cDNA library. The libraries were sequenced on an Illumina HiSeq 2500 platform in paired-end 100 bp configuration.

Transcriptome analysis

Adapter and low-quality sequences were removed by cutadapt (v1.2.1) (1). After quality control, poly-A/T sequences were also removed by PRINSEQ (v0.19.2) (2). For gene expression analysis, the trimmed reads were aligned to the reference human genome (GRCh37/hg19) using TopHat (v2.0.13) (3). Mapped reads were assembled by Cufflinks (v2.2.1) (4), and the transcripts across all samples were merged by Cuffmerge, a part of the Cufflinks package. The “fragments per kilobase per million map reads” (FPKM) was calculated with Cuffquant, and differential expression analysis (calculating fold change and testing the statistical significance) between samples was performed with Cuffdiff (Cuffquant and Cuffdiff are programs involved in the Cufflinks package).

Western Blotting

Nuclear protein was extracted using NE-PER Nuclear and Cytoplasmic Extraction Reagents (Thermo Fisher Scientific) according to the manufacturer’s protocol. Western blotting was performed as previously described[13]. Primary antibodies included anti-phospho smad3C rabbit polyclonal antibody (1:1000, Immuno-Biology Laboratories, Gunma, Japan), anti-beta catenin rabbit monoclonal antibody (1:1,000, Cell Signal Technology, Danver, USA), anti-TBP rabbit monoclonal antibody (1:1000, Abcam, Cambridge, UK), and anti-GAPDH mouse monoclonal antibody (1:1000,

Abcam). After washing in tris-buffered saline (TBS)-T, the blots were incubated with the appropriate peroxidase-coupled secondary antibody (1:2000; anti-rabbit HRP or anti-mouse HRP, GE Healthcare Life Science). Specific proteins were detected using ECL Plus Western Blotting Reagent (GE Healthcare Life Science). The bands were visualized using the Molecular Imager Gel DocTMXR+ and ChemiDocTMXRS+ Systems with Image Lab 2.0 software (Bio-Rad).

References

1. Martin M. Cutadapt removes adapter sequences from high-throughput sequencing reads. *EMBnetjournal, North America* 2011; **17**: 10-12.

2. Schmieder R, Edwards R. Quality control and preprocessing of metagenomic datasets. *Bioinformatics* 2011; **27**: 863-864.

3. Trapnell C, Pachter L, Salzberg SL. TopHat: discovering splice junctions with RNA-Seq. *Bioinformatics* 2009; **25**: 1105-1111.

4. Trapnell C, Williams BA, Pertra G, Mortazavi A, Kwan G, van Baren MJ, *et al.* Transcript assembly and quantification by RNA-Seq reveals unannotated transcripts and isoform switching during cell differentiation. *Nat. Biotechnol.* 2010; **28**: 511-515.
